# Sweetener System Intervention Shifted Neutrophils from Homeostasis to Priming

**DOI:** 10.3390/nu15051260

**Published:** 2023-03-02

**Authors:** Thomas Skurk, Tamara Krämer, Patrick Marcinek, Agne Malki, Roman Lang, Andreas Dunkel, Tiffany Krautwurst, Thomas F. Hofmann, Dietmar Krautwurst

**Affiliations:** 1 ZIEL Institute for Food and Health, Core Facility Human Studies, TUM School for Life Sciences Weihenstephan, Technical University of Munich, 85354 Freising, Germany; 2 Leibniz Institute for Food Systems Biology, Technical University of Munich, 85354 Freising, Germany; 3 TUM School of Life Sciences, Technical University of Munich, 85354 Freising, Germany; 4 Chair of Food Chemistry and Molecular Sensory Science, Technical University of Munich, 85354 Freising, Germany

**Keywords:** saccharin, acesulfame-K, cyclamate, taste receptors, cytokines, chemokines, GPCR, polymorphonuclear neutrophils, healthy subjects, chemoreception

## Abstract

Background: Non-nutritive sweeteners (NNS) are part of personalized nutrition strategies supporting healthy glycemic control. In contrast, the consumption of non-nutritive sweeteners has been related to person-specific and microbiome-dependent glycemic impairments. Reports on the effects of NNS on our highly individual cellular immune system are sparse. The recent identification of taste receptor expression in a variety of immune cells, however, suggested their immune-modulatory relevance. Methods: We studied the influence of a beverage-typical NNS system on the transcriptional profiling of sweetener-cognate taste receptors, selected cytokines and their receptors, and on Ca^2+^ signaling in isolated blood neutrophils. We determined plasma concentrations of saccharin, acesulfame-K, and cyclamate by HPLC-MS/MS, upon ingestion of a soft drink-typical sweetener surrogate. In an open-labeled, randomized intervention study, we determined pre- versus post-intervention transcript levels by RT-qPCR of sweetener-cognate taste receptors and immune factors. Results: Here we show that the consumption of a food-typical sweetener system modulated the gene expression of cognate taste receptors and induced the transcriptional regulation signatures of early homeostasis- and late receptor/signaling- and inflammation-related genes in blood neutrophils, shifting their transcriptional profile from homeostasis to priming. Notably, sweeteners at postprandial plasma concentrations facilitated fMLF (*N*-formyl-Met-Leu-Phe)-induced Ca^2+^ signaling. Conclusions: Our results support the notion of sweeteners priming neutrophils to higher alertness towards their adequate stimuli.

## 1. Introduction

Due to the high processing of food and its omnipresence, today’s nutrition contains an increasing number of compounds. As an example, the obesity pandemic might, in part, be caused by a continuously increasing use of added sugars and sweeteners in daily nutrition [1,2]. In recent decades, the use of non-nutritive sweeteners (NNS) [3] has largely increased [4,5] as one strategy to prevent a chronic positive energy balance and excessive weight gain [6,7,8]. Such compounds, which determine the chemosensory properties of foods, are nowadays recognized as bioactives in a more general sense [9,10,11,12,13,14,15,16,17]. In particular, the bioavailability and physiological effects of non-metabolizable NNS have long been under debate [18,19,20,21,22,23,24,25,26]. Beyond their function as adequate, attractive stimuli of our canonical chemical sense of taste, sugars and sweeteners are sensed via their cognate receptors at many locations elsewhere in the body [22,27,28], for example, in the gastrointestinal system [29]. Indeed, a recent study demonstrated that the preference for sugar over sweeteners depended on their differential sensing by duodenal neuropod cells [30]. Moreover, another recent study reported a significantly elevated glycemic response in healthy individuals during the short-term consumption of saccharin and sucralose through interaction with the gut microbiome and its metabolic response [31]. These results suggested highly personalized responses of the human microbiome to the consumption of certain NNS.

Non-nutritive sweeteners via their cognate taste receptors were recently shown also to modulate our cellular immune system [13,14], which is highly variable among individuals [32]. The experimental evidence of such modulation of immune cell functions is commonly obtained from testing the effects of substrates, e.g., sweeteners, on cytokine expression or secretion in isolated primary blood leukocytes. For example, in whole blood cultures, artificial sweeteners were reported to elicit a suppressive effect on IL6 secretion [15]. In particular, saccharin derivatives have recently been demonstrated to inhibit the synthesis of TNF-α and IL8 in THP-1 monocytes, suggesting an anti-inflammatory activity [33]. While in a previous study, food-typical concentrations of saccharin did not induce oxidative or inflammatory stress in circulating mononuclear cells [34], its consumption, however, has been associated recently with impaired glucose tolerance and compromised GLP-1 secretion in rodent models and humans [35,36,37]. Despite uncertainty on the health effects of artificial sweeteners, the development of inflammation-modulating, saccharin-based antagonists of interferon signaling is remarkable [11].

An early review from 1980 concluded that aspartame had no impact on inflammation, at least in animal models [38]. A recent review, however, pointed out that the consumption of aspartame may, indeed, ultimately lead to systemic inflammation [39,40].

Cyclamate is banned in North America [41,42,43], but is widely used in other Western countries, typically in combination with other NNS, such as saccharin, for example in diet soft drinks [44]. Non-nutritive sweeteners may elicit a bitter off-taste as a function of concentration, by the activation of selective bitter taste receptors [45,46,47,48]. The mechanism by which NNS blends become perceptually superior to single compounds has been suggested by a recent study, at least for saccharin and cyclamate, as the mutual suppression of their respective bitter receptors [49].

Beyond the chemical sense of taste, however, there is a need for more information on the bioavailability and common plasma concentrations of food-borne flavor compounds [50], such as sweeteners in particular, and on their molecular effects on potential targets such as peripheral blood immune cells. Recently, we have identified mRNA expression for some eighty chemosensory G protein-coupled receptors (GPCRs) as genuine biomarkers for subpopulations of circulating leukocytes, including the sweet taste receptor dimer and all bitter taste receptors [9,14,51]. Moreover, we demonstrated the functional expression of saccharin-specific sweet and bitter taste receptors in neutrophils [14].

Here, we describe an open-labeled, placebo-controlled, randomized intervention study determining the plasma concentration and biokinetics of the beverage-typical sweeteners saccharin, cyclamate, and acesulfame-K, dissolved in water and analyzed by HPLC-MS/MS. By means of RT-qPCR, we interrogated sweeteners’ effects on the transcriptional regulation of sweetener-specific chemosensory GPCRs and of certain cytokines and their receptors in isolated neutrophils from buffy coat samples in vitro, and from the intervention study-derived blood samples ex vivo. We further investigated (i) by Ca^2+^-fluorimetric experiments, whether a diet lemonade-typical NNS mixture can induce intracellular Ca^2+^ signaling, and (ii) by laser-guided, fluorescence-activated cell analysis, whether the same NNS mixture can modulate fMLF-induced Ca^2+^ signaling in isolated human neutrophils.

## 2. Materials and Methods

### 2.1. Reagents

Calcium buffer was composed of 140 mM NaCl (Carl Roth, Karlsruhe, Germany), 20 mM HEPES (VWR, Radnor, PA, USA), 5 mM KCl (Sigma-Aldrich, St. Louis, MO, USA), 1.8 mM CaCl_2_ (Sigma-Aldrich, St. Louis, MO, USA), and 0.5 mM *D*-glucose (VWR, Radnor, PA, USA), adjusted pH 7.4. The EGTA buffer was prepared according to the calcium buffer, but contained 0.5 mM EGTA (Sigma-Aldrich, St. Louis, MO, USA) instead of CaCl_2_. Probenecid was purchased from Sigma-Aldrich (St. Louis, MO, USA). Sodium hydroxide (NaOH) and dimethyl sulfoxide (DMSO) were both purchased from VWR (Radnor, PA, USA). Pluronic^®^ F-127 was acquired from AAT Bioquest Inc. (Sunnyvale, CA, USA) and dissolved in H_2_O (10% solution). Fura-8 AM was obtained from PromoCell GmbH (Heidelberg, Germany). Thapsigargin was acquired from Cell Signaling Technology (Cambridge, UK). To prepare an aqueous solution of physiological sweetener mix, sodium cyclamate (Thermo Fisher Scientific, Waltham, MA, USA), saccharin (VWR, Radnor, PA, USA), and acesulfame-K (Cayman Chemical, Ann Arbor, MI, USA) were purchased. Fluo-4 AM was acquired from Bio-Techne (Minneapolis, MN, USA). *N*-Formyl-Methionyl-Leucyl-Phenylalanine (fMLF) was obtained from Tokyo Chemical Industry (Tokyo, Japan). Lactisole was purchased from Cayman Chemical (Ann Arbor, MI, USA). DPBS (Genaxxon bioscience GmbH, Ulm, Germany) was used for cell purification. Unless otherwise stated, reagents were initially dissolved in DMSO.

### 2.2. Trial Design and Subjects

The study was designed as a pilot in a cross-over design to address whether the ingestion of typical amounts of sweeteners may modulate the transcription of chemosensory GPCRs or cytokines and their receptors in isolated neutrophils.

**Figure 1:**
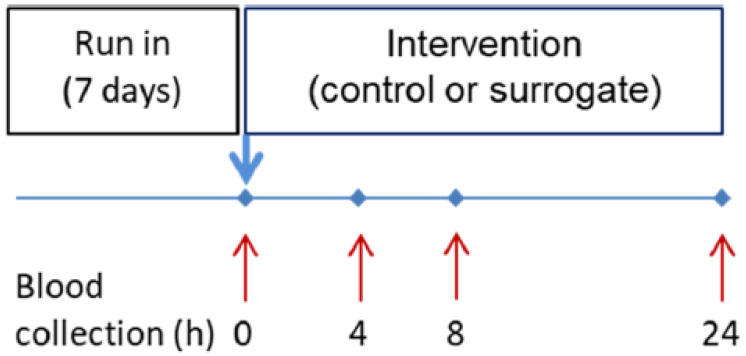
Schematic of the intervention study. After a run-in period without sweetener intake (7 d), study participants consumed a recombination of sweeteners (surrogate) or mineral water (control). Intervention period was 24 h.

The primary and secondary endpoints of our intervention study were the quantification of plasma levels of sweeteners, the quantification of transcript levels of chemosensory GPCRs or cytokines and their receptors in isolated neutrophils, and the statistical analysis of the intervention effects as compared to the water intervention control. These and other analyses not prespecified are considered exploratory. The primary and secondary endpoints of our intervention study did not change during the research or post hoc analyses. The human intervention study was conducted in accordance with the Declaration of Helsinki, and approved by the ethical commission of the faculty of medicine at the Technical University of Munich, Germany (#5798/13, approved on 27 June 2013), and was registered in the WHO partner register DRKS (#DRKS00005083). The study was performed at the ZIEL Institute for Food and Health, Human Study Center of the Else Kröner-Fresenius-Center of Nutritional Medicine (Technical University of Munich, Germany) during July and August 2013. Five female (age range 27–32 yrs, bw 60.3 ± 6.5 kg) and five male (age range 30–47 yrs, bw 80.6 ± 3.5 kg) healthy, non-smoking volunteers were asked to participate in a study consuming either mineral water alone or a combination of saccharin (75.6 mg/L, sodium salt, 0.37 mM), acesulfame-K (52.7 mg/L, potassium salt, 0.27 mM), and cyclamate (227.7 mg/L, sodium salt, 1.13 mM), frequently used in commercially available lemonades, dissolved in mineral water on 2 different occasions. The study was performed after a 7 day run-in period, strictly avoiding non-nutritional sweetener-containing foods (chewing gum, sugar-sweetened beverages, etc.) (Figure 1). To reduce bias, volunteers were randomly allocated by SNOSE (sequentially numbered, opaque single envelopes) to receive either mineral water or the surrogate. Envelopes were prepared by a co-worker from the study center not directly involved in the study conductance. Due to the difference in the sweetness of the test drinks, the study could not be blinded.

After overnight fasting, volunteers were invited to the study center, a venous catheter was inserted, and a baseline blood sample (0 h) was taken; thereafter, either mineral water (10.7 mL/kg bw) as a control, or the mixture of chemically defined sweeteners in mineral water (10.7 mL/kg bw) was given. The volume of the test beverage was calculated to standardize the intake to yield a saccharin concentration of 0.8 mg/kg bw, which is equivalent to commercially available products. Test beverages had to be ingested within 15 min. On test days, blood samples for the isolation of PMNs (see below) or analytics were taken after 0, 4, 8, and 24 h (Figure 1). For the isolation of PMNs, we used EDTA (1 mg/mL) tubes (9 mL), which were immediately transferred to the wet lab and subjected to Ficoll density gradient centrifugation (see below). Those samples for analytics were immediately centrifuged at 3000× *g* for 10 min at 4 °C, were subsequently aliquoted (500 μL portions), and stored at −80 °C. The tested drinks caused no side effects; all ten participants completed the intervention study and were used for analysis.

### 2.3. Human Blood Polymorphonuclear Neutrophil (PMN) Purification

Human blood polymorphonuclear neutrophils were isolated from buffy coat samples (Bavarian Red Cross Blood Bank, or Sonnen-Gesundheitszentrum, Dr. Gerd Becker, Munich, Germany), or immediately isolated from the samples coming out of the intervention study (9 mL EDTA tubes), and purified using Ficoll density gradient centrifugation to at least 90% purity as confirmed by flow cytometry (MACSQuant Analyzer 10, Miltenyi Biotec, Bergisch Gladbach, Germany), as described previously [9]. For in vitro stimulation experiments with sweeteners, cells were resuspended to 1 × 10^6^/mL in RPMI 1640, and then were used for RNA isolation.

For Ca^2+^ fluorimetric experiments, 30 mL of buffy coat samples were mixed with 5 mL of pre-warmed DPBS. Human blood PMNs were isolated using MACSxpress^®^ Whole Blood Neutrophil Isolation Kit, followed by MACSxpress™ Erythrocyte Depletion Kit (Miltenyi Biotec, Bergisch Gladbach, Germany), according to the manufacturers’ recommendations.

### 2.4. RNA Isolation and cDNA Synthesis

Total RNA from blood neutrophils was isolated and purified using the RNeasy Mini Kit with an on-column DNAse-I digest (Qiagen, Hilden, Germany), to avoid genomic DNA contamination. The quality of RNA was analyzed with an Experion™ automated electrophoresis system (Bio-Rad, Hercules, CA, USA), using standard sense RNA analysis LabChips, according to manufacturer’s instructions. The quality of tested RNA samples was determined by the RNA quality index (RQI), which ranged between 7.5 and 10 on a scale from 0–10. cDNA was synthesized from 300 ng total RNA using iScript cDNA Synthesis Kit (Bio-Rad, Hercules, CA, USA), following the manufacturer’s recommendations.

### 2.5. RT-qPCR

The RNA expression levels of investigated genes in PMNS were quantitatively compared by Reverse Transcription-quantitative Polymerase Chain Reaction (RT-qPCR) as relative expression, normalized to an average of 3 selected and stably expressed reference genes (GAPDH, RPL13A, and ACTB), according to the MIQE guidelines [52]. Further normalization was performed to the respective reference gene-controlled lowest RNA expression level, or untreated control, which was set as 1. All RT-qPCR reactions on human PMNS cDNA were performed using GoTaq qPCR master mix (Promega, Madison, WI, USA) in an Mx3000P cycler (Stratagene, Santa Clara, CA, USA): 95 °C (3 min), 40× (95 °C (15 s), 58 °C (30 s), 72 °C (30 s)). The cycle of quantification (Cq) data was evaluated using MxPro software (Stratagene, Santa Clara, CA, USA), and Cq values ≥ 38 were considered as negative. Relative quantification (RQ) of mRNA expression levels of 84 neutrophil-related cytokines and their receptors, as well as other immune cell activation and transcription factor regulation markers (Table S1), were investigated by RT-qPCR using a customized RT^2^ Profiler PCR array (Sabiosciences, Qiagen, Valencia, CA, USA), as described previously [9]. All 48 cognate taste receptor genes, as well as immune-related and neutrophil function-associated genes investigated during sweetener mix intervention, are listed in Table S2.

### 2.6. Quantification of Artificial Sweeteners in Human Plasma

Standard solutions: Stock solutions of the analytes (roughly 10 mM) were individually prepared by dissolving the exactly weighed solids of acesulfame-K, sodium saccharin and sodium cyclamate in methanol. The internal standard p-toluolsulfonic acid hydrate was prepared in acetonitrile at a concentration of ~10 mM, and the exact concentration was determined by qNMR versus a caffeine standard [53]. The internal standard was diluted with acetonitrile (1 + 9999; *v*/*v*) to obtain an internal standard working solution of 1 µM concentration.

Matrix-matched calibration: For matrix-matched calibration, we prepared spiked plasma samples, which were processed and analyzed in triplicates to establish calibration curves, as described in detail in the Supplemental Material (Figure S1).

Quantitative analysis: An aliquot of the plasma sample (100 µL) was mixed with the internal standard working solution (1 mL) in an Eppendorf cup. The mixture was vortexed and centrifuged (12,500 rpm, 4 °C, and 15 min). The clear supernatant was decanted into a new reaction tube and was evaporated in a stream of nitrogen. The residue was taken up in a mixture of water and acetonitrile (95 + 5, *v*/*v*, 100 µL). Aliquots (1 µL) were injected into the HPLC-MS/MS system.

Chromatographic separation: The samples were injected onto a Luna phenylhexyl column (150 × 2 mm, 3 µ, Phenomenex Darmstadt, Germany) protected by a guard column of the same material (2 × 2 mm, 3 µ, Phenomenex, Darmstadt, Germany). Eluents were 1% formic acid in acetonitrile (eluent A) and 1% formic acid in water (eluent B). The solvent was delivered at a flow of 300 µL/min. After 2 min of isocratic elution (A/B 5/95), the composition was changed to A/B 95/5 within 3 min following a non-linear gradient. After 1 min of isocratic elution, the starting conditions were re-established within 0.2 min and kept constant (3.8 min) for equilibration.

Instrumentation: MS/MS data were acquired on a 3200 triple quadrupole mass spectrometer (ABSciex, Darmstadt, Germany). Potentials applied for the introduction of the compounds into the ion source (Q1 mass, declustering potential) and the fragmentation-related parameters (cell entrance potential, collision energy, cell exit potential) were optimized by using the auto-tune function of the software (Analyst 1.5.1, ABSciex, Darmstadt, Germany). The Q1/Q3 pairs are given in Table S3. Details are given in the Supplemental Material. In-house validation was performed by spiking analyte-free human plasma with known amounts of the analytes and subsequent analysis (Table S4). Accuracy ranged between 91.5 and 116.8% for all analytes, and precision from 0.5 to 9.5% RSD. The determined post-prandial concentrations of sweeteners in human plasma at all intervention times are given in Table 1.

### 2.7. Gene Ontology (GO) Term-Based Network and Cluster Analysis

Sets of sweetener intervention-regulated VIP-gene transcripts at the respective post-intervention times were analyzed with respect to functional networks and their underlying GO terms, using the network analyzing software apps Bingo (v3.0.3) and ClueGo (v2.5.5) [54] in Cytoscape (v3.7.1) [55]. The BiNGO analysis was performed using the hypergeometric test and the Benjamini and Hochberg False Discovery Rate (FDR) correction with a chosen significance level of 0.00001. The main ontology subcategory for which the gene sets were tested was “GO_Biological_Process”. The node size reflects the number of genes annotated to a node, multiplied by the normalized sum of the VIP-values of the genes annotated, with the lowest sum set to 1. The node coloring represents the corrected *p*-value. The white nodes, although not overrepresented, are the parental nodes of overrepresented categories down the path, whereas the yellow to orange nodes display the growing overrepresented nodes [56]. Additionally, for each VIP-gene list at the respective post-intervention time, a ClueGO cluster analysis was performed using the settings “GO_BiologicalProcess” and “GO_ImmuneSystemProcess”, referring to the database UniProt [57].

### 2.8. Spectrofluorimetric Ca^2+^ Influx Kinetics in Isolated PMNs In Vitro

Four Mio PMNs/mL were centrifuged at 300× *g* for 10 min. The cell pellet was resuspended in 1 mL calcium buffer, supplemented with final concentrations of 1 mM probenecid and 0.04% Pluronic^®^ F-127. Probenecid was used to block organic anion transport, thereby reducing the leakage of Ca^2+^ dyes Fura-8 AM or Fluo-4 AM from neutrophils [58,59], while Pluronic^®^ F-127 was used to facilitate the solubilization of water-insoluble dyes and to help disperse acetoxymethyl (AM) esters. A total of 4 µM of Fura-8 AM was added, and the cell suspension was incubated for 45 min at 37 °C and 5% CO_2_. The cell suspension was washed twice with calcium buffer or EGTA buffer, respectively. After a final centrifugation step at 300× *g* for 10 min, the cell pellet was resuspended in 2 mL calcium buffer or EGTA buffer, both containing 1 mM probenecid. Fluorescence was measured by a SAFAS Flx-Xenius Spectrofluorometer (SAFAS-Société Anonyme de Fabrication d’Appareillages Scientifiques, Monaco), using 340 nm and 410 nm as dual excitation wavelength and 520 nm as single emission wavelength. Settings were adjusted as follows: integration time, 1 s; bandwidth at excitation and emission, 10 nm; complete filtering; cadence, 50 s. The PMT voltage was set manually to achieve around 50% emissivity for both excitation wavelengths. For all fluorescence measurements, samples contained less than 0.1% DMSO to ensure cell viability. After 2 min and 10 min, different reagents were added, including 1 µM thapsigargin and 30× of physiological sweetener mix (30 µM sodium cyclamate, 21 µM saccharin, 9 µM acesulfame-K). The ratio of F520_340_/F520_410_ was calculated and normalized to the initial ratio at 0 s by using Excel (Microsoft Office Professional 2016, Microsoft Corporation, Redmond, WA, USA). Plots were generated and statistics were performed by using SigmaPlot 14.0 (Systat Software GmbH distributed from Inpixon GmbH, Düsseldorf, Germany). Significances were calculated by paired, two-tailed Student’s *t*-test. Data are shown as mean ± SD (*n* = 3).

### 2.9. Ca^2+^ Signaling Measured by Laser-Guided Flow Cytometry

A total of 0.5 Mio PMNs/mL were centrifuged at 300× *g* for 10 min. The cell pellet was resuspended in 250 µL calcium buffer, supplemented with final concentrations of 1 mM probenecid, 0.04% Pluronic^®^ F-127, and 4 µM of Fluo-4 AM. The cell suspension was incubated for 45 min at 37 °C and 5% CO_2_. The cell suspension was washed twice with calcium buffer. After a final centrifugation step at 300× *g* for 10 min, the cell pellet was resuspended in 500 µL MACSQuant^®^ Running Buffer (Miltenyi Biotec, Bergisch Gladbach, Germany). Cells were pre-stimulated with 1× of physiological sweetener mix (1 µM sodium cyclamate, 0.7 µM saccharin, 0.3 µM acesulfame-K) for 10 min, followed by the addition of fMLF with concentrations ranging from 0.1 nM to 20 nM, with sweeteners being present throughout. For testing an allosteric inhibition of TAS1R2/TAS1R3, lactisole (500 µM) was added to the sweetener mix during pre-stimulation of the cells. Fluorescence was measured until 10,000 events were obtained by laser-guided flow cytometry using MACSQuant^®^ Analyzer 16 (Miltenyi Biotec, Bergisch Gladbach, Germany). Wavelengths were 494 nm for excitation and 506 nm for emission. For flow cytometry, reagents were diluted in MACSQuant^®^ Running Buffer, containing less than 0.1% DMSO to ensure cell viability. Data were analyzed by using Excel (Microsoft Office Professional 2016, Microsoft Corporation, Redmond, WA, USA) and SigmaPlot 14.0 (Systat Software GmbH distributed by Inpixon GmbH, Düsseldorf, Germany).

*EC*_50_ values and curves were derived from fitting the function

$$f\left(x\right)=\left(\frac{\left(min-max\right)}{\left(1+{\left(\frac{x}{EC_{50}}\right)}^{Hillslope}\right)}\right)+max$$

to the data by nonlinear regression (SigmaPlot 14.0, Systat Software). A total of 0.1% DMSO was used as negative control and subtracted from each measurement, using a 5% gate as false positive signal. Significances were calculated by a two-tailed Student’s *t*-test.

### 2.10. Statistical Analysis

Normality Testing (Shapiro–Wilk), paired Student’s *t*-test, or Wilcoxon Signed Rank Test for the in vitro data was performed with SigmaPlot 14.0 (Systat Software GmbH distributed from Inpixon GmbH, Düsseldorf, Germany). RT^2^ Profiler PCR array (Sabiosciences, Qiagen, Valencia, CA, USA) data were analyzed according to the manufacturer’s specifications [60]. Statistical comparisons for the primary outcome measures of the human intervention study were between the sweetener-supplemented test drink and water alone. Data from the intervention study were analyzed with R (version 3.5.2) using packages tidyverse [61], ropls [62], and cowplot [63]. Statistical evaluation of group differences was achieved employing multivariate analysis by orthogonal partial least square discrimination analysis (OPLS-DA) [64]. 

## 3. Results

### 3.1. Saccharin Upregulated Sweet Taste Receptor Gene Expression in Isolated Neutrophils In Vitro

**Figure 2:**
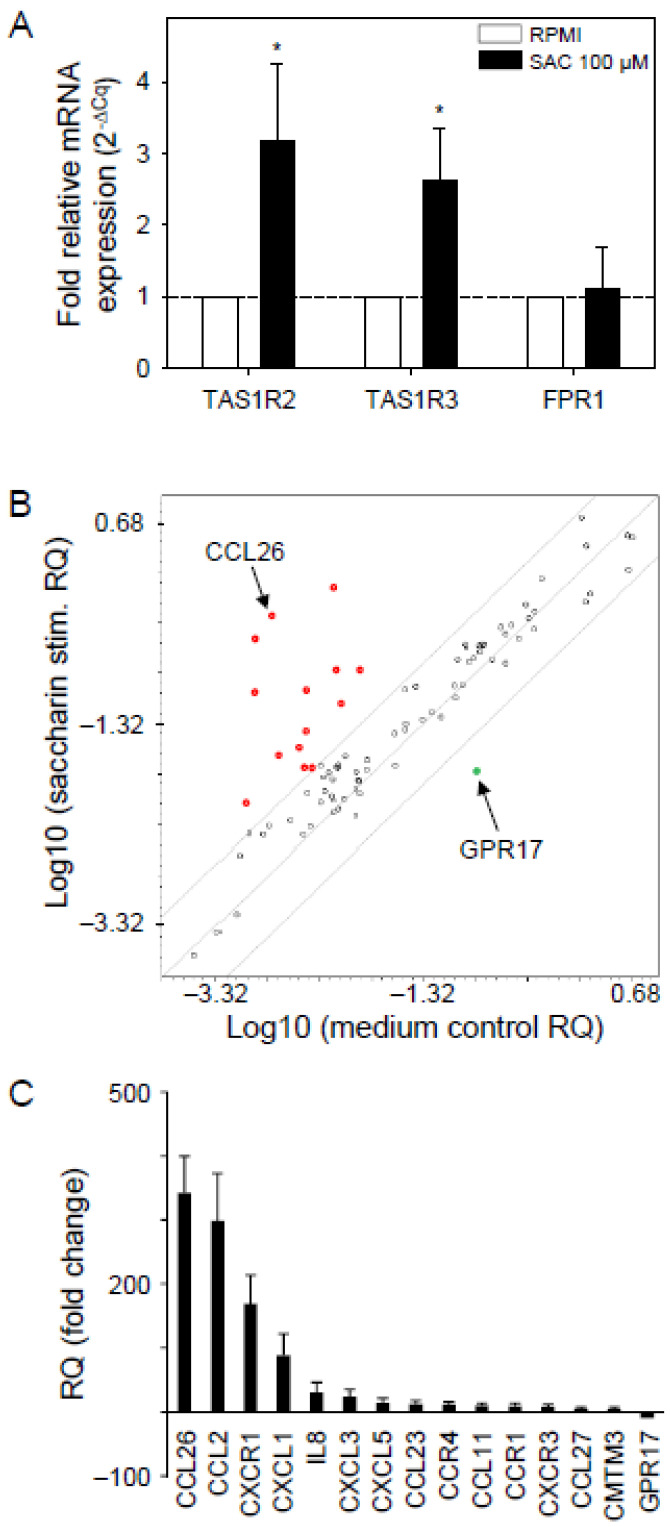
Transcriptional regulation by saccharin of the sweet taste receptor, cytokines, and their cognate receptors in isolated neutrophils in vitro. Isolated neutrophils from buffy coat samples were incubated with saccharin (100 µM) in comparison to RPMI medium for 24 h. (**A**) Transcript levels of TAS1R2, TAS1R3, and FPR1 were obtained from RT-qPCR and are given as 2^−∆Cq^, normalized to each respective standard condition (RPMI) (dashed line). Data are shown as mean ± SEM (*n* = 7 buffy coat samples). Differences in expression levels were tested versus each RPMI condition by paired *t*-test (* *p* < 0.05, two-tailed). (**B**) Scatter plot compares normalized mRNA expression levels between saccharin- and medium-treated samples of 84 genes coding for neutrophil-related cytokines and their receptors (Table S1). Every gene-specific normalized expression level in one group is plotted on one axis against the corresponding value in the other group on the other axis on a log base 10 scale to visualize gene expression changes. The mean fold change in relative quantification (RQ) normalized to medium control (*n* = 3) is shown. Fold changes in gene expression between lines are not significant (black circles, *p* > 0.05). Up-regulated mRNA (red circles) was observed for 14 cytokines and receptors, the highest being CCL26. Down-regulation (green circle) was detected for GPR17. (**C**) Ranked expression of significantly regulated genes in (**B**) is plotted as mean fold change. Data are shown as mean ± SEM.

In peripheral blood monocytes (PBMCs), the regulation of GPCR gene expression is well described [65]. In these cells, for instance, transcript levels of TAS1R3 have been associated with children’s sugary and fatty food consumption [66]. Moreover, a study in human neutrophils of healthy individuals identified a TAS1R locus with significant, inter-individual, epigenetic variations [67,68]. Taste receptors show broad substrate specificity, with saccharin being a typical agonist of the heterodimer of the sweet taste receptor heterodimers TAS1R2 and TAS1R3 in micromolar concentrations [69,70,71,72], as well as for the bitter-taste receptors TAS2R31 and TAS2R43 [46]. Moreover, saccharin activated migration of primary neutrophils in a concentration-dependent manner in chemotactic transmigration assays with an *EC*_50_ of ca. 10 µM [14]. We, therefore, first tested in vitro whether sweet taste receptor subunits’ transcript levels are affected in isolated human neutrophils when challenging them with saccharin in vitro. Incubation for 24 h with saccharin at a concentration of 100 µM resulted in a 2-3-fold, significant upregulation of mRNA for both sweet taste receptor subunits TAS1R2 and TAS1R3 (Figure 2A). In contrast, RNA expression of a non-chemosensory, immune-relevant GPCR, FPR1, which responds to nanomolar concentrations of the chemotactic peptide *N*-Formyl-Methionyl-Leucyl-Phenylalanine (fMLF) [73], was not affected by saccharin (Figure 2A). 

### 3.2. Saccharin Upregulated Gene Expression of Neutrophil Chemokines and Their Receptors in Isolated Neutrophils In Vitro

As a proof of principle, we then investigated whether challenging neutrophils with 100 µM saccharin for 24 h altered the transcription levels of 84 genes (Table S1) coding for neutrophil-related cytokines and their receptors, as well as for other markers involved in immune cell activation and transcription factor regulation (Figure 2B,C). We observed significant upregulation of 14 transcripts (Figure 2B). Among those, three chemokines, CCL26 (342.5 ± 56.9), CCL2 (298.1 ± 74.6), CXCL1 (88.0 ± 33.6), which are chemoattractants for a variety of leukocytes, including neutrophils, as well as receptor CXCR1 (168.5 ± 44.8), showed the highest fold-change in transcript levels (Figure 2C). Notably, the gene transcription of two neutrophil chemokine receptor/ligand pairs, CXCR1/IL8 (CXCL8) and CCR4/CCL2, was significantly upregulated (Figure 2C).

### 3.3. Bio-Appearance of Sweeteners in Healthy Volunteers

**Figure 3:**
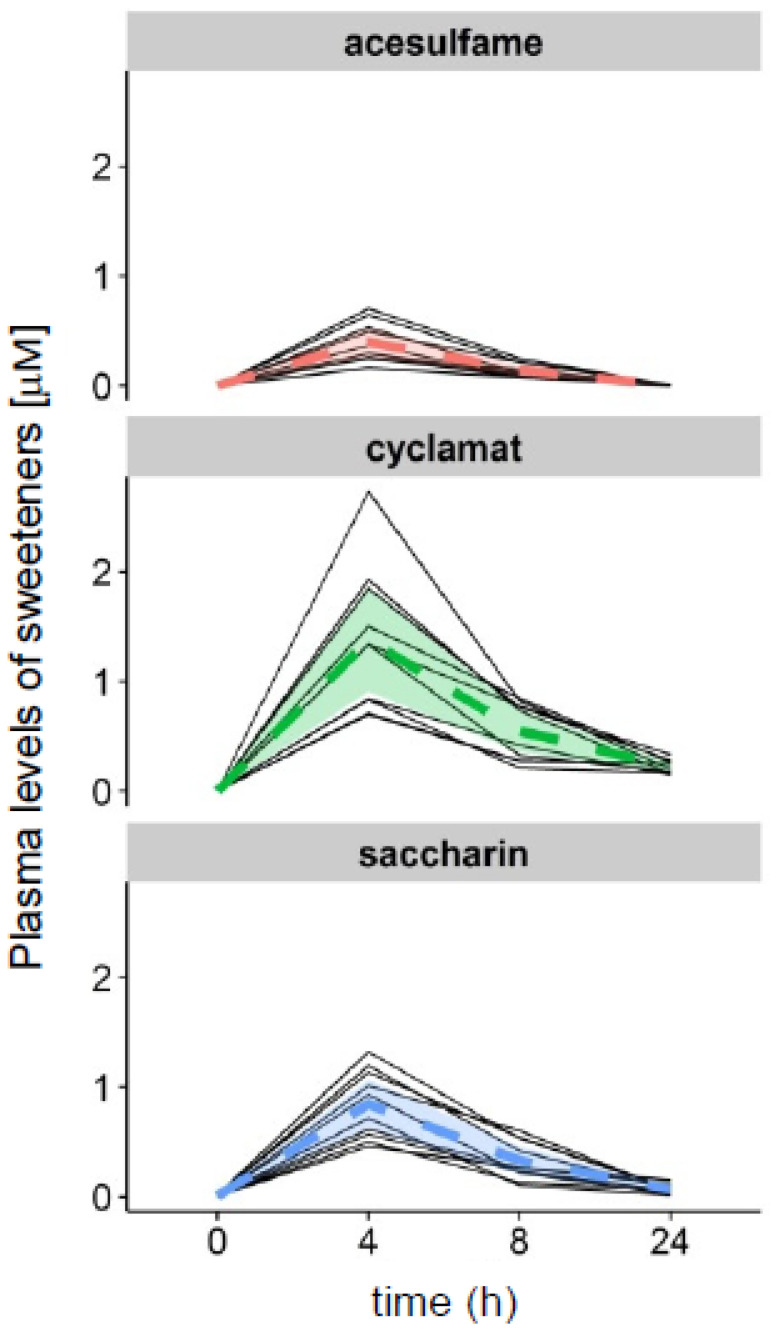
Intervention-derived plasma kinetics of sweeteners in healthy volunteers. Ten volunteers consumed a surrogate beverage containing soft drink-typical concentrations of acesulfame-K, cyclamate, and saccharin dissolved in mineral water (compare Figure 1, Table 1). The appearance of the sweeteners in the plasma was monitored by HPLC-MS/MS over 24 h, as indicated by the time intervals on the *X*-axis. Black lines, individual plasma concentrations of sweeteners; colored dashed line, mean (*n* = 10); colored areas, 95% confidence intervals.

Determining bio-appearance and typical plasma levels of bioactive food compounds is essential for assessing their bio-activity. We, therefore, tested a surrogate beverage, which consisted of diet lemonade-typical concentrations of acesulfame-K (0.27 mM), Na cyclamate (1.13 mM), and saccharin (0.37 mM) in water (Supplemental Material), in a cohort of 10 healthy, adult volunteers, and measured plasma levels of sweeteners at 0 h, 4 h, 8 h, and 24 h (Figure 1 and Figure 3, Table 1). While many non-metabolizable NNS reach peak plasma concentrations already after 0.5 h [23], we chose longer time intervals, since we were interested in a transcriptional regulation, which typically occurs delayed after dietary intervention. In the present study, we observed the highest levels of all compounds 4 h after ingestion, which declined at 8 h until 24 h, where they nearly reached base-line levels again (Figure 3, Table 1 and Table S5).

### 3.4. Plasma-Typical Sweetener Concentrations Increased Transcript Levels of Their Cognate Taste Receptors in Isolated Neutrophils In Vitro

**Figure 4:**
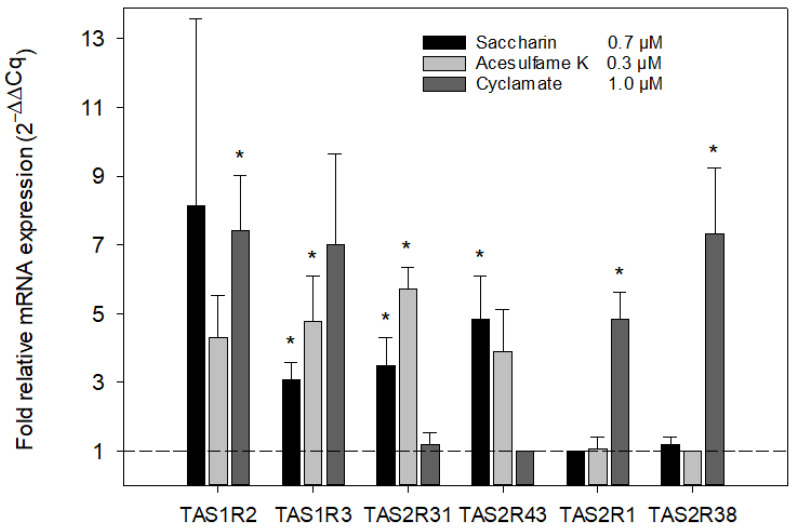
Relative transcript levels for sweet and bitter taste receptors in isolated neutrophils after stimulation with typical plasma concentrations of NNS in vitro. Isolated neutrophils from buffy coat samples were incubated with the different sweeteners at plasma-typical concentrations for 24 h, as indicated. Transcript levels were obtained from RT-qPCR and are given as 2^−∆∆Cq^, normalized to the lowest signals for each sweetener, respectively (dashed line), which always corresponded with their non-cognate receptors: Saccharin–TAS2R1, Acesulfame K–TAS2R38, Cyclamate–TAS2R43. Data are shown as mean ± SEM. Differences in receptor transcript levels were tested for each sweetener versus its respective minimum by paired *t*-test (*n* = six blood samples; * *p* < 0.05, two-tailed).

Beyond activating the sweet taste receptor heterodimer TAS1R2/TAS1R3, sweeteners are known to also trigger certain bitter taste receptors [46,47,71,74]. Saccharin, cyclamate, and acesulfame-K are sweeteners most frequently used in blends with a reduced bitter off-taste, presumably due to the mutual suppression of their respective bitter taste receptor activation [49]. We, therefore, investigated in vitro whether plasma-typical single sweetener concentrations (see Table 1, 4 h post-intervention) may regulate specifically those mRNA levels of their cognate sweet and bitter taste receptors in isolated neutrophils in vitro. After a 24 h incubation of isolated neutrophils with sweeteners, these cells showed significantly increased transcript levels of about 3- to 8-fold of at least one of the sweet taste receptor subunits (Figure 4, Table S6). Notably, all sweeteners upregulated transcript levels exclusively of their respective bitter taste receptors but not of non-cognate receptors. For example, saccharin and cyclamate significantly upregulated transcript levels 4- to 8-fold of TAS2R31, TAS2R43 and TAS2R1, TAS2R38, respectively, but not vice versa (Figure 4).

### 3.5. Beverage-Typical Sweetener Mix Intervention Regulated Transcript Levels of Cognate Taste Receptors in Isolated Neutrophils Ex Vivo

**Figure 5:**
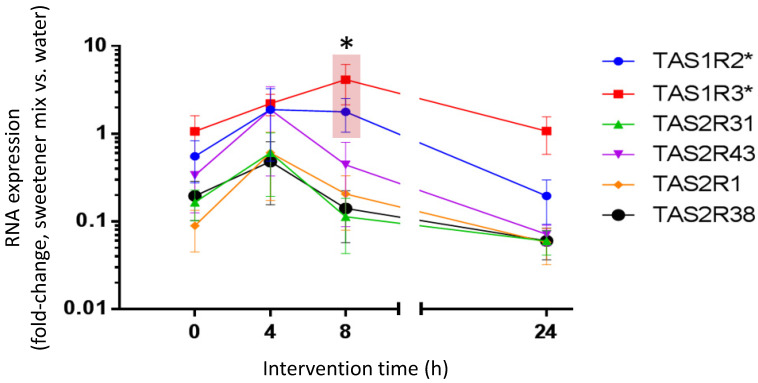
Taste receptor transcript level kinetics during beverage-typical sweetener mix intervention. The RNA was isolated from primary neutrophils of study participants (*n* = 10) before ingestion (0 h) of a food-typical mix of acesulfame-K, cyclamate, and saccharin, as well as 4, 8, and 24 h after ingestion. This was contrasted with matched measurements derived from participants ingesting only water (control). Gene expression was determined by RT-qPCR, compared to the mean expression of three housekeeper genes (2^−ΔCq^), and displayed as fold-change between water control and mix. * TAS1R2 and TAS1R3 expression were significant at 8 h (Wilcoxon sign-rank test; TAS1R2: 8 h vs. 24 h, *p* = 0.0059; TAS1R3: 8 h vs. 0 h, *p* = 0.0195). Data are mean ± SEM.

The post-intervention determined transcript levels of sweetener-cognate taste receptors in neutrophils ex vivo (Figure 5) basically followed the post-intervention plasma level kinetics (Figure 3). However, a significant regulation could only be determined for transcripts of the sweet taste receptor subunits, TAS1R2 and TAS1R3, at 8 h post-intervention (Figure 5). Notably, this increase in transcript levels was fully reversible 24 h post-intervention.

### 3.6. OPLS-DA Score Plots Reveal Reversible Differences between Water and Sweetener Intervention Groups over Time

**Figure 6:**
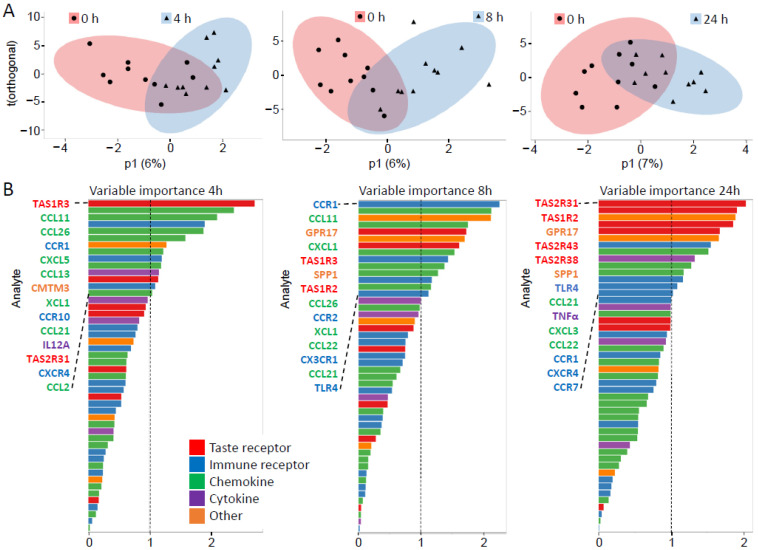
The OPLS-DA and VIP score plots of transcriptional data between sweetener mix and water in healthy volunteers. (**A**) Group differences at increasing post-prandial times are illustrated with OPLS-DA score plots. The figure illustrates the effect of the different intervention times after consumption of the sweetener mix (filled red circles) vs. pre-intervention (filled blue triangles). Each point matches a single volunteer. Multivariate differences between groups are modeled to the effect of treatment, while variation unrelated to the treatment is modeled on the orthogonal component (*y*-axis; t(orthogonal)) and represents primarily individual differences. Between-group differences related to the intervention are modeled on the predictive component (*x*-axis; p1). The ellipses around the grouped individuals give the 95% CI of the multivariate t-distribution of all features in the model. These calculations are based on changes over time in the RT-qPCR-derived transcriptional profile of isolated PMNs after intake of the sweetener surrogate beverage (Figure S2). (**B**) Variable importance in projection (VIP) plots of transcriptional data. The plots rank the 48 analyzed taste receptors, cytokines/chemokines, and their receptors, according to the variable importance scores at the time points 4, 8, and 24 h (post-intervention vs. 0 h). This analysis depicts the relevance of each analyte for the separation in the respective OPLS-DA-plot. Variables above the value of 1 are considered as contributing.

An objection of RT-qPCR-derived transcript levels to a multivariate analysis by OPLS-DA showed a clear separation of the intervention effects already 4 h after sweetener ingestion. However, this group difference became most prominent after 8 h (Figure 6 and Figure S2). Strikingly, although weaker, the intervention effect with the sweetener mixture was still detectable after 24 h (Figure 6, Figures S2 and S3), despite standardized nutrition throughout the study course.

### 3.7. VIP-Plots of Transcripts Indicate Post-Intervention-Specific and Functionally Diverse Gene Sets as Most Relevant Discriminants between Intervention Times

The VIP (variable importance in projection)-plots of transcripts for taste receptors and immune factors (Figure 6B) illustrate the individual contribution of each analyte to the separation in the OPLS-DA analysis (Figure 6A) at the respective times after sweetener intake, and unambiguously demonstrate their varying significance for the OPLS-DA score plot. Overall, it appeared that early on (4 h), transcript levels of mainly chemokines have the highest discriminating power, whereas at 24 h post-intervention the transcript levels of mainly taste and chemokine receptors, as well as some pro-inflammatory and neutrophil priming-related chemokines and receptors, such as TNF-α, CCL22, and TLR4 [75,76,77], have the strongest influence on the separation of intervention times (Figure 6B and Figure S3). GPR17, a receptor evolutionarily related to chemokine receptors [78], also displayed the highest discriminating power at 8 h and 24 h post-intervention. One exception was transcript levels for taste receptor subunit TAS1R3, which showed the highest discriminating power at 4 h, but this influence was lost at 24 h (Figure 6B and Figure S3). At this time, however, most of the other taste receptor transcript levels still contributed substantially to the differences observed between the intervention arms (Figure 6B). Another exception was CCR1, which was an important variable for the differentiation between all sweetener intervention time points and 0 h control.

CCL11 and CCL26 are chemokines with chemotactic activity for the recruitment of a variety of leukocytes [79,80,81]. In the present intervention study, both chemokines, as well as some of their receptors, CCR1, CCR2, and CX3CR1, appeared to be early determinants (4 h, 8 h) for the separation of the intervention times vs. 0 h, with CCR1 being an important variable even at 24 h post-intervention (Figure 6B and Figure S3). CCR7 and one of its ligands, CCL21, have diverse migratory functions in adaptive immunity [65]. CCL21 appeared to be an important variable for the separation of water control and sweetener intervention at all post-intervention times investigated. Interestingly, its receptor CCR7 became an important variable for the differentiation of 24 h post-treatment and 0 h control (Figure 6B and Figure S3).

SPP1, a cytokine that stimulates cytokine production and leukocyte recruitment to inflammatory sites [82], as well as TLR4, which mediates the production of inflammatory cytokines via NF-κB signaling in the innate immune system [83], both were important variables for the differentiation of 8 h or 24 h post-intervention times and 0 h control (Figure 6B and Figure S3). Also, TNF-α, which is a key cytokine in the inflammatory responses of leukocytes [75], became important for the differentiation only between 24 h post-treatment and 0 h control (Figure 6B and Figure S3).

### 3.8. Network and Cluster Analyses Reveal That the Most Relevant Discriminants between Intervention Times Associate with Specific Functional Gene Ontology Networks

**Figure 7:**
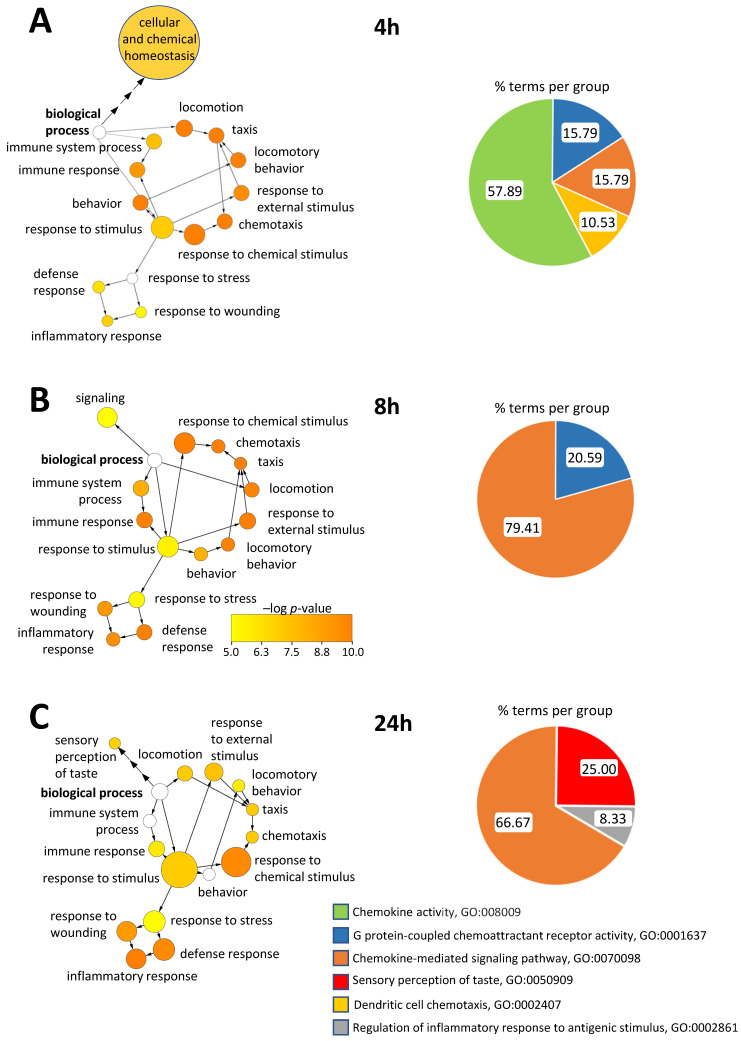
Gene ontology term network analysis of sweetener intervention-regulated VIP-genes. ((**A**–**C**), left panels), functional networks from a BiNGO analysis visualize the VIP-gene sets (Figure 6, Table S7) associated with their significantly overrepresented GO terms at the respective post-intervention times. ((**A**–**C**), right panels), pie-charts from a ClueGO GO term cluster analysis display the percentage of the number of subcategory ontology terms associated with the VIP-genes (Figure 6, Table S7) at the respective post-intervention times.

To investigate whether the intervention time-specific VIP-gene lists (see Figure 6, Table S7) and their associated and overrepresented GO terms underly different functional clusters, we performed a functional network and cluster analysis. The functional network analysis (BiNGO), indeed, revealed striking differences across post-intervention times (Figure 7A–C, left panels). Exclusively at 4 h post-intervention, a homeostasis-related network of significantly overrepresented GO terms became apparent (Figure 7A and Figure S4, left panel), which is absent at later post-intervention times (Figure 7B,C, left panels). Moreover, a cluster analysis (ClueGO) at 4 h post-intervention revealed chemokine activity as the category comprising the majority of significantly overrepresented GO terms (Figure 7A, right panel). At 8 h post-intervention, the homeostasis network is replaced by the category signaling (Figure 7B, left panel). In line with this, the cluster analysis revealed that about 80% of significantly overrepresented GO terms associate with the chemokine-mediated signaling pathway (Figure 7B, right panel). At 24 h post-intervention, the GO term categories response to chemical/stimulus and inflammatory response became more apparent, and chemotaxis- and locomotory behavior-related GO term categories showed lower significance levels as compared to previous intervention times (Figure 7C, left panel). Indeed, the number of genes underlying the GO term category inflammatory response increased with post-intervention time (Table S8), as reflected by the VIP value-corrected node size (Figure 7C, left panel, Table S9). Cluster analysis at 24 h post-intervention revealed that the percentage of, for instance, significantly overrepresented GO terms under the chemokine-mediated signaling pathway decreased from 79% at 8 h to 67%, whereas 25% of GO terms newly associated with sensory perception of taste and 8% newly associated with regulation of inflammatory response to antigenic stimulus (Figure 7C, right panel, Table S10). A GO term functional network analysis of all 48 genes investigated comprised all VIP gene-related sub-networks predominant at the different sweetener intervention times, albeit at different significance levels (Figure S5). For example, the GO term category inflammatory response was more apparent in the functional network analysis of all 48 genes investigated (Table S8) at orders of magnitude higher significance levels (Figure S5, color-coded scale), as compared to each sweetener intervention time (Figure 7, left panels). The percentage of significantly overrepresented GO terms of the VIP gene sets at each respective intervention time (Figure 7, right panels), however, was largely increased, compared to a GO term cluster analysis with all 48 genes analyzed (Figure S6, Table S11).

### 3.9. Sweetener Mix-Induced Ca^2+^ Influx Increased Neutrophils’ Sensitivity for fMLF

Intracellular Ca^2+^ signaling is involved in most neutrophils’ cellular immune responses [84,85] and in neutrophil priming [86,87]. Intracellular Ca^2+^ in neutrophils typically is modulated via GPCRs and Ca^2+^-store- or ligand-operated Ca^2+^-channels, resulting in Ca^2+^-release from intracellular stores and/or Ca^2+^-influx over the plasma membrane [84,85,88,89,90,91,92]. It has long been known that many priming agents induce transient Ca^2+^ signals and enhance an activation-induced Ca^2+^-influx into neutrophils [86,87]. Since our intervention study suggested that NNS may shift the transcriptional profile of neutrophils from ‘homeostasis’ to ‘priming’, we thus set out to investigate whether NNS interfere with the Ca^2+^ homeostasis in neutrophils isolated from commercially available buffy coat samples.

**Figure 8:**
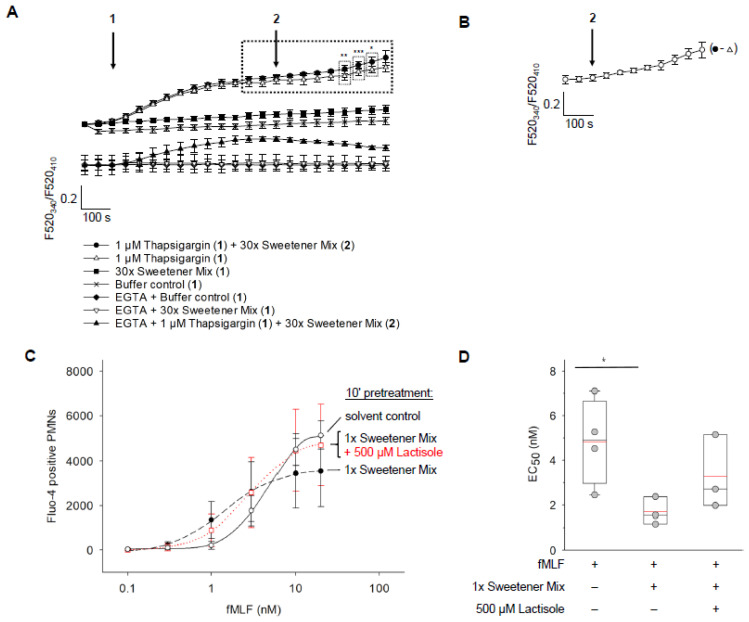
Sweetener-induced facilitation of fMLF-stimulated Ca^2+^ signaling in isolated PMNs. (**A**) Sweetener-induced priming effects of freshly isolated PMNs stimulated with 30× sweetener mix. Fluorescence was measured by a spectrofluorometer with dual excitation at 340 nm and 410 nm, and emission at 520 nm. Arrows indicate application times (“1”: 2 min; “2”: 10 min). The fluorescence ratio F520340/F520410 was calculated and normalized to the initial ratio at 0 s. Data points represent mean ± SD (*n* = 3). * *p* = 0.032; ** *p* = 0.008; *** *p* < 0.001 (paired, two-tailed Student’s *t*-test). (**B**) Shown is a 30× sweetener-induced increase in Ca^2+^ influx as kinetic of ratio F520340/F520410 in the presence of 1 µM thapsigargin and 30× sweetener mix after subtraction of ratio F520340/F520410 in the presence of 1 µM thapsigargin only from (**A**). Data are mean ± SD (*n* = 3). (**C**) fMLF-induced concentration-response relations of pre-stimulated PMNs under varying conditions. Cells were pre-stimulated with 1× sweetener mix or solvent control for 10 min followed by the addition of fMLF at concentrations ranging from 0.1 nM to 20 nM. To test for an inhibition of a TAS1R2/TAS1R3-mediated effect of the 1× sweetener mix on the fMLF concentration–response relation, lactisole (500 µM) was added to the sweetener mix during pre-stimulation of cells. Sweeteners and lactisole were present during fMLF stimulation. Fluorescence was measured until 10,000 events were obtained by fluorescence-activated cell analysis using 494 nm excitation and 506 nm emission. A total of 0.1% DMSO was used as solvent control and subtracted from each measurement, using 5% to gate positive signals. Data are mean ± SD (*n* = 3, *n* = 4 for fMLF). (**D**) *EC*_50_ values derived from (**B**) are displayed as box plots with medians (black horizontal lines) and calculated means (red horizontal lines); * *p* = 0.045 (two-tailed Student’s *t*-test).

The 30× sweetener mix, including saccharin, acesulfame-K, and cyclamate, neither induced a detectable, transient Ca^2+^ release from intracellular stores, nor a Ca^2+^ influx signal in spectrofluorimetric experiments with Fura-8-loaded neutrophils (Figure 8A). However, under ‘activation’ conditions where Ca^2+^ stores had been emptied (Thapsigargin), the 30× sweetener mix induced a long-lasting, small, albeit significant Ca^2+^ influx. This Ca^2+^ influx signal was abolished under conditions omitting extracellular Ca^2+^ (EGTA) (Figure 8A,B).

We next investigated whether NNS at post-prandial plasma concentrations are capable of modulating Ca^2+^ signaling in neutrophils, which has been activated by the application of the damage- or pathogen-associated formylpeptide fMLF (*N*-formyl-Met-Leu-Phe), a prototypical high-affinity ligand for the formyl peptide receptor FPR1 [93,94,95]. The leukocyte attractant fMLF regulates innate immunity and host defense via several FPR-mediated signaling pathways, including Ca^2+^ signaling [88,89,96,97]. Thus, we tested whether a diet lemonade-typical NNS mix, comprising saccharin, acesulfame-K, and cyclamate at concentrations identified post-prandially in human plasma (1× sweetener mix, see Figure 3, Table 1), can modulate fMLF-induced Ca^2+^ signaling in isolated human neutrophils. Surprisingly, a 10 min pretreatment of isolated neutrophils with the 1× sweetener mix shifted the fMLF concentration–response curve to a significantly lower *EC*_50_. This effect was only partially reverted by lactisole, an inhibitor of the sweet taste receptor [98] (Figure 8C,D), suggesting a potential involvement of additional receptors in neutrophils as targets of NNS, e.g., bitter taste receptors [14,46,49], or the TRPV1 receptor [99,100].

## 4. Discussion

Artificial high-intensity sweeteners are widely consumed due to their reduced energy content and low glycemic effect, which is relevant especially for bw control and under hyperglycemic conditions. While their use in food has been controversially discussed for decades concerning their potentially negative impact on metabolism [18,20,21,24,31,36], they are however considered safe for the general population under certain conditions [39,41,42,43]. 

Beyond our canonical chemical sense of taste, the expression of the sweet-taste receptor on, e.g., intestinal cells [101,102] or blood immune cells [14] suggested their modulation by the typical post-prandial plasma concentration of artificial sweeteners. Moreover, sweeteners have been demonstrated to selectively target their cognate bitter taste receptors [46,47,49,74] with extra-oral functions in, e.g., the respiratory tract [13,103], gastrointestinal system [104], or the immune system [14,51]. Previously, we have shown in a siRNA-controlled study that saccharin was capable of triggering sweet taste receptor-dependent chemotactic migration in isolated neutrophils in vitro [14].

Beyond sweet and bitter taste receptors, sweeteners may activate further biological targets. For instance, it has been suggested that saccharin activates chemesthesis-related TRPV1 channels [100], albeit at rather high (mM) concentrations. Neutrophils do express TRP channels, including the TRPV1 channel [99,105,106,107,108]; however, in our intervention study, we did not investigate TRP transcript levels.

In the present study, we show that plasma-typical concentrations of sweeteners upregulated transcript levels of their cognate taste receptors in vitro and ex vivo. Whereas mainly cytokines emerged as significant early discriminants in the present sweetener intervention study, at 24 h post-intervention, mainly taste and immune-related receptors appeared to have the highest discriminating power between pre-intervention and sweetener intervention. These observations were corroborated by our cluster analysis. The significant contributors that we identified to distinguish fasting state from sweetener intake ex vivo across all post-prandial time points overlapped with 60% of the transcripts that we found to be significantly regulated by saccharin 24 h in vitro. Comparing just the 24 h sweetener mixture intervention in vivo with the 24 h saccharin challenge in vitro, three significantly regulated transcripts emerged under both conditions, among them GPR17 and CCR1. Chemokine GPCR-related receptor GPR17 has been suggested to regulate inflammatory immune responses [109]. Indeed, inflammation/stress-related significantly overrepresented GO terms became more prominent 24 h post-intervention, according to our BiNGO analysis. During the in vivo challenge with the sweetener mixture, CCR1 emerged as an important variable for the differentiation between 0 h control and sweetener intervention at all post-prandial time points, as did CXCR4, at least at 4 h and 24 h post-intervention.

The activation status of neutrophils shows high flexibility from resting state over primed to fully activated [87]. In light of the context that (i) CCR1 and CXCR4 transcripts are regulated in neutrophils when exposed to inflammatory environments or pro-inflammatory, neutrophil priming-related cytokines [110,111,112], and that (ii) ubiquitin is an endogenous ligand of CXCR4 and an anti-inflammatory immune modulator [113], we might speculate that one late post-prandial effect of saccharin is to shift neutrophils to a primed state with inflammatory alertness. This is in line with our observation that at 24 h post-intervention, inflammation-related immune modulators, such as TLR4 and TNF-α, were major discriminants. TNF-α, which is frequently used as a model priming agent, is a key cytokine in the inflammatory responses of leukocytes [114]. A hallmark of primed neutrophils is delayed apoptosis, which could be at least partially mediated via TLR4, a principal regulator of neutrophil survival [115]. Increased inflammatory alertness is corroborated by our network analysis, showing that the significantly overrepresented GO term ‘inflammatory response’ became most prominent at 24 h post-intervention.

Chemokines and their GPCRs, such as CCL21 and CCR7, are not only important mediators of innate and adaptive immune responses in acute inflammation or infection, but also are continuously fine-tuning an immunological homeostasis, balancing immunity and tolerance [65,77,116,117,118]. In the present study, CCL21 was an important discriminant at all post-intervention time points, its receptor CCR7 at least at 24 h post-intervention. SPP1, which in the present study was a very important variable for the differentiation between 0 h control and 8 h or 24 h post-intervention, has been suggested to have homeostatic immune functions [119], but also has been shown to be involved in inflammation [82]. Since in our study significantly overrepresented, homeostasis-associated GO terms were absent at 8 h and 24 h post-intervention, but inflammation/stress-related GO terms gained prominence at 8 h and 24 h post-intervention, a function of SPP1 might therefore be associated with an inflammation-related neutrophil function.

Care must be taken, though, to not interpret the results from our sweetener intervention study solely towards inflammation-related processes. Indeed, the GO term inflammatory response was overrepresented in the functional network analysis of all 48 genes investigated, and at much higher significance levels as compared to the network analysis of the VIP gene sets, which were relevant for separating experimental groups at any sweetener post-intervention time from 0 h control. This, however, may reflect the lack of eminent inflammation- and neutrophil activation-related genes, such as IL-8 (CXCL8) and its receptors, CXCR1 and CXCR2, in all VIP gene sets, suggesting that a full-blown inflammatory immune response would hardly be triggered by the consumption of food-typical sweetener concentrations. Notably, a recent study reported that an 8-week intervention with diet soda sweetened with sucralose and acesulfame K altered the inflammatory transcriptome of adipose tissue from overweight females, but, however, did not alter circulating inflammatory markers [120].

Importantly, though, inflammation-related cytokines, such as TNFα, may have modulatory functions on, e.g., taste receptor responses [121], suggesting an intimate relationship between a chemosensory and/or chemoreceptive internal detection of food ingredients and an immune function. Knowing that restricting our study to a selection of immune mediators might confer a potential risk of bias, however, our study clearly demonstrates a biological principle that specific food ingredients do interfere with the transcriptional profile in neutrophils.

Interestingly, in our hands the 30× sweetener mix induced a detectable Ca^2+^ influx only into activation-simulated neutrophils with thapsigargin-depleted intracellular Ca^2+^ stores displaying elevated cytosolic Ca^2+^ levels. Vice versa, a fMLF-induced Ca^2+^ signaling was facilitated in the presence of 1× sweetener mix, with the fMLF concentration–response curve shifted to lower concentrations. This suggests crosstalk between NNS- and fMLF-activated receptors and their signaling pathways in neutrophils, with a synergistic effect, at least at the level of Ca^2+^ signaling. G protein-coupled receptor crosstalk, heterologous sensitization, and the facilitation of fMLF/FPR1-induced Ca^2+^ signaling are well-known principles in neutrophils [99,122,123]. Since an elevated Ca^2+^ level in neutrophils is a prerequisite for most of neutrophils’ cellular functions, thus, the simultaneous presence of NNS and pathogen- or damage-associated molecular patterns (PAMPs or DAMPs) may lead to the more sensitive and efficient cellular immune responses of neutrophils. A recent study corroborates our results: Mol et al. (2021) demonstrated that a full activation of neutrophils required the simultaneous presence of adequate stimuli for at least two different receptor systems [124]. The minor, non-significant effect of lactisole in the present study may point to the involvement of, e.g., lactisole-insensitive but sweetener-specific TAS2R bitter taste receptors [14,46,49], which may have participated additionally to the sweet taste receptor in mediating the significant shift of the fMLF concentration-Ca^2+^ response relation to lower concentrations after priming with sweetener mix.

## 5. Conclusions

In summary, we show that post-prandial, plasma-typical concentrations of artificial sweeteners altered a resting state transcriptional profile of their cognate taste receptors, as well as factors typically orchestrating innate immunity in peripheral blood leukocytes towards a more priming-related status, involving factors of pro-inflammatory signaling. Their differential transcriptional kinetics suggest cytokines or taste and immune receptors as early or late discriminants, respectively, between the pre-intervention control and sweetener intervention groups.

We further show that beyond altering the transcriptional profile of sweetener-cognate taste receptors and immune factors, NNS at post-prandial plasma concentrations indeed facilitated an fMLF-induced cellular Ca^2+^ signaling response in isolated neutrophils.

We, therefore, hypothesize that taste receptors of immune cells are sensors for food-borne stimuli, enabling a post-prandial, flexible, and reversible alertness of our immune system.

## Figures and Tables

**Table 1:** Plasma concentrations of sweeteners in healthy volunteers at different intervention times.

Time (h)	Saccharin	Acesulfame-K	Cyclamate
	Plasma Concentration (µM)		
0	0.01 ± 0.01	<0.01	<0.01
4	0.85 ± 0.29	0.39 ± 0.18	1.38 ± 0.62
8	0.33 ± 0.17	0.14 ± 0.06	0.55 ± 0.25
24	0.07 ± 0.04	<0.01	0.22 ± 0.06

Data are mean ± SD of *n* = 10 individual samples.

## Data Availability

The human intervention study was conducted in accordance with the Declaration of Helsinki, and approved by the ethical commission of the faculty of medicine at the Technical University of Munich, Germany (#5798/13, approved on 27 June 2013)). Clinical Trial Registry number and website: This study was registered in the German Clinical Trials Register (WHO registered) (DRKS; #DRKS00005083, accessed on 1 March 2023). Written informed consent has been obtained from all subjects involved in the study. Data described in the manuscript, code book, and analytic code will be made available upon request pending application.

## Supplementary Materials

The following supporting information can be downloaded at: https://www.mdpi.com/article/10.3390/nu15051260/s1, Figure S1: Matrix-matched calibration curves. A, analyte. IS, internal standard; Figure S2: Post-intervention transcript level kinetics of 48 immunity- and chemoreception-relevant genes; Figure S3: VIP plot of transcriptional data between sweetener mix and water 0 vs. 24 h; Figure S4: Bingo (v3.0.3) analysis in Cytoscape (v3.7.1) of only the VIP genes relevant for the separation in the OPLS-DA analysis of the 4 h sweetener mix intervention group versus the 0h control group (Figure 4A); Figure S5: Bingo (v3.0.3) analysis in Cytoscape (v3.7.1) of all 48 genes (Table S4) in the sweetener mix intervention study; Figure S6: Pie-charts from a ClueGO GO term cluster analysis display the percentage of the number of subcategory ontology terms associated to all 48 genes analyzed in the sweetener mix intervention study; Table S1: List of 84 cytokines and receptors for which saccharin-dependent transcript levels (RQ values) have been determined by RT-qPCR in isolated neutrophils in vitro; Table S2: All 48 genes investigated during sweetener mix intervention; Table S3: Chromatographic and spectrometric properties of the analytes and the internal standard, linear range and properties of the calibration curves established in blank plasma; Table S4: Accuracy and precision values of the method, determined in analyte-free plasma; Table S5: Intervention-derived plasma kinetics of sweeteners in healthy volunteers (data to Figure 3); Table S6: Taste receptor transcript level kinetics during beverage-typical sweetener mix intervention (data to Figure 4); Table S7: VIP-gene lists and accession numbers at 4 h, 8 h, and 24 h post-intervention; Table S8: BiNGO results for GO-term‚ inflammatory response; Table S9: BiNGO results, *p*-values, and VIP-normalized node-sizes; Table S10a: ClueGO results_4 h sweetener mix intervention; Table S10b: ClueGO results_8 h sweetener mix intervention; Table S10c: ClueGO results_24 h sweetener mix intervention; Table S11: ClueGO results_all 48 genes.

## Abbreviations

bw, body weight; GPCR, G protein-coupled receptors; PMNs, polymorphonuclear neutrophils; NNS, non-nutritive sweetener; MIQE, minimum information for publication of quantitative real-time PCR experiments; CCL, C-C motif chemokine ligand; CCR, C-C motif chemokine receptor; CI, confidence interval; CXCL, C-X-C motif chemokine ligand; CXCR, C-X-C motif chemokine receptor; CX3CR1, C-X3-C motif chemokine receptor type 1; GM-CSF (Granulocyte-macrophage colony stimulating factor); OPLS-DA, orthogonal projections to latent structures-discriminant analysis; SPP1, secreted phosphoprotein 1; TAS1R2, TAS1R3, sweet taste receptor subunits; TAS2R(x), bitter taste receptors, TLR4, toll-like receptor type 4; TRP channels, transient receptor potential channels; TRPV1, transient receptor potential cation channel subfamily V member 1 (vanilloid receptor).
