# Supplementary material for: Sweetener System Intervention Shifted Neutrophils from Homeostasis to Priming
Source: Nutrients. 2023 Mar 2;15(5):1260. doi: 10.3390/nu15051260 (PMC10005247; doi:10.3390/nu15051260)
Supplement: Supplementary file 1 [file nutrients-15-01260-s001.zip › nutrients-2218630-supplementary.pdf]

Supplemental Material – Sweetener system intervention shifted a neutrophils transcript profile from homeostasis to priming

Table S1: List of 84 cytokines and receptors for which saccharin-dependent transcript levels (RQ values) have been determined by RT-qPCR in isolated neutrophils in vitro

Cytokine/ receptor	RQ value (mean, *n*=3)	Cytokine/ receptor	RQ value (mean, *n*=3)
CCL26	342.5095	CCL24	1.5298
CCL2	298.1718	VEGFA	1.5157
CXCR1	168.5072	IL5	1.4948
CXCL1	88.0347	IL18	1.4931
IL8	30.204	IL13	1.4726
CXCL3	23.8073	CMTM4	1.4709
CXCL5	14.3204	CMKLR1	1.4456
CCL23	12.2101	CXCR5	1.3044
CCR4	11.5514	CCR7	1.2775
CCL11	9.9177	CCL21	1.2512
CCR1	9.084	IL15	1.2454
CXCR3	8.6139	CCR2	1.1947
CCL27	5.2416	IL2	1.1865
CMTM3	4.4025	IL12B	1.1851
IL10	3.9041	CCL14	1.162
CMTM2	3.7408	IL16	1.1554
CXCL9	3.2716	IL17F	1.1342
CCL5	3.1969	IL1B	1.093
IL3	3.0105	CXCR6	1.0918
CCRL2	2.9828	CCR10	1.0595
IL9	2.8415	TNF	1.0546
CCR5	2.7766	IL1A	1.0401
CCBP2	2.7101	IL4	1.014
CKLF	2.6945	IFNG	1.007
FPR1	2.682	CXCR2	–1.0257
CCL13	2.6697	IL27	–1.0705
CCL16	2.5847	CCL15	–1.0743
CSF3	2.5847	CXCL13	–1.1381
CCL3	2.4061	CCL28	–1.162
CCR3	2.395	CCRL1	–1.1837
CXCL6	2.2191	TNFSF13B	–1.1947
CXCR4	2.1287	RTC	–1.2086
IL17A	2.0898	IL12A	–1.2198
CCL17	2.0232	PPC	–1.2354
CCL4	2.0046	CSF2	–1.2746
LTB	1.9097	IL22	–1.355
CCR8	1.7532	GPI	–1.3851
CD40LG	1.6586	IL1RN	–1.5619
CCR9	1.651	FASLG	–1.8025
SPP1	1.6245	IL6	–2.2763
CCR6	1.5837	CCL25	–2.8024
IL21	1.5801	GPR17	–9.5798

Cytokines or receptors for which transcript levels were significantly regulated upon challenging neutrophils with 100 µM saccharin or RPMI for 24 h.

Table S2: All 48 genes investigated during sweetener mix intervention

ACKR3	CCL11	CCL13	CCL19	CCL2	CCL21
CCL22	CCL23	CCL26	CCL27	CCL7	CCL8
CCR1	CCR10	CCR2	CCR3	CCR4	CCR7
CKLF	CMTM3	CX3CR1	CXCL1	CXCL10	CXCL3
CXCL5	CXCL8	CXCL9	CXCR1	CXCR2	CXCR4
CXCR5	GNAT3	GPR17	IL12A	IL13R	IL4
SPP1	TAS1R2	TAS1R3	TAS2R1	TAS2R31	TAS2R38
TAS2R43	TLR2	TLR4	TNF	TYMP	XCL1

Amount of sweeteners in the test beverage

Na-saccharin 0.37 mM, acesulfame-K 0.27 mM, Na cyclamate 1.13 mM.

Quantification of artificial sweeteners in human plasma

Standard solutions*:* Stock solutions of the analytes (roughly 10 mM) were individually prepared by dissolving the exactly weighed solids of acesulfame K, sodium saccharin and sodium cyclamate in methanol. The internal standard p-toluolsulfonic acid hydrate was prepared in acetonitrile at a concentration of ~10 mM and the exact concentration determined by qNMR versus a caffeine standard as reported recently [1]. The internal standard was diluted with acetonitrile (1+9,999, v+v) to obtain an internal standard working solution with 1 µM.

Matrix-matched calibration*:* For matrix matched calibration, aliquots of the analyte stocks were combined to yield a multicomponent stock and diluted to 100 µM with water. This multicomponent dilution was diluted with water to obtain a total of 8 aqueous solutions (100, 50, 25, 12.5, 5.0, 2.5, 1.25, 0.63 µM). Aliquots of these dilutions (100 µL) were added to aliquots (900 µL) of blank human plasma (purchased from Sigma-Aldrich), as confirmed beforehand by HPLC-MS/MS, yielding spiked plasma samples with final analyte concentrations of 10, 5.0, 2.5, 1.25, 0.50, 0.25, 0.125 and 0.06 µM plasma. These calibration samples were processed and analysed in triplicate as detailed below, to establish calibration curves. Two additional samples each of the 0.125 µM, 0.50 and 2.5 µM spiked plasma were processed for assessment of accuracy and precision.

Quantitative analysis*:* In an Eppendorf cup an aliquot of the plasma sample (100 µL) was mixed with the internal standard working solution (1 mL). The mixture was vortexed and centrifuged (12,500 rpm, 4°C, 15 min). The clear supernatant was decanted into a new Eppendorf cup and evaporated in a stream of nitrogen. The residue was taken up in a mixture of water and acetonitrile (95+5, v/v, 100 µL). Aliquots (1 µL) were injected into the HPLC-MS/MS system.

Instrumentation*:* MS/MS data were acquired on a 3200 triple quadrupole mass spectrometer (ABSciex, Darmstadt, Germany). Potentials applied for introduction of the compounds into the ion source (Q1 mass, declustering potential) and the fragmentation related parameters (cell entrance potential, collision energy, cell exit potential) were optimized by using the autotune-function of the software Analyst 5.1.1 (ABSciex, Darmstadt, Germany). The delay between two mass transitions was adjusted from 5 to 8 ms. Ion source temperature was 450°C, curtain gas (air) 25 psi, heater gas (air) and nebulizer gas (nitrogen) were 50 and 60 psi. Ion spray voltage was set to -4.5 kV.

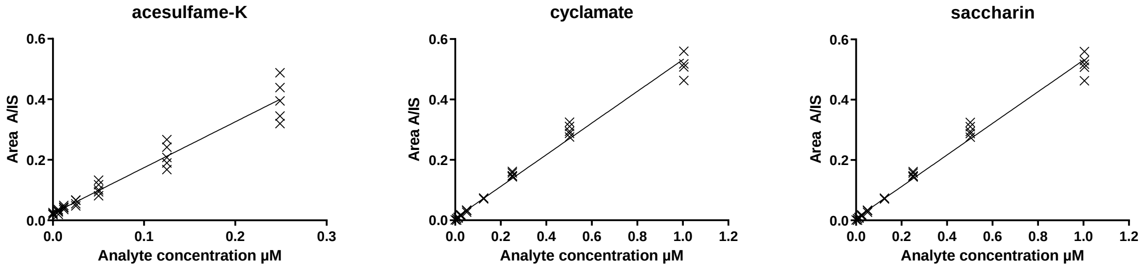

Figure S1: Matrix-matched calibration curves. A, analyte. IS, internal standard.

Table S3: Chromatographic and spectrometric properties of the analytes and the internal standard, linear range and properties of the calibration curves established in blank plasma.

**Compound**	**Q1/Q3 (m/z)**[a]	**Rt. (min)**	**Linear range (µM)**[b]	**Weighing**	$\mathbf{R^2}$
Saccharin	182/42*, 106, 62	6.87	0.06 – 5.0	1/x	0.989
Cyclamate	178/80*, 64, 96	6.64	0.06 – 5.0	1/x	0.981
Acesulfame-K	162/82*, 78, 64	5.74	0.12 – 2.5	1/x	0.974
p-Toluolsulfonate (IS)	171/80*, 107, 64	6.92			

[a] Q1/Q3 transitions marked with "*" were used for quantitation; [b] linear range refers to the concentration in spiked plasma

Table S4: Accuracy and precision values of the method, determined in analyte-free plasma.

	Acesulfame-K	Cyclamate	Saccharin
Nominal (µM)	0.12	0.13	0.13
Found (µM)	0.12	0.13	0.13
Accuracy (%)	92.2	102.7	106.8
Precision (RSD, %)	0.5	3.5	9.5
Nominal (µM)	0.50	0.50	0.50
Found (µM)	0.51	0.59	0.55
Accuracy (%)	101.2	116.8	110.4
Precision (RSD, %)	1.7	5.3	5.5
Nominal (µM)	2.50	2.51	2.51
Found (µM)	2.28	2.92	2.78
Accuracy (%)	91.5	116.4	110.5
Precision (RSD, %)	0.9	0.3	1.1
Data are means of individually processed duplicates each injected in triplicates.			

Table S5: Intervention-derived plasma kinetics of sweeteners in healthy volunteers (data to Fig.3).

Time (h)	Saccharin plasma concentration (µM)										mean (µM)	SD (µM)
0	0.0029	0.0186	0.0103	0.0104	0.0037	0.0042	0.0163	0.0061	0.0230	0.0012	0.010	0.007
4	0.9289	1.1338	0.5009	1.1952	0.7179	0.4590	1.3207	0.5690	0.6129	1.0109	0.845	0.297
8	0.2180	0.5413	0.1297	0.4175	0.1045	0.2645	0.5331	0.2298	0.2630	0.6034	0.330	0.170
24	0.0237	0.0735	0.0570	0.0659	0.0137	0.1522	0.0670	0.0556	0.1174	0.0574	0.068	0.039
Time (h)	Acesulfame-K plasma concentration (µM)										mean (µM)	SD (µM)
0	0.0002	0.0001	0.0001	0.0001	0.0002	0.0045	0.0001	0.0002	0.0001	0.0001	0.001	0.001
4	0.3651	0.6446	0.1611	0.5329	0.2588	0.2502	0.7014	0.2490	0.2922	0.4923	0.395	0.177
8	0.0954	0.2177	0.0762	0.1154	0.0781	0.1474	0.2488	0.1029	0.1122	0.2165	0.141	0.060
24	0.0008	0.0031	0.0009	0.0013	0.0003	0.0077	0.0035	0.0041	0.0039	0.0015	0.003	0.002
Time (h)	Cyclamate plasma concentration (µM)										mean (µM)	SD (µM)
0	0.0002	0.0016	0.0008	0.0010	0.0011	0.0024	0.0033	0.0007	0.0016	0.0026	0.002	0.001
4	1.9285	1.5035	0.8351	2.7293	1.3358	0.7115	1.8480	0.8428	0.6982	1.3391	1.377	0.621
8	0.7349	0.8495	0.2118	0.8256	0.3276	0.2811	0.7683	0.4203	0.2760	0.7767	0.547	0.250
24	0.1589	0.2648	0.1660	0.2001	0.1768	0.2522	0.2824	0.1476	0.2489	0.3355	0.223	0.059

Table S6: Taste receptor transcript level kinetics during beverage-typical sweetener mix intervention (data to Fig.4).

ID	Group	TAS1R 2	TAS1R 3	TAS2R 31	TAS2R 43	TAS2R 1	TAS2R 38
**AD1**	**1**	0.0006	0.0036	0.0362	0.0279	0.0294	0.0184
**AD2**	**2**	0.1085	0.0043	0.0537	0.1358	0.1079	0.0177
**AD3**	**3**	0.0339	0.0189	0.0321	0.0052	0.0867	0.0066
**AD4**	**4**	0.0121	0.0057	0.0817	0.1741	0.1763	0.0359
**BO1**	**1**	0.1180	0.0330	0.0003	0.0736	0.0048	0.0385
**BO2**	**2**	0.0377	0.0832	0.0054	0.0199	0.0121	0.0150
**BO3**	**3**	0.0141	0.0168	0.0608	0.0997	0.0585	0.0628
**BO4**	**4**	0.0128	0.1379	0.0297	0.1223	0.2529	0.1827
**FB1**	**1**	0.0450	0.1495	0.1326	0.0699	0.0233	0.0465
**FB2**	**2**	0.1055	0.4451	0.4160	0.3934	0.0086	0.0194
**FB3**	**3**	0.0565	1.0780	0.0024	0.0320	0.0079	0.0337
**FB4**	**4**	0.0014	0.0832	0.0558	0.0257	0.0057	0.0126
**KG1**	**1**	0.7835	5.2876	0.1273	0.1285	0.0139	0.0275
**KG2**	**2**	0.4448	4.5645	0.0282	0.0179	0.0079	0.0052
**KG3**	**3**	5.0622	14.057	0.0007	0.0066	0.0002	0.0014
**KG4**	**4**	0.3724	4.3337	0.2137	0.0071	0.0274	0.0328
**NR1**	**1**	0.0059	3.3995	0.6159	0.3769	0.0957	0.7221
**NR2**	**2**	0.0049	2.3327	0.1282	0.0690	0.0490	0.1375
**NR3**	**3**	0.3853	18.852	0.0514	0.1699	0.4200	0.2395
**NR4**	**4**	0.0029	2.7716	0.0861	0.0823	0.0430	0.2296
**RS1**	**1**	0.7524	0.1827	0.0685	0.1831	0.0087	0.0930
**RS2**	**2**	1.3951	4.3224	0.4710	0.3893	0.0783	0.2147
**RS3**	**3**	0.3034	0.5208	0.0813	0.0980	0.0083	0.0252
**RS4**	**4**	0.0646	2.9241	0.0538	0.1515	0.0117	0.0313
**SL1**	**1**	0.0151	0.4525	0.0010	0.0071	0.0048	0.0000
**SL2**	**2**	0.0484	2.3406	0.0030	0.0056	0.0067	0.0001
**SL3**	**3**	0.0818	0.5577	0.0061	0.0297	0.0327	0.0019
**SL4**	**4**	0.0009	0.1710	0.0043	0.0043	0.0042	0.0009
**SiKo1**	**1**	0.3895	0.2866	0.2129	0.1593	0.2328	0.1456
**SiKo2**	**2**	15.007	5.2724	0.4540	1.1245	1.1603	0.8823
**SiKo3**	**3**	6.2058	4.5582	0.1059	0.1555	0.0538	0.0617
**SiKo4**	**4**	0.1307	0.1741	0.0004	0.0009	0.0001	0.0001
**SK1**	**1**	3.0431	0.1311	0.0041	0.0212	0.0183	0.0331
**SK2**	**2**	0.5633	0.0045	0.0118	0.0797	0.0441	0.0374

Mean	h	TAS1R 2	TAS1R 3	TAS2R 31	TAS2R 43	TAS2R 1	TAS2R 38
	**0**	0.5530	1.0663	0.1648	0.3344	0.0892	0.1946
	**4**	1.8788	2.2054	0.6065	1.8805	0.6058	0.4803
	**8**	1.7772	4.1436	0.1128	0.4419	0.2054	0.1403
	**24**	0.1950	1.0692	0.0598	0.0713	0.0577	0.0600
**SD**	**h**	**TAS1R 2**	**TAS1R 3**	**TAS2R 31**	**TAS2R 43**	**TAS2R 1**	**TAS2R 38**
	**0**	0.8771	1.7044	0.1986	0.6622	0.1407	0.2920
	**4**	4.3996	1.9238	1.3091	4.9072	1.3677	1.0297
	**8**	2.3371	6.3681	0.2209	1.1210	0.3997	0.2627
	**24**	0.3230	1.5386	0.0585	0.0597	0.0813	0.0751
**SEM**	**h**	**TAS1R 2**	**TAS1R 3**	**TAS2R 31**	**TAS2R 43**	**TAS2R 1**	**TAS2R 38**
	**0**	0.2774	0.5390	0.0628	0.2094	0.0445	0.0923
	**4**	1.3913	0.6083	0.4140	1.5518	0.4325	0.3256
	**8**	0.7391	2.0138	0.0699	0.3545	0.1264	0.0831
	**24**	0.1022	0.4866	0.0185	0.0189	0.0257	0.0237

**SK3**	**3**	4.5428	1.1342	0.0196	0.0218	0.0343	0.0670		
**SK4**	**4**	0.2581	0.0119	0.0549	0.0612	0.0306	0.0328		
**TF1**	**1**	0.3774	0.7371	0.4491	2.2966	0.4605	0.8209		
**TF2**	**2**	1.0722	2.6847	4.4940	16.5702	4.5833	3.4734		
**TF3**	**3**	1.0858	0.6421	0.7680	3.8006	1.3518	0.9029		
**TF4**	**4**	1.0940	0.0788	0.0175	0.0834	0.0254	0.0417		

Sweet and bitter taste receptor gene expression kinetics, determined by RT-qPCR, compared to the mean expression of three housekeeper genes ($2^{-\Delta Cq}$), and displayed as fold-change between water intervention (control) and sweetener mix intervention (*n* = 10; data to Fig. 4).

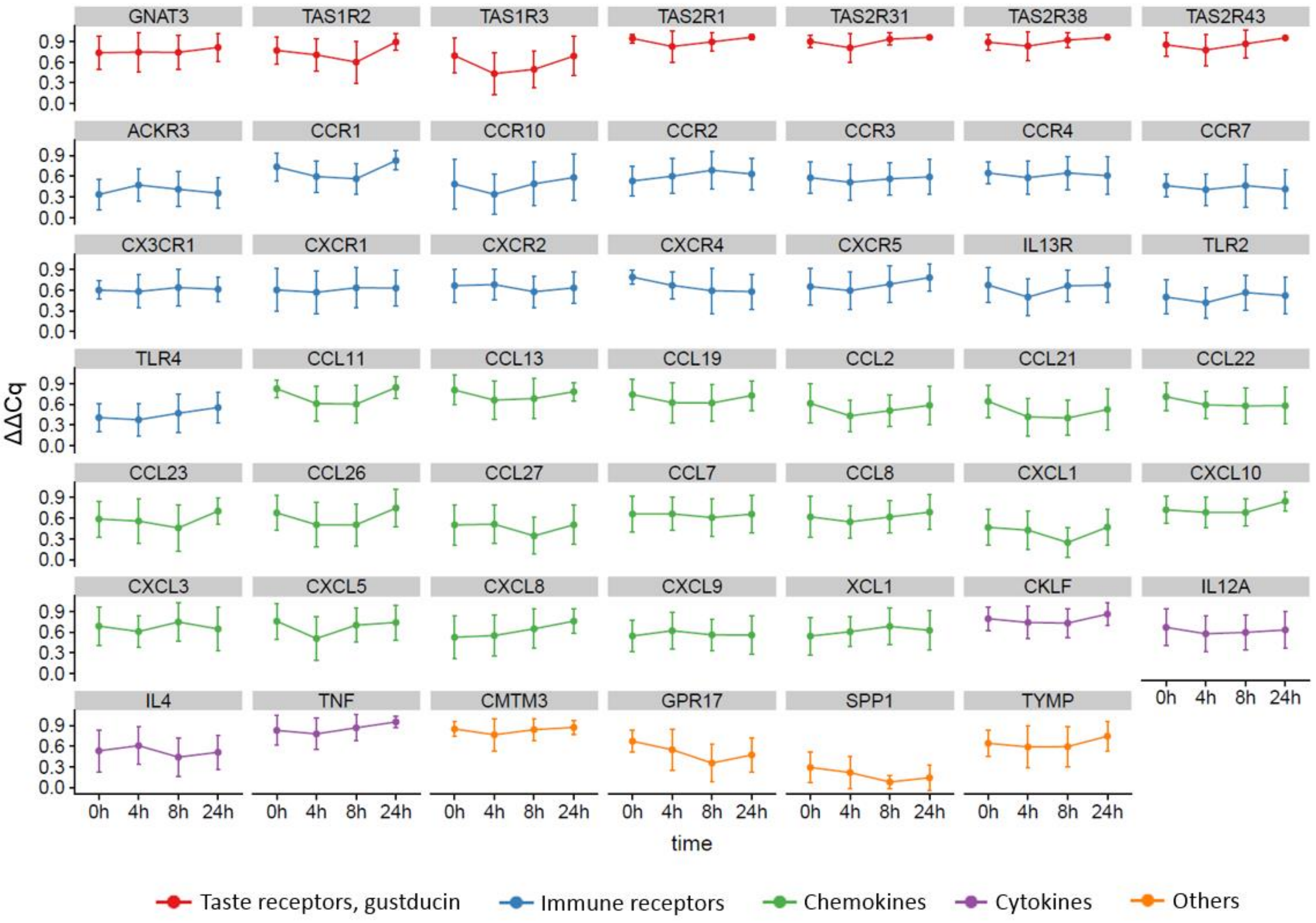

Figure S2: Post-intervention transcript level kinetics of 48 immunity- and chemoreception-relevant genes. Study participants (*n* =10) were challenged with either a food-relevant artificial sweetener mix (acesulfame-K, cyclamate, saccharin) or water. Neutrophils were isolated from blood drawn before (0h) or after (4, 8, 24h) challenge and RNA was extracted. Values are RT-qPCR-derived ΔΔCq, with ΔCq calculated from expression of target- vs. Housekeeper-genes, and ΔΔCq from $\Delta Cq_{treated}$ vs. $\Delta Cq_{untreated}$ (decrease of ΔΔCq as compared to 0h, thus, reflects upregulation of transcription, and vice versa).

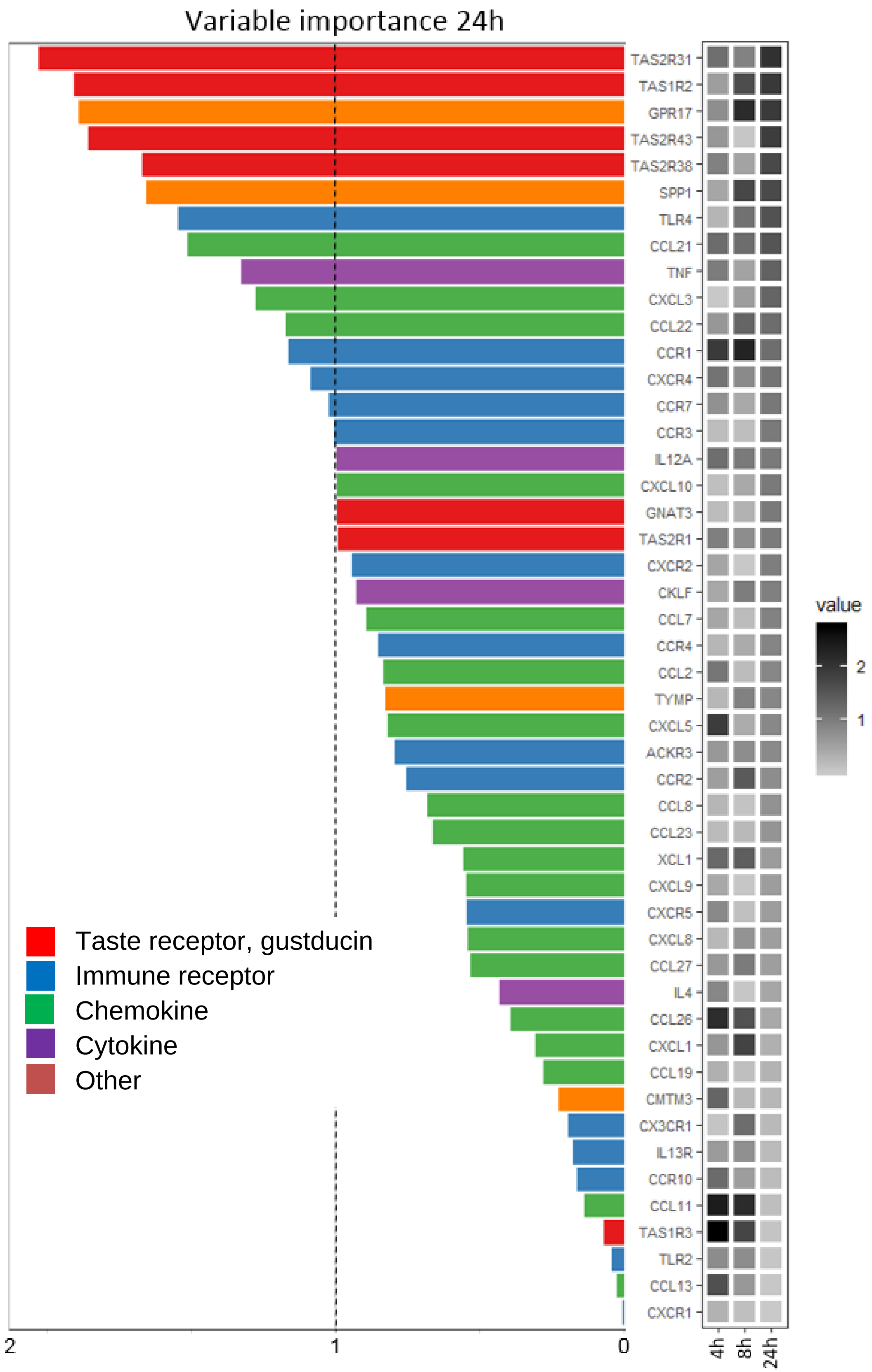

Figure S3: VIP plot of transcriptional data between sweetener mix and water 0 vs. 24 hrs. Colors indicate the group affiliation as indicated in the legend. The plot ranks the analyzed selected taste receptors and cytokines/chemokines according to the variable importance scores (VIP) at the time point 24h (intervention vs. control). This analysis depicts the relevance of each analyte for the separation in the OPLS-DA-plot shown in Fig. 4. Variables above the value of 1 (dashed line) are considered as contributing. The heat map on the right again shows VIP-values in a grey coded manner for all 3 time points (4, 8 and 24h), sorted according to the descending 24h values.

Table S7: VIP-gene lists and accession numbers at 4h, 8h, and 24h post-intervention

4h Sweetener Mix		8h Sweetener Mix		24h Sweetener Mix	
**Gene symbol**	**Accesion #**	**Gene symbol**	**Accesion #**	**Gene Symbol**	**Accesion #**
TAS1R3	NM_152228	CCR1	NM_001295	TAS2R31	NM_176885
CCL11	NM_002986	CCL11	NM_002986	TAS1R2	NM_152232
CCL26	NM_001371936	GPR17	NM_001161415	GPR17	NM_001161415
CCR1	NM_001295	CXCL1	NM_001511	TAS2R43	NM_176884
CXCL5	NM_002994	TAS1R3	NM_152228	TAS2R38	NM_176817
CCL13	NM_005408	SPP1	NM_001251830	SPP1	NM_000582
CMTM3	NM_001363918	TAS1R2	NM_152232	TLR4	NM_003266
XCL1	NM_002995	CCL26	NM_001371936	CCL21	NM_002989
CCR10	NM_016602	CCR2	NM_001123041	TNFα	NM_000594
CCL21	NM_002989	XCL1	NM_002995	CXCL3	NM_002090
IL12A	NM_000882	CCL22	NM_002990	CCL22	NM_002990
TAS2R31	NM_176885	CX3CR1	NM_001171174	CCR1	NM_001295
CXCR4	NM_001008540	CCL21	NM_002989	CXCR4	NM_001008540
CCL2	NM_002982	TLR4	NM_138554	CCR7	NM_001301714
		IL12A	NM_000882	CCR3	NM_001164680
				IL12A	NM_000882
				CXCL10	NM_001565
				GNAT3	NM_001102386

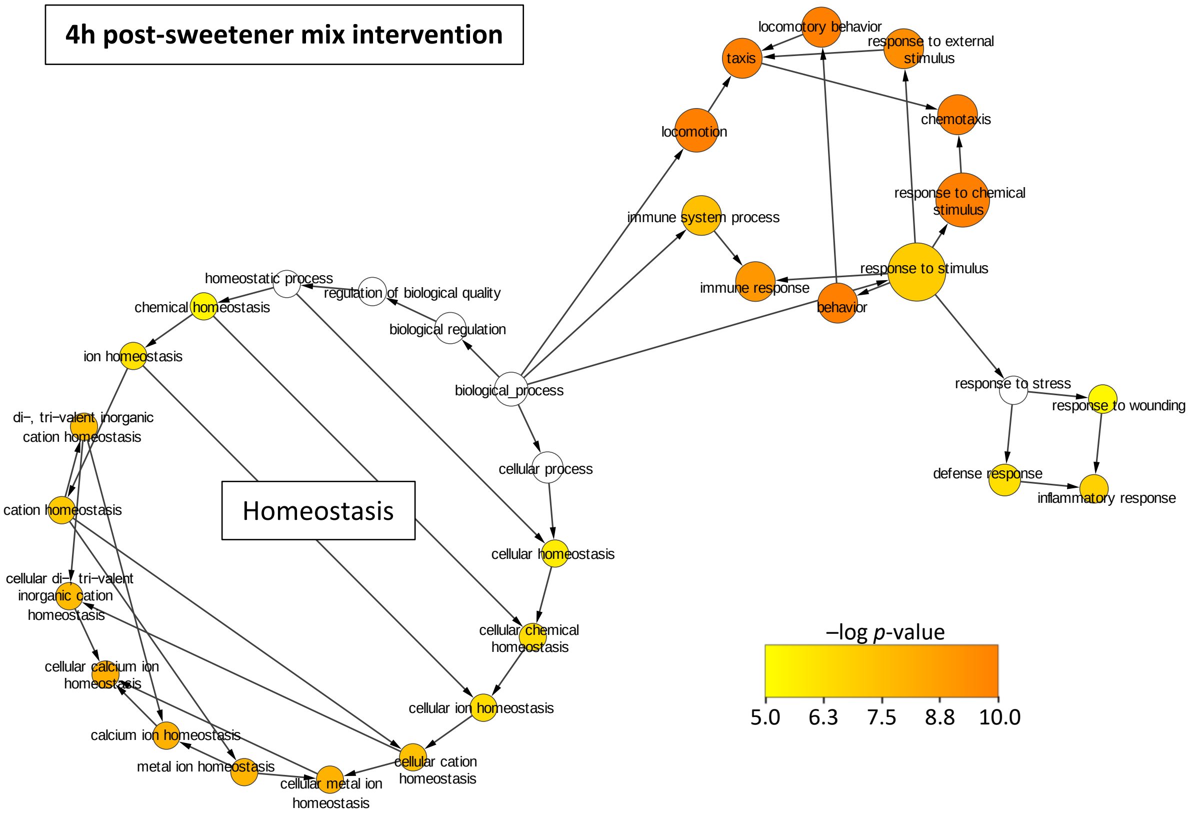

Figure S4: Bingo (v3.0.3) analysis in Cytoscape (v3.7.1) of only the VIP genes relevant for the separation in the OPLS-DA analysis of the **4h** sweetener mix intervention group versus the 0h control group (Figure 4A).

Table S8: BiNGO results for GO-term ‚inflammatory response'

BiNGO Analysis of:	All 48 genes	VIP genes 4h	VIP genes 8h	VIP genes 24h
GO_6954: inflammatory response	CCL11	CCL11|	CCL11	
	CCL13	CCL13|		
	CCL19			
	CCL2	CCL2		
	CCL21	CCL21	CCL21	CCL21
	CCL22		CCL22	CCL22
	CCL23		CCL26	
	CCL26	CCL26		
	CCL7		CCR1	CCR1
	CCL8			
	CCR1	CCR1|		
	CCR2		CCR2	
	CCR3			CCR3
	CCR4			
	CCR7			CCR7
	CXCL1		CXCL1	
	CXCL10			CXCL10
	CXCL3			CXCL3
	CXCL8 (IL-8)			
	CXCL9			
	CXCR1			
	CXCR2			
	CXCR4	CXCR4		CXCR4
	SPP1		SPP1	SPP1
	TLR4		TLR4	TLR4
	TNF			TNF

Table S9: BiNGO results, p-values, and VIP-normalized node-sizes

4h BP's	Normalisierte Σ der VIP values	Node sizes in Cytoscape	VIP-adjusted node size
behavior	1.51	6.32	9.57
biological regulation	1.00	6.63	6.63
biological_process	1.00	7.48	7.48
calcium ion homeostasis	1.00	5.29	5.29
cation homeostasis	1.00	5.29	5.29
cellular calcium ion homeostasis	1.00	5.29	5.29
cellular cation homeostasis	1.00	5.29	5.29
cellular chemical homeostasis	1.00	5.29	5.29
cellular di-, tri-valent inorganic cation homeostasis	1.00	5.29	5.29
cellular homeostasis	1.00	5.29	5.29
cellular ion homeostasis	1.00	5.29	5.29
cellular metal ion homeostasis	1.00	5.29	5.29
cellular process	1.00	6.63	6.63
chemical homeostasis	1.00	5.29	5.29
chemotaxis	1.51	6.32	9.57
defense response	1.19	5.66	6.76
di-, tri-valent inorganic cation homeostasis	1.00	5.29	5.29
homeostatic process	1.00	5.29	5.29
immune response	1.50	6.32	9.50
immune system process	1.50	6.32	9.50
inflammatory response	1.08	5.29	5.73
ion homeostasis	1.00	5.29	5.29
locomotion	1.63	6.63	10.78
locomotory behavior	1.51	6.32	9.57
metal ion homeostasis	1.00	5.29	5.29
regulation of biological quality	1.00	5.66	5.66
response to chemical stimulus	1.99	7.21	14.36
response to external stimulus	1.51	6.32	9.57
response to stimulus	2.10	7.48	15.72
response to stress	1.00	5.66	5.66
response to wounding	1.08	5.29	5.73
taxis	1.51	6.32	9.57
**8h BPs**	**Normalisierte Σ der VIP values**	**Node sizes in cytoscape**	**VIP-adjusted node size**
behavior	1.14	6.00	6.85
biological process	1	7.75	7.75
chemotaxis	1.14	6.00	6.85
defense response	1.25	6.63	8.31
immune response	1.31	6.63	8.72
immune system process	1.31	6.63	8.72
inflammatory response	1.16	6.00	6.98
locomotion	1.22	6.32	7.74
locomotory behavior	1.14	6.00	6.85
response to chemical stimulus	1.72	7.48	12.89
response to external stimulus	1.37	6.63	9.09
response to stimulus	1.72	7.48	12.89
response to stress	1.34	6.63	8.89
response to wounding	1.26	6.32	7.96
signaling	1.68	7.21	12.08
taxis	1.00	6.00	6.00

24h BP's	Normalisierte Σ der VIP values	Node sizes in Cytoscape	VIP-adjusted node size
behavior	1.00	5.29	5.29
biological_process	1.00	8.49	8.49
chemotaxis	1.00	5.29	5.29
cognition	1.00	4.47	4.47
defense response	1.81	6.93	12.55
immune response	1.35	6.00	8.11
immune system process	1.00	6.00	6.00
inflammatory response	1.69	6.63	11.21
locomotion	1.29	6.00	7.71
locomotory behavior	1.00	5.29	5.29
multicellular organismal process	1.00	6.00	6.00
neurological system process	1.00	4.47	4.47
response to chemical stimulus	2.40	7.75	18.56
response to external stimulus	1.56	6.32	9.84
response to stimulus	2.85	8.25	23.50
response to stress	1.81	6.93	12.55
response to wounding	1.69	6.63	11.21
sensory perception	1.00	4.47	4.47
sensory perception of chemical stimulus	1.00	4.47	4.47
sensory perception of taste	1.04	4.47	4.64
system process	1.00	5.29	5.29
taxis	1.00	5.29	5.29

Table S10 – a): ClueGO results_4h sweetener mix intervention

GO ID	GO Term	Ontology Source	Term*p* Value	Term *p*Value Corrected with Bonferroni step down	Group*p* Value	Group *p*Value Corrected with Bonferroni step down	GO Levels	GO Groups	% Associated Genes	Nr. Genes	Associated Genes Found
GO:0036336	dendritic cell migration	GO_BiologicalProcess-EBI-UniProt-GOA_01.05.2020_00h00	1.25E-08	8.77E-08	1.25E-08	2.51E-08	[3, 5, 6]	Group0	11.11	4.00	[CCL21, CCR1, CXCR4, IL12A]
GO:0002407	dendritic cell chemotaxis	GO_BiologicalProcess-EBI-UniProt-GOA_01.05.2020_00h00	5.85E-09	4.68E-08	1.25E-08	2.51E-08	[4, 6, 7]	Group0	13.33	4.00	[CCL21, CCR1, CXCR4, IL12A]
GO:1990868	response to chemokine	GO_BiologicalProcess-EBI-UniProt-GOA_01.05.2020_00h00	1.40E-19	2.10E-18	1.40E-19	5.61E-19	[5]	Group1	8.06	10.00	[CCL11, CCL13, CCL2, CCL21, CCL26, CCR1, CCR10, CXCL5, CXCR4, XCL1]
GO:0070098	chemokine-mediated signaling pathway	GO_BiologicalProcess-EBI-UniProt-GOA_01.05.2020_00h00	5.86E-20	9.38E-19	1.40E-19	5.61E-19	[5, 6, 7]	Group1	8.77	10.00	[CCL11, CCL13, CCL2, CCL21, CCL26, CCR1, CCR10, CXCL5, CXCR4, XCL1]
GO:1990869	cellular response to chemokine	GO_BiologicalProcess-EBI-UniProt-GOA_01.05.2020_00h00	1.40E-19	2.10E-18	1.40E-19	5.61E-19	[6]	Group1	8.06	10.00	[CCL11, CCL13, CCL2, CCL21, CCL26, CCR1, CCR10, CXCL5, CXCR4, XCL1]
GO:0001637	G protein-coupled chemoattractant receptor activity	GO_BiologicalProcess-EBI-UniProt-GOA_01.05.2020_00h00	1.75E-06	3.51E-06	1.75E-06	1.75E-06	[6, 7, 8]	Group2	9.38	3.00	[CCR1, CCR10, CXCR4]
GO:0004950	chemokine receptor activity	GO_BiologicalProcess-EBI-UniProt-GOA_01.05.2020_00h00	1.75E-06	3.51E-06	1.75E-06	1.75E-06	[6, 7, 8, 9]	Group2	9.38	3.00	[CCR1, CCR10, CXCR4]
GO:0016493	C-C chemokine receptor activity	GO_BiologicalProcess-EBI-UniProt-GOA_01.05.2020_00h00	1.29E-06	5.18E-06	1.75E-06	1.75E-06	[7, 8, 9, 10]	Group2	10.34	3.00	[CCR1, CCR10, CXCR4]
GO:0071674	mononuclear cell migration	GO_BiologicalProcess-EBI-UniProt-GOA_01.05.2020_00h00	2.19E-12	2.63E-11	3.06E-11	9.18E-11	[3, 5, 6]	Group3	5.51	7.00	[CCL11, CCL13, CCL2, CCL21, CCL26, CCR1, XCL1]
GO:0002548	monocyte chemotaxis	GO_BiologicalProcess-EBI-UniProt-GOA_01.05.2020_00h00	1.85E-13	2.41E-12	3.06E-11	9.18E-11	[4, 6, 7]	Group3	7.78	7.00	[CCL11, CCL13, CCL2, CCL21, CCL26, CCR1, XCL1]
GO:0048247	lymphocyte chemotaxis	GO_BiologicalProcess-EBI-UniProt-GOA_01.05.2020_00h00	1.98E-11	1.78E-10	3.06E-11	9.18E-11	[4, 6, 7]	Group3	7.32	6.00	[CCL11, CCL13, CCL2, CCL21, CCL26, XCL1]
GO:0072678	T cell migration	GO_BiologicalProcess-EBI-UniProt-GOA_01.05.2020_00h00	6.06E-07	3.03E-06	3.06E-11	9.18E-11	[4, 6, 7]	Group3	4.30	4.00	[CCL2, CCL21, CCL26, XCL1]
GO:0008009	chemokine activity	GO_BiologicalProcess-EBI-UniProt-GOA_01.05.2020_00h00	1.74E-14	2.44E-13	3.06E-11	9.18E-11	[5, 6, 7, 8]	Group3	10.77	7.00	[CCL11, CCL13, CCL2, CCL21, CCL26, CXCL5, XCL1]
GO:0010818	T cell chemotaxis	GO_BiologicalProcess-EBI-UniProt-GOA_01.05.2020_00h00	2.31E-06	2.31E-06	3.06E-11	9.18E-11	[5, 7, 8]	Group3	8.57	3.00	[CCL21, CCL26, XCL1]
GO:0072677	eosinophil migration	GO_BiologicalProcess-EBI-UniProt-GOA_01.05.2020_00h00	1.75E-08	1.05E-07	3.06E-11	9.18E-11	[5, 7, 8]	Group3	10.26	4.00	[CCL11, CCL13, CCL2, XCL1]
GO:1990266	neutrophil migration	GO_BiologicalProcess-EBI-UniProt-GOA_01.05.2020_00h00	1.47E-11	1.47E-10	3.06E-11	9.18E-11	[5, 7, 8]	Group3	4.22	7.00	[CCL11, CCL13, CCL2, CCL21, CCL26, CXCL5, XCL1]
GO:0030593	neutrophil chemotaxis	GO_BiologicalProcess-EBI-UniProt-GOA_01.05.2020_00h00	5.62E-12	6.18E-11	3.06E-11	9.18E-11	[5, 6, 7, 8, 9]	Group3	4.83	7.00	[CCL11, CCL13, CCL2, CCL21, CCL26, CXCL5, XCL1]
GO:0048245	eosinophil chemotaxis	GO_BiologicalProcess-EBI-UniProt-GOA_01.05.2020_00h00	5.85E-09	4.68E-08	3.06E-11	9.18E-11	[5, 6, 7, 8, 9]	Group3	13.33	4.00	[CCL11, CCL13, CCL2, XCL1]
GO:1901623	regulation of lymphocyte chemotaxis	GO_BiologicalProcess-EBI-UniProt-GOA_01.05.2020_00h00	1.59E-06	4.77E-06	3.06E-11	9.18E-11	[5, 6, 7, 8, 9]	Group3	9.68	3.00	[CCL2, CCL21, XCL1]

Table S10 – b): ClueGO results_8h sweetener mix intervention

GO ID	GO Term	Ontology Source	Term p Value	Term p Value Corrected with Bonferroni step down	Group p Value	Group P Value Corrected with Bonferroni step down	GO Levels	GO Groups	% Associ-ated Genes	Nr. Genes	Associated Genes Found
GO:0061756	leukocyte adhesion to vascular endothelial cell	GO_BiologicalProcess-EBI-UniProt-GOA_01.05.2020_00h00	8.58E-06	3.43E-05	3.92E-10	3.92E-10	[5]	Group0	6.00	3.00	[CCL21, CCR2, CX3CR1]
GO:0050901	leukocyte tethering or rolling	GO_BiologicalProcess-EBI-UniProt-GOA_01.05.2020_00h00	2.64E-06	1.85E-05	3.92E-10	3.92E-10	[4, 6, 7]	Group0	8.82	3.00	[CCL21, CCR2, CX3CR1]
GO:0042533	tumor necrosis factor biosynthetic process	GO_BiologicalProcess-EBI-UniProt-GOA_01.05.2020_00h00	7.11E-06	3.55E-05	3.92E-10	3.92E-10	[4, 5, 6, 7, 8]	Group0	6.38	3.00	[CCR2, CX3CR1, TLR4]
GO:0001637	G protein-coupled chemoattractant receptor activity	GO_BiologicalProcess-EBI-UniProt-GOA_01.05.2020_00h00	1.04E-08	1.88E-07	3.92E-10	3.92E-10	[6, 7, 8]	Group0	12.50	4.00	[CCR1, CCR2, CX3CR1, GPR17]
GO:0042534	regulation of tumor necrosis factor biosynthetic process	GO_BiologicalProcess-EBI-UniProt-GOA_01.05.2020_00h00	7.11E-06	3.55E-05	3.92E-10	3.92E-10	[5, 6, 7, 8, 9]	Group0	6.38	3.00	[CCR2, CX3CR1, TLR4]
GO:0004950	chemokine receptor activity	GO_BiologicalProcess-EBI-UniProt-GOA_01.05.2020_00h00	1.04E-08	1.88E-07	3.92E-10	3.92E-10	[6, 7, 8, 9]	Group0	12.50	4.00	[CCR1, CCR2, CX3CR1, GPR17]
GO:0016493	C-C chemokine receptor activity	GO_BiologicalProcess-EBI-UniProt-GOA_01.05.2020_00h00	1.62E-06	1.45E-05	3.92E-10	3.92E-10	[7, 8, 9, 10]	Group0	10.34	3.00	[CCR1, CCR2, CX3CR1]
GO:0036336	dendritic cell migration	GO_BiologicalProcess-EBI-UniProt-GOA_01.05.2020_00h00	1.71E-08	2.73E-07	1.21E-16	2.42E-16	[3, 5, 6]	Group1	11.11	4.00	[CCL21, CCR1, CCR2, IL12A]
GO:0071674	mononuclear cell migration	GO_BiologicalProcess-EBI-UniProt-GOA_01.05.2020_00h00	4.08E-12	9.39E-11	1.21E-16	2.42E-16	[3, 5, 6]	Group1	5.51	7.00	[CCL11, CCL21, CCL22, CCL26, CCR1, CCR2, XCL1]
GO:0042088	T-helper 1 type immune response	GO_BiologicalProcess-EBI-UniProt-GOA_01.05.2020_00h00	1.49E-07	2.09E-06	1.21E-16	2.42E-16	[5]	Group1	6.56	4.00	[CCR2, IL12A, TLR4, XCL1]
GO:0061756	leukocyte adhesion to vascular endothelial cell	GO_BiologicalProcess-EBI-UniProt-GOA_01.05.2020_00h00	8.58E-06	3.43E-05	1.21E-16	2.42E-16	[5]	Group1	6.00	3.00	[CCL21, CCR2, CX3CR1]
GO:1990868	response to chemokine	GO_BiologicalProcess-EBI-UniProt-GOA_01.05.2020_00h00	4.19E-19	1.05E-17	1.21E-16	2.42E-16	[5]	Group1	8.06	10.00	[CCL11, CCL21, CCL22, CCL26, CCR1, CCR2, CX3CR1, CXCL1, GPR17, XCL1]
GO:0002369	T cell cytokine production	GO_BiologicalProcess-EBI-UniProt-GOA_01.05.2020_00h00	1.28E-05	2.55E-05	1.21E-16	2.42E-16	[4, 6, 7]	Group1	5.26	3.00	[CCR2, IL12A, XCL1]
GO:0002407	dendritic cell chemotaxis	GO_BiologicalProcess-EBI-UniProt-GOA_01.05.2020_00h00	7.96E-09	1.51E-07	1.21E-16	2.42E-16	[4, 6, 7]	Group1	13.33	4.00	[CCL21, CCR1, CCR2, IL12A]
GO:0002548	monocyte chemotaxis	GO_BiologicalProcess-EBI-UniProt-GOA_01.05.2020_00h00	3.46E-13	8.31E-12	1.21E-16	2.42E-16	[4, 6, 7]	Group1	7.78	7.00	[CCL11, CCL21, CCL22, CCL26, CCR1, CCR2, XCL1]
GO:0048247	lymphocyte chemotaxis	GO_BiologicalProcess-EBI-UniProt-GOA_01.05.2020_00h00	3.29E-11	6.91E-10	1.21E-16	2.42E-16	[4, 6, 7]	Group1	7.32	6.00	[CCL11, CCL21, CCL22, CCL26, CCR2, XCL1]
GO:0050901	leukocyte tethering or rolling	GO_BiologicalProcess-EBI-UniProt-GOA_01.05.2020_00h00	2.64E-06	1.85E-05	1.21E-16	2.42E-16	[4, 6, 7]	Group1	8.82	3.00	[CCL21, CCR2, CX3CR1]
GO:0072678	T cell migration	GO_BiologicalProcess-EBI-UniProt-GOA_01.05.2020_00h00	8.23E-07	9.05E-06	1.21E-16	2.42E-16	[4, 6, 7]	Group1	4.30	4.00	[CCL21, CCL26, CCR2, XCL1]
GO:0042533	tumor necrosis factor biosynthetic process	GO_BiologicalProcess-EBI-UniProt-GOA_01.05.2020_00h00	7.11E-06	3.55E-05	1.21E-16	2.42E-16	[4, 5, 6, 7, 8]	Group1	6.38	3.00	[CCR2, CX3CR1, TLR4]
GO:0070098	chemokine-mediated signaling pathway	GO_BiologicalProcess-EBI-UniProt-GOA_01.05.2020_00h00	1.75E-19	4.55E-18	1.21E-16	2.42E-16	[5, 6, 7]	Group1	8.77	10.00	[CCL11, CCL21, CCL22, CCL26, CCR1, CCR2, CX3CR1, CXCL1, GPR17, XCL1]
GO:1990869	cellular response to chemokine	GO_BiologicalProcess-EBI-UniProt-GOA_01.05.2020_00h00	4.19E-19	1.05E-17	1.21E-16	2.42E-16	[6]	Group1	8.06	10.00	[CCL11, CCL21, CCL22, CCL26, CCR1, CCR2, CX3CR1, CXCL1, GPR17, XCL1]
GO:0002690	positive regulation of leukocyte chemotaxis	GO_BiologicalProcess-EBI-UniProt-GOA_01.05.2020_00h00	3.93E-08	5.90E-07	1.21E-16	2.42E-16	[4, 5, 6, 7, 8, 9]	Group1	4.00	5.00	[CCL21, CCR1, CCR2, IL12A, XCL1]
GO:0008009	chemokine activity	GO_BiologicalProcess-EBI-UniProt-GOA_01.05.2020_00h00	7.82E-12	1.72E-10	1.21E-16	2.42E-16	[5, 6, 7, 8]	Group1	9.23	6.00	[CCL11, CCL21, CCL22, CCL26, CXCL1, XCL1]
GO:2000403	positive regulation of lymphocyte migration	GO_BiologicalProcess-EBI-UniProt-GOA_01.05.2020_00h00	9.11E-06	2.73E-05	1.21E-16	2.42E-16	[4, 5, 6, 7, 8, 9]	Group1	5.88	3.00	[CCL21, CCR2, XCL1]
GO:0010818	T cell chemotaxis	GO_BiologicalProcess-EBI-UniProt-GOA_01.05.2020_00h00	1.52E-08	2.58E-07	1.21E-16	2.42E-16	[5, 7, 8]	Group1	11.43	4.00	[CCL21, CCL26, CCR2, XCL1]
GO:0030593	neutrophil chemotaxis	GO_BiologicalProcess-EBI-UniProt-GOA_01.05.2020_00h00	1.06E-09	2.13E-08	1.21E-16	2.42E-16	[5, 6, 7, 8, 9]	Group1	4.14	6.00	[CCL11, CCL21, CCL22, CCL26, CXCL1, XCL1]
GO:0042534	regulation of tumor necrosis factor biosynthetic process	GO_BiologicalProcess-EBI-UniProt-GOA_01.05.2020_00h00	7.11E-06	3.55E-05	1.21E-16	2.42E-16	[5, 6, 7, 8, 9]	Group1	6.38	3.00	[CCR2, CX3CR1, TLR4]
GO:1901623	regulation of lymphocyte chemotaxis	GO_BiologicalProcess-EBI-UniProt-GOA_01.05.2020_00h00	1.99E-06	1.59E-05	1.21E-16	2.42E-16	[5, 6, 7, 8, 9]	Group1	9.68	3.00	[CCL21, CCR2, XCL1]
GO:2000404	regulation of T cell migration	GO_BiologicalProcess-EBI-UniProt-GOA_01.05.2020_00h00	1.49E-05	1.49E-05	1.21E-16	2.42E-16	[5, 6, 7, 8, 9]	Group1	5.00	3.00	[CCL21, CCR2, XCL1]
GO:0140131	positive regulation of lymphocyte chemotaxis	GO_BiologicalProcess-EBI-UniProt-GOA_01.05.2020_00h00	1.15E-06	1.15E-05	1.21E-16	2.42E-16	[5, 6, 7, 8, 9, 10]	Group1	11.54	3.00	[CCL21, CCR2, XCL1]
GO:2000406	positive regulation of T cell migration	GO_BiologicalProcess-EBI-UniProt-GOA_01.05.2020_00h00	4.69E-06	2.81E-05	1.21E-16	2.42E-16	[5, 6, 7, 8, 9, 10]	Group1	7.32	3.00	[CCL21, CCR2, XCL1]
GO:0010819	regulation of T cell chemotaxis	GO_BiologicalProcess-EBI-UniProt-GOA_01.05.2020_00h00	4.31E-07	5.17E-06	1.21E-16	2.42E-16	[6, 7, 8, 9, 10]	Group1	15.79	3.00	[CCL21, CCR2, XCL1]
GO:0010820	positive regulation of T cell chemotaxis	GO_BiologicalProcess-EBI-UniProt-GOA_01.05.2020_00h00	3.63E-07	4.72E-06	1.21E-16	2.42E-16	[6, 7, 8, 9, 10, 11]	Group1	16.67	3.00	[CCL21, CCR2, XCL1]
GO:0016493	C-C chemokine receptor activity	GO_BiologicalProcess-EBI-UniProt-GOA_01.05.2020_00h00	1.62E-06	1.45E-05	1.21E-16	2.42E-16	[7, 8, 9, 10]	Group1	10.34	3.00	[CCR1, CCR2, CX3CR1]

Table S10 – c): ClueGO results_24h sweetener mix intervention

GOID	GOTerm	Ontology Source	Term PValue	Term PValue Corrected with Bonferroni step down	Group PValue	Group PValue Corrected with Bonferroni step down	GOLevels	GOGroups	% Associated Genes	Nr. Genes	Associated Genes Found
GO:000243	inflammatory response to antigenic stimulus	GO_BiologicalProcess-EBI-UniProt-GO	4.98E-05	4.98E-05	4.98E-05	4.98E-05	[3, 5]	Group0	4.05	3.00	[CCR7, GPR17, TNF]
GO:000286	regulation of inflammatory response to antigenic stimulus	GO_BiologicalProcess-EBI-UniProt-GO	5.15E-06	2.06E-05	4.98E-05	4.98E-05	[4, 5, 6, 7]	Group0	8.57	3.00	[CCR7, GPR17, TNF]
GO:005090	sensory perception of taste	GO_BiologicalProcess-EBI-UniProt-GO	1.40E-08	2.38E-07	1.40E-08	2.80E-08	[6]	Group1	6.02	5.00	[GNAT3, TAS1R2, TAS2R31, TAS2R38, TAS2R43]
GO:005091	detection of chemical stimulus involved in sensory perception of taste	GO_BiologicalProcess-EBI-UniProt-GO	3.11E-07	2.80E-06	1.40E-08	2.80E-08	[5, 7]	Group1	6.67	4.00	[TAS1R2, TAS2R31, TAS2R38, TAS2R43]
GO:000158	detection of chemical stimulus involved in sensory perception of bitter taste	GO_BiologicalProcess-EBI-UniProt-GO	1.11E-05	3.33E-05	1.40E-08	2.80E-08	[6, 8]	Group1	6.67	3.00	[TAS2R31, TAS2R38, TAS2R43]
GO:000852	taste receptor activity	GO_BiologicalProcess-EBI-UniProt-GO	5.31E-08	7.97E-07	1.40E-08	2.80E-08	[6, 8]	Group1	10.26	4.00	[TAS1R2, TAS2R31, TAS2R38, TAS2R43]
GO:005091	sensory perception of bitter taste	GO_BiologicalProcess-EBI-UniProt-GO	1.48E-07	1.48E-06	1.40E-08	2.80E-08	[7]	Group1	8.00	4.00	[GNAT3, TAS2R31, TAS2R38, TAS2R43]
GO:003303	bitter taste receptor activity	GO_BiologicalProcess-EBI-UniProt-GO	1.60E-06	9.63E-06	1.40E-08	2.80E-08	[7, 9]	Group1	12.50	3.00	[TAS2R31, TAS2R38, TAS2R43]
GO:003633	dendritic cell migration	GO_BiologicalProcess-EBI-UniProt-GO	1.87E-10	3.37E-09	3.72E-15	1.12E-14	[3, 5, 6]	Group2	13.89	5.00	[CCL21, CCR1, CCR7, CXCR4, IL12A]
GO:199086	response to chemokine	GO_BiologicalProcess-EBI-UniProt-GO	1.05E-15	2.21E-14	3.72E-15	1.12E-14	[5]	Group2	7.26	9.00	[CCL21, CCL22, CCR1, CCR3, CCR7, CXCL10, CXCL3, CXCR4, GPR17]
GO:000240	dendritic cell chemotaxis	GO_BiologicalProcess-EBI-UniProt-GO	7.10E-11	1.42E-09	3.72E-15	1.12E-14	[4, 6, 7]	Group2	16.67	5.00	[CCL21, CCR1, CCR7, CXCR4, IL12A]
GO:000254	monocyte chemotaxis	GO_BiologicalProcess-EBI-UniProt-GO	1.60E-06	1.12E-05	3.72E-15	1.12E-14	[4, 6, 7]	Group2	4.44	4.00	[CCL21, CCL22, CCR1, CXCL10]
GO:000489	cytokine receptor activity	GO_BiologicalProcess-EBI-UniProt-GO	7.93E-08	1.03E-06	3.72E-15	1.12E-14	[5, 6, 7]	Group2	4.27	5.00	[CCR1, CCR3, CCR7, CXCR4, GPR17]
GO:007009	chemokine-mediated signaling pathway	GO_BiologicalProcess-EBI-UniProt-GO	4.83E-16	1.06E-14	3.72E-15	1.12E-14	[5, 6, 7]	Group2	7.89	9.00	[CCL21, CCL22, CCR1, CCR3, CCR7, CXCL10, CXCL3, CXCR4, GPR17]
GO:007167	regulation of mononuclear cell migration	GO_BiologicalProcess-EBI-UniProt-GO	4.22E-05	8.44E-05	3.72E-15	1.12E-14	[4, 5, 6, 7, 8]	Group2	4.29	3.00	[CCR1, CXCL10, TNF]
GO:199086	cellular response to chemokine	GO_BiologicalProcess-EBI-UniProt-GO	1.05E-15	2.21E-14	3.72E-15	1.12E-14	[6]	Group2	7.26	9.00	[CCL21, CCL22, CCR1, CCR3, CCR7, CXCL10, CXCL3, CXCR4, GPR17]
GO:000269	positive regulation of leukocyte chemotaxis	GO_BiologicalProcess-EBI-UniProt-GO	1.10E-07	1.21E-06	3.72E-15	1.12E-14	[4, 5, 6, 7, 8, 9]	Group2	4.00	5.00	[CCL21, CCR1, CCR7, CXCL10, IL12A]
GO:000800	chemokine activity	GO_BiologicalProcess-EBI-UniProt-GO	4.30E-07	3.44E-06	3.72E-15	1.12E-14	[5, 6, 7, 8]	Group2	6.15	4.00	[CCL21, CCL22, CXCL10, CXCL3]
GO:007167	positive regulation of mononuclear cell migration	GO_BiologicalProcess-EBI-UniProt-GO	4.30E-06	2.15E-05	3.72E-15	1.12E-14	[4, 5, 6, 7, 8, 9]	Group2	9.09	3.00	[CCR1, CXCL10, TNF]
GO:000163	G protein-coupled chemoattractant receptor activity	GO_BiologicalProcess-EBI-UniProt-GO	1.00E-10	1.90E-09	3.72E-15	1.12E-14	[6, 7, 8]	Group2	15.63	5.00	[CCR1, CCR3, CCR7, CXCR4, GPR17]
GO:200050	regulation of dendritic cell chemotaxis	GO_BiologicalProcess-EBI-UniProt-GO	9.60E-08	1.15E-06	3.72E-15	1.12E-14	[5, 6, 7, 8, 9]	Group2	30.00	3.00	[CCL21, CCR7, IL12A]
GO:000495	chemokine receptor activity	GO_BiologicalProcess-EBI-UniProt-GO	1.00E-10	1.90E-09	3.72E-15	1.12E-14	[6, 7, 8, 9]	Group2	15.63	5.00	[CCR1, CCR3, CCR7, CXCR4, GPR17]
GO:200051	positive regulation of dendritic cell chemotaxis	GO_BiologicalProcess-EBI-UniProt-GO	6.72E-08	9.41E-07	3.72E-15	1.12E-14	[5, 6, 7, 8, 9, 10]	Group2	33.33	3.00	[CCL21, CCR7, IL12A]
GO:001649	C-C chemokine receptor activity	GO_BiologicalProcess-EBI-UniProt-GO	1.54E-08	2.47E-07	3.72E-15	1.12E-14	[7, 8, 9, 10]	Group2	13.79	4.00	[CCR1, CCR3, CCR7, CXCR4]

Table S11: ClueGO results_all 48 genes

GOID	GOTerm	Ontology Source	Term PValue	Term PValue Corrected with Bonferroni step down	Group PValue	Group PValue Corrected with Bonferroni step down	GOLevels	GOGroups	% Associated Genes	Nr. Genes	Associated Genes Found
GO:000504	scavenger receptor activity	GO_BiologicalProcess-EBI-UniProt-	7.74E-04	3.10E-03	7.74E-04	7.74E-04	[5, 7]	Group00	4.29	3.00	[ACKR3, CCR10, CX3CR1]
GO:000696	cellular defense response	GO_BiologicalProcess-EBI-UniProt-	6.16E-07	4.19E-05	6.16E-07	1.23E-06	[4]	Group01	7.81	5.00	[CCR2, CCR3, CX3CR1, CXCL9, CXCR2]
GO:006184	antimicrobial humoral immune response mediated by antimicrobial peptide	GO_BiologicalProcess-EBI-UniProt-	1.38E-08	1.11E-06	9.60E-28	1.34E-26	[4, 5, 6]	Group02	6.25	7.00	[CCL13, CXCL1, CXCL10, CXCL3, CXCL5, CXCL8, CXCL9]
GO:007122	cellular response to lipopolysaccharide	GO_BiologicalProcess-EBI-UniProt-	6.50E-12	6.37E-10	9.60E-28	1.34E-26	[5, 6, 8]	Group02	4.14	12.00	[CCL2, CX3CR1, CXCL1, CXCL10, CXCL3, CXCL5, CXCL8, CXCL9, IL12A, TLR2, TLR4, TNF]
GO:000800	chemokine activity	GO_BiologicalProcess-EBI-UniProt-	1.05E-35	1.39E-33	9.60E-28	1.34E-26	[5, 6, 7, 8]	Group02	29.23	19.00	[CCL11, CCL13, CCL19, CCL2, CCL21, CCL22, CCL23, CCL26, CCL27, CCL7, CCL8, CKLF, CXCL1, CXCL10, CXCL3, CXCL5, CXCL8, CXCL9, XCL1]
GO:000243	inflammatory response to antigenic stimulus	GO_BiologicalProcess-EBI-UniProt-	3.88E-05	1.43E-03	2.66E-07	1.06E-06	[3, 5]	Group03	5.41	4.00	[CCR7, CXCR2, GPR17, TNF]
GO:000286	regulation of inflammatory response to antigenic stimulus	GO_BiologicalProcess-EBI-UniProt-	9.86E-05	2.37E-03	2.66E-07	1.06E-06	[4, 5, 6, 7]	Group03	8.57	3.00	[CCR7, GPR17, TNF]
GO:000267	regulation of acute inflammatory response	GO_BiologicalProcess-EBI-UniProt-	4.93E-04	5.91E-03	2.66E-07	1.06E-06	[5, 6, 7]	Group03	5.00	3.00	[CCR7, IL4, TNF]
GO:200010	negative regulation of leukocyte apoptotic process	GO_BiologicalProcess-EBI-UniProt-	2.32E-05	9.51E-04	2.66E-07	1.06E-06	[6, 7, 8]	Group03	6.15	4.00	[CCL19, CCL21, CCR7, CXCR2]
GO:004512	cellular extravasation	GO_BiologicalProcess-EBI-UniProt-	2.55E-06	1.48E-04	1.44E-07	7.20E-07	[3, 5, 6]	Group04	5.88	5.00	[CCL2, CCL21, CCR2, CX3CR1, TNF]
GO:000193	negative regulation of endothelial cell proliferation	GO_BiologicalProcess-EBI-UniProt-	3.81E-04	4.96E-03	1.44E-07	7.20E-07	[5, 6, 7]	Group04	5.45	3.00	[CCL2, IL12A, TNF]
GO:003272	positive regulation of interferon-gamma production	GO_BiologicalProcess-EBI-UniProt-	5.81E-05	1.86E-03	1.44E-07	7.20E-07	[4, 5, 6, 7, 8]	Group04	4.88	4.00	[CCR2, IL12A, TLR4, TNF]
GO:003166	lipopolysaccharide-mediated signaling pathway	GO_BiologicalProcess-EBI-UniProt-	7.00E-05	1.96E-03	1.44E-07	7.20E-07	[4, 5, 6, 7, 9]	Group04	4.65	4.00	[CCL2, TLR2, TLR4, TNF]
GO:200035	regulation of endothelial cell apoptotic process	GO_BiologicalProcess-EBI-UniProt-	6.52E-04	5.22E-03	1.44E-07	7.20E-07	[7, 8]	Group04	4.55	3.00	[CCL2, IL4, TNF]
GO:200105	reactive nitrogen species metabolic process	GO_BiologicalProcess-EBI-UniProt-	1.21E-04	2.54E-03	8.01E-11	5.61E-10	[3]	Group05	4.04	4.00	[CX3CR1, TLR2, TLR4, TNF]
GO:004620	nitric oxide metabolic process	GO_BiologicalProcess-EBI-UniProt-	1.07E-04	2.36E-03	8.01E-11	5.61E-10	[4, 5]	Group05	4.17	4.00	[CX3CR1, TLR2, TLR4, TNF]
GO:006184	antimicrobial humoral immune response mediated by antimicrobial peptide	GO_BiologicalProcess-EBI-UniProt-	1.38E-08	1.11E-06	8.01E-11	5.61E-10	[4, 5, 6]	Group05	6.25	7.00	[CCL13, CXCL1, CXCL10, CXCL3, CXCL5, CXCL8, CXCL9]
GO:003166	lipopolysaccharide-mediated signaling pathway	GO_BiologicalProcess-EBI-UniProt-	7.00E-05	1.96E-03	8.01E-11	5.61E-10	[4, 5, 6, 7, 9]	Group05	4.65	4.00	[CCL2, TLR2, TLR4, TNF]
GO:007122	cellular response to lipopolysaccharide	GO_BiologicalProcess-EBI-UniProt-	6.50E-12	6.37E-10	8.01E-11	5.61E-10	[5, 6, 8]	Group05	4.14	12.00	[CCL2, CX3CR1, CXCL1, CXCL10, CXCL3, CXCL5, CXCL8, CXCL9, IL12A, TLR2, TLR4, TNF]
GO:000725	I-kappaB phosphorylation	GO_BiologicalProcess-EBI-UniProt-	1.00E-06	6.52E-05	8.01E-11	5.61E-10	[5, 6, 7, 8]	Group05	13.33	4.00	[CX3CR1, TLR2, TLR4, TNF]
GO:005090	sensory perception of taste	GO_BiologicalProcess-EBI-UniProt-	1.68E-09	1.48E-07	1.68E-09	1.01E-08	[6]	Group06	8.43	7.00	[GNAT3, TAS1R2, TAS1R3, TAS2R1, TAS2R31, TAS2R38, TAS2R43]
GO:005091	detection of chemical stimulus involved in sensory perception of taste	GO_BiologicalProcess-EBI-UniProt-	9.39E-09	7.61E-07	1.68E-09	1.01E-08	[5, 7]	Group06	10.00	6.00	[TAS1R2, TAS1R3, TAS2R1, TAS2R31, TAS2R38, TAS2R43]
GO:000158	detection of chemical stimulus involved in sensory perception of bitter taste	GO_BiologicalProcess-EBI-UniProt-	5.30E-06	2.86E-04	1.68E-09	1.01E-08	[6, 8]	Group06	8.89	4.00	[TAS2R1, TAS2R31, TAS2R38, TAS2R43]
GO:000852	taste receptor activity	GO_BiologicalProcess-EBI-UniProt-	6.37E-10	5.80E-08	1.68E-09	1.01E-08	[6, 8]	Group06	15.38	6.00	[TAS1R2, TAS1R3, TAS2R1, TAS2R31, TAS2R38, TAS2R43]
GO:005091	sensory perception of bitter taste	GO_BiologicalProcess-EBI-UniProt-	1.76E-07	1.28E-05	1.68E-09	1.01E-08	[7]	Group06	10.00	5.00	[GNAT3, TAS2R1, TAS2R31, TAS2R38, TAS2R43]
GO:005091	sensory perception of sweet taste	GO_BiologicalProcess-EBI-UniProt-	4.48E-06	2.47E-04	1.68E-09	1.01E-08	[7]	Group06	23.08	3.00	[GNAT3, TAS1R2, TAS1R3]
GO:003303	bitter taste receptor activity	GO_BiologicalProcess-EBI-UniProt-	3.93E-07	2.79E-05	1.68E-09	1.01E-08	[7, 9]	Group06	16.67	4.00	[TAS2R1, TAS2R31, TAS2R38, TAS2R43]
GO:200040	regulation of lymphocyte migration	GO_BiologicalProcess-EBI-UniProt-	1.83E-09	1.57E-07	1.16E-16	1.16E-15	[4, 5, 6, 7, 8]	Group07	8.33	7.00	[CCL2, CCL21, CCL27, CCL7, CCR2, CXCL10, XCL1]
GO:003574	natural killer cell chemotaxis	GO_BiologicalProcess-EBI-UniProt-	5.70E-06	3.02E-04	1.16E-16	1.16E-15	[5, 7, 8]	Group07	21.43	3.00	[CCL2, CCL7, XCL1]
GO:007267	eosinophil migration	GO_BiologicalProcess-EBI-UniProt-	5.94E-14	6.06E-12	1.16E-16	1.16E-15	[5, 7, 8]	Group07	20.51	8.00	[CCL11, CCL13, CCL2, CCL23, CCL7, CCL8, IL4, XCL1]
GO:004824	eosinophil chemotaxis	GO_BiologicalProcess-EBI-UniProt-	5.75E-15	6.09E-13	1.16E-16	1.16E-15	[5, 6, 7, 8, 9]	Group07	26.67	8.00	[CCL11, CCL13, CCL2, CCL23, CCL7, CCL8, IL4, XCL1]
GO:190162	regulation of lymphocyte chemotaxis	GO_BiologicalProcess-EBI-UniProt-	1.17E-12	1.17E-10	1.16E-16	1.16E-15	[5, 6, 7, 8, 9]	Group07	22.58	7.00	[CCL2, CCL21, CCL27, CCL7, CCR2, CXCL10, XCL1]
GO:014013	positive regulation of lymphocyte chemotaxis	GO_BiologicalProcess-EBI-UniProt-	5.72E-09	4.75E-07	1.16E-16	1.16E-15	[5, 6, 7, 8, 9, 1	Group07	19.23	5.00	[CCL21, CCL27, CCL7, CCR2, XCL1]
GO:200050	regulation of natural killer cell chemotaxis	GO_BiologicalProcess-EBI-UniProt-	2.60E-06	1.48E-04	1.16E-16	1.16E-15	[6, 7, 8, 9, 10]	Group07	27.27	3.00	[CCL2, CCL7, XCL1]
GO:003633	dendritic cell migration	GO_BiologicalProcess-EBI-UniProt-	1.96E-16	2.11E-14	8.21E-16	7.39E-15	[3, 5, 6]	Group08	25.00	9.00	[CCL19, CCL21, CCR1, CCR2, CCR7, CXCR1, CXCR2, CXCR4, IL12A]
GO:000240	dendritic cell chemotaxis	GO_BiologicalProcess-EBI-UniProt-	3.01E-17	3.28E-15	8.21E-16	7.39E-15	[4, 6, 7]	Group08	30.00	9.00	[CCL19, CCL21, CCR1, CCR2, CCR7, CXCR1, CXCR2, CXCR4, IL12A]
GO:000489	cytokine receptor activity	GO_BiologicalProcess-EBI-UniProt-	1.80E-18	2.04E-16	8.21E-16	7.39E-15	[5, 6, 7]	Group08	11.11	13.00	[ACKR3, CCR1, CCR10, CCR2, CCR3, CCR4, CCR7, CX3CR1, CXCR1, CXCR2, CXCR4, CXCR5, GPR17]
GO:000852	G protein-coupled peptide receptor activity	GO_BiologicalProcess-EBI-UniProt-	5.20E-16	5.57E-14	8.21E-16	7.39E-15	[5, 6, 7]	Group08	7.26	13.00	[ACKR3, CCR1, CCR10, CCR2, CCR3, CCR4, CCR7, CX3CR1, CXCR1, CXCR2, CXCR4, CXCR5, GPR17]
GO:001972	calcium-mediated signaling	GO_BiologicalProcess-EBI-UniProt-	2.77E-14	2.88E-12	8.21E-16	7.39E-15	[5, 6, 7]	Group08	4.49	14.00	[ACKR3, CCR1, CCR10, CCR2, CCR3, CCR4, CCR7, CX3CR1, CXCL8, CXCR1, CXCR2, CXCR4, CXCR5, TNF]
GO:000163	G protein-coupled chemoattractant receptor activity	GO_BiologicalProcess-EBI-UniProt-	1.17E-26	1.45E-24	8.21E-16	7.39E-15	[6, 7, 8]	Group08	40.63	13.00	[ACKR3, CCR1, CCR10, CCR2, CCR3, CCR4, CCR7, CX3CR1, CXCR1, CXCR2, CXCR4, CXCR5, GPR17]
GO:000495	chemokine receptor activity	GO_BiologicalProcess-EBI-UniProt-	1.17E-26	1.45E-24	8.21E-16	7.39E-15	[6, 7, 8, 9]	Group08	40.63	13.00	[ACKR3, CCR1, CCR10, CCR2, CCR3, CCR4, CCR7, CX3CR1, CXCR1, CXCR2, CXCR4, CXCR5, GPR17]
GO:001649	C-C chemokine receptor activity	GO_BiologicalProcess-EBI-UniProt-	9.16E-25	1.12E-22	8.21E-16	7.39E-15	[7, 8, 9, 10]	Group08	41.38	12.00	[ACKR3, CCR1, CCR10, CCR2, CCR3, CCR4, CCR7, CX3CR1, CXCR1, CXCR2, CXCR4, CXCR5]
GO:001649	C-X-C chemokine receptor activity	GO_BiologicalProcess-EBI-UniProt-	1.13E-11	1.10E-09	8.21E-16	7.39E-15	[7, 8, 9, 10]	Group08	55.56	5.00	[ACKR3, CXCR1, CXCR2, CXCR4, CXCR5]
GO:005128	positive regulation of release of sequestered calcium ion into cytosol	GO_BiologicalProcess-EBI-UniProt-	6.81E-04	4.77E-03	5.03E-07	1.51E-06	[4, 5, 6, 7, 8, 9	Group09	4.48	3.00	[CXCL10, CXCL9, XCL1]
GO:000694	syncytium formation	GO_BiologicalProcess-EBI-UniProt-	7.74E-04	3.10E-03	5.03E-07	1.51E-06	[2, 3, 4, 5]	Group09	4.29	3.00	[CXCL10, CXCL9, IL4]
GO:000076	syncytium formation by plasma membrane fusion	GO_BiologicalProcess-EBI-UniProt-	7.11E-04	4.27E-03	5.03E-07	1.51E-06	[3, 4, 5, 6]	Group09	4.41	3.00	[CXCL10, CXCL9, IL4]
GO:004566	regulation of myoblast differentiation	GO_BiologicalProcess-EBI-UniProt-	6.23E-04	5.61E-03	5.03E-07	1.51E-06	[4, 5]	Group09	4.62	3.00	[CXCL10, CXCL9, TNF]
GO:000752	myoblast fusion	GO_BiologicalProcess-EBI-UniProt-	3.61E-04	5.06E-03	5.03E-07	1.51E-06	[4, 5, 6, 7]	Group09	5.56	3.00	[CXCL10, CXCL9, IL4]
GO:006014	regulation of syncytium formation by plasma membrane fusion	GO_BiologicalProcess-EBI-UniProt-	1.26E-04	2.53E-03	5.03E-07	1.51E-06	[4, 5, 6, 7]	Group09	7.89	3.00	[CXCL10, CXCL9, IL4]
GO:001083	regulation of myotube differentiation	GO_BiologicalProcess-EBI-UniProt-	8.41E-04	2.52E-03	5.03E-07	1.51E-06	[6, 7]	Group09	4.17	3.00	[CXCL10, CXCL9, IL4]
GO:190173	regulation of myoblast fusion	GO_BiologicalProcess-EBI-UniProt-	5.00E-05	1.75E-03	5.03E-07	1.51E-06	[5, 6, 7, 8]	Group09	10.71	3.00	[CXCL10, CXCL9, IL4]
GO:200040	regulation of T cell migration	GO_BiologicalProcess-EBI-UniProt-	4.45E-07	3.11E-05	5.03E-07	1.51E-06	[5, 6, 7, 8, 9]	Group09	8.33	5.00	[CCL21, CCL27, CCR2, CXCL10, XCL1]
GO:200040	positive regulation of T cell migration	GO_BiologicalProcess-EBI-UniProt-	6.33E-08	4.94E-06	5.03E-07	1.51E-06	[5, 6, 7, 8, 9, 1	Group09	12.20	5.00	[CCL21, CCL27, CCR2, CXCL10, XCL1]
GO:001081	regulation of T cell chemotaxis	GO_BiologicalProcess-EBI-UniProt-	1.02E-09	9.22E-08	5.03E-07	1.51E-06	[6, 7, 8, 9, 10]	Group09	26.32	5.00	[CCL21, CCL27, CCR2, CXCL10, XCL1]
GO:004512	cellular extravasation	GO_BiologicalProcess-EBI-UniProt-	2.55E-06	1.48E-04	2.07E-14	1.65E-13	[3, 5, 6]	Group10	5.88	5.00	[CCL2, CCL21, CCR2, CX3CR1, TNF]
GO:006175	leukocyte adhesion to vascular endothelial cell	GO_BiologicalProcess-EBI-UniProt-	8.12E-06	4.06E-04	2.07E-14	1.65E-13	[5]	Group10	8.00	4.00	[CCL21, CCR2, CX3CR1, TNF]
GO:004542	regulation of nitric oxide biosynthetic process	GO_BiologicalProcess-EBI-UniProt-	8.41E-04	2.52E-03	2.07E-14	1.65E-13	[4, 5, 6, 7]	Group10	4.17	3.00	[CX3CR1, TLR4, TNF]
GO:005090	leukocyte tethering or rolling	GO_BiologicalProcess-EBI-UniProt-	1.69E-06	1.01E-04	2.07E-14	1.65E-13	[4, 6, 7]	Group10	11.76	4.00	[CCL21, CCR2, CX3CR1, TNF]
GO:007267	T cell migration	GO_BiologicalProcess-EBI-UniProt-	3.75E-09	3.15E-07	2.07E-14	1.65E-13	[4, 6, 7]	Group10	7.53	7.00	[CCL2, CCL21, CCL26, CCL27, CCR2, CXCL10, XCL1]
GO:190551	macrophage migration	GO_BiologicalProcess-EBI-UniProt-	5.81E-05	1.86E-03	2.07E-14	1.65E-13	[4, 6, 7]	Group10	4.88	4.00	[CCL2, CCR2, CKLF, CX3CR1]
GO:000193	negative regulation of endothelial cell proliferation	GO_BiologicalProcess-EBI-UniProt-	3.81E-04	4.96E-03	2.07E-14	1.65E-13	[5, 6, 7]	Group10	5.45	3.00	[CCL2, IL12A, TNF]
GO:200040	regulation of lymphocyte migration	GO_BiologicalProcess-EBI-UniProt-	1.83E-09	1.57E-07	2.07E-14	1.65E-13	[4, 5, 6, 7, 8]	Group10	8.33	7.00	[CCL2, CCL21, CCL27, CCL7, CCR2, CXCL10, XCL1]
GO:190499	regulation of leukocyte adhesion to vascular endothelial cell	GO_BiologicalProcess-EBI-UniProt-	7.51E-05	2.03E-03	2.07E-14	1.65E-13	[6, 7]	Group10	9.38	3.00	[CCL21, CCR2, TNF]
GO:190162	regulation of lymphocyte chemotaxis	GO_BiologicalProcess-EBI-UniProt-	1.17E-12	1.17E-10	2.07E-14	1.65E-13	[5, 6, 7, 8, 9]	Group10	22.58	7.00	[CCL2, CCL21, CCL27, CCL7, CCR2, CXCL10, XCL1]
GO:001401	positive regulation of gliogenesis	GO_BiologicalProcess-EBI-UniProt-	4.41E-06	2.47E-04	2.07E-14	1.65E-13	[5, 6, 7, 8, 9, 1	Group10	5.26	5.00	[CCR2, CX3CR1, CXCR4, TLR2, TNF]
GO:200035	regulation of endothelial cell apoptotic process	GO_BiologicalProcess-EBI-UniProt-	6.52E-04	5.22E-03	2.07E-14	1.65E-13	[7, 8]	Group10	4.55	3.00	[CCL2, IL4, TNF]
GO:007167	mononuclear cell migration	GO_BiologicalProcess-EBI-UniProt-	1.57E-25	1.93E-23	6.07E-20	7.28E-19	[3, 5, 6]	Group11	13.39	17.00	[CCL11, CCL13, CCL19, CCL2, CCL21, CCL22, CCL23, CCL26, CCL27, CCL7, CCL8, CCR1, CCR2, CXCL10, IL4, TNF, XCL1]
GO:007267	lymphocyte migration	GO_BiologicalProcess-EBI-UniProt-	7.70E-24	9.16E-22	6.07E-20	7.28E-19	[3, 5, 6]	Group11	10.76	17.00	[CCL11, CCL13, CCL19, CCL2, CCL21, CCL22, CCL23, CCL26, CCL27, CCL7, CCL8, CCR2, CCR7, CKLF, CXCL10, TAS2R38, XCL1]
GO:007055	response to interleukin-1	GO_BiologicalProcess-EBI-UniProt-	2.81E-12	2.78E-10	6.07E-20	7.28E-19	[5]	Group11	4.44	12.00	[CCL11, CCL13, CCL19, CCL2, CCL21, CCL22, CCL23, CCL26, CCL7, CCL8, CXCL8, XCL1]
GO:003434	response to interferon-gamma	GO_BiologicalProcess-EBI-UniProt-	4.69E-14	4.83E-12	6.07E-20	7.28E-19	[4, 5, 6, 7]	Group11	5.14	13.00	[CCL11, CCL13, CCL19, CCL2, CCL21, CCL22, CCL23, CCL26, CCL7, CCL8, TLR2, TLR4, XCL1]
GO:000254	monocyte chemotaxis	GO_BiologicalProcess-EBI-UniProt-	5.16E-22	6.09E-20	6.07E-20	7.28E-19	[4, 6, 7]	Group11	15.56	14.00	[CCL11, CCL13, CCL19, CCL2, CCL21, CCL22, CCL23, CCL26, CCL7, CCL8, CCR1, CCR2, CXCL10, XCL1]
GO:004824	lymphocyte chemotaxis	GO_BiologicalProcess-EBI-UniProt-	1.05E-24	1.26E-22	6.07E-20	7.28E-19	[4, 6, 7]	Group11	18.29	15.00	[CCL11, CCL13, CCL19, CCL2, CCL21, CCL22, CCL23, CCL26, CCL27, CCL7, CCL8, CCR2, CKLF, CXCL10, XCL1]
GO:007134	cellular response to interleukin-1	GO_BiologicalProcess-EBI-UniProt-	4.92E-13	4.97E-11	6.07E-20	7.28E-19	[6]	Group11	5.15	12.00	[CCL11, CCL13, CCL19, CCL2, CCL21, CCL22, CCL23, CCL26, CCL7, CCL8, CXCL8, XCL1]
GO:007134	cellular response to interferon-gamma	GO_BiologicalProcess-EBI-UniProt-	1.37E-14	1.44E-12	6.07E-20	7.28E-19	[5, 6, 7, 8]	Group11	5.65	13.00	[CCL11, CCL13, CCL19, CCL2, CCL21, CCL22, CCL23, CCL26, CCL7, CCL8, TLR2, TLR4, XCL1]
GO:003574	natural killer cell chemotaxis	GO_BiologicalProcess-EBI-UniProt-	5.70E-06	3.02E-04	6.07E-20	7.28E-19	[5, 7, 8]	Group11	21.43	3.00	[CCL2, CCL7, XCL1]
GO:007267	eosinophil migration	GO_BiologicalProcess-EBI-UniProt-	5.94E-14	6.06E-12	6.07E-20	7.28E-19	[5, 7, 8]	Group11	20.51	8.00	[CCL11, CCL13, CCL2, CCL23, CCL7, CCL8, IL4, XCL1]
GO:004824	eosinophil chemotaxis	GO_BiologicalProcess-EBI-UniProt-	5.75E-15	6.09E-13	6.07E-20	7.28E-19	[5, 6, 7, 8, 9]	Group11	26.67	8.00	[CCL11, CCL13, CCL2, CCL23, CCL7, CCL8, IL4, XCL1]
GO:200050	regulation of natural killer cell chemotaxis	GO_BiologicalProcess-EBI-UniProt-	2.60E-06	1.48E-04	6.07E-20	7.28E-19	[6, 7, 8, 9, 10]	Group11	27.27	3.00	[CCL2, CCL7, XCL1]
GO:007037	positive regulation of ERK1 and ERK2 cascade	GO_BiologicalProcess-EBI-UniProt-	9.30E-18	1.02E-15	6.07E-20	7.28E-19	[6, 7, 8, 9, 10,	Group11	5.56	16.00	[ACKR3, CCL11, CCL13, CCL19, CCL2, CCL21, CCL22, CCL23, CCL26, CCL7, CCL8, CCR1, CCR7, TLR4, TNF, XCL1]
GO:000246	dendritic cell antigen processing and presentation	GO_BiologicalProcess-EBI-UniProt-	1.50E-05	6.46E-04	6.84E-20	7.53E-19	[3]	Group12	15.79	3.00	[CCL19, CCL21, CCR7]
GO:200105	reactive nitrogen species metabolic process	GO_BiologicalProcess-EBI-UniProt-	1.21E-04	2.54E-03	6.84E-20	7.53E-19	[3]	Group12	4.04	4.00	[CX3CR1, TLR2, TLR4, TNF]
GO:000257	regulation of antigen processing and presentation	GO_BiologicalProcess-EBI-UniProt-	7.51E-05	2.03E-03	6.84E-20	7.53E-19	[3, 4]	Group12	9.38	3.00	[CCL19, CCL21, CCR7]
GO:000243	inflammatory response to antigenic stimulus	GO_BiologicalProcess-EBI-UniProt-	3.88E-05	1.43E-03	6.84E-20	7.53E-19	[3, 5]	Group12	5.41	4.00	[CCR7, CXCR2, GPR17, TNF]
GO:000257	positive regulation of antigen processing and presentation	GO_BiologicalProcess-EBI-UniProt-	8.73E-06	4.28E-04	6.84E-20	7.53E-19	[3, 4, 5]	Group12	18.75	3.00	[CCL19, CCL21, CCR7]
GO:005092	regulation of chemotaxis	GO_BiologicalProcess-EBI-UniProt-	3.44E-19	3.92E-17	6.84E-20	7.53E-19	[3, 4, 5]	Group12	5.80	17.00	[CCL19, CCL2, CCL21, CCL26, CCL27, CCL7, CCR1, CCR2, CCR4, CCR7, CXCL10, CXCL8, CXCR2, CXCR4, IL12A, IL4, XCL1]
GO:000176	establishment of lymphocyte polarity	GO_BiologicalProcess-EBI-UniProt-	8.73E-06	4.28E-04	6.84E-20	7.53E-19	[4, 5]	Group12	18.75	3.00	[CCL19, CCL21, CCR7]
GO:000177	immunological synapse formation	GO_BiologicalProcess-EBI-UniProt-	1.50E-05	6.46E-04	6.84E-20	7.53E-19	[4, 5]	Group12	15.79	3.00	[CCL19, CCL21, CCR7]
GO:000260	regulation of dendritic cell antigen processing and presentation	GO_BiologicalProcess-EBI-UniProt-	1.27E-05	5.83E-04	6.84E-20	7.53E-19	[4, 5]	Group12	16.67	3.00	[CCL19, CCL21, CCR7]
GO:003261	interleukin-10 production	GO_BiologicalProcess-EBI-UniProt-	6.09E-05	1.89E-03	6.84E-20	7.53E-19	[3, 6]	Group12	4.82	4.00	[IL4, TLR2, TLR4, XCL1]
GO:003261	interleukin-12 production	GO_BiologicalProcess-EBI-UniProt-	5.27E-05	1.74E-03	6.84E-20	7.53E-19	[3, 6]	Group12	5.00	4.00	[CCL19, CCR7, TLR2, TLR4]
GO:004620	nitric oxide metabolic process	GO_BiologicalProcess-EBI-UniProt-	1.07E-04	2.36E-03	6.84E-20	7.53E-19	[4, 5]	Group12	4.17	4.00	[CX3CR1, TLR2, TLR4, TNF]
GO:005092	positive regulation of chemotaxis	GO_BiologicalProcess-EBI-UniProt-	1.22E-20	1.42E-18	6.84E-20	7.53E-19	[3, 4, 5, 6]	Group12	8.38	16.00	[CCL19, CCL21, CCL26, CCL27, CCL7, CCR1, CCR2, CCR4, CCR7, CXCL10, CXCL8, CXCR2, CXCR4, IL12A, IL4, XCL1]
GO:003633	dendritic cell migration	GO_BiologicalProcess-EBI-UniProt-	1.96E-16	2.11E-14	6.84E-20	7.53E-19	[3, 5, 6]	Group12	25.00	9.00	[CCL19, CCL21, CCR1, CCR2, CCR7, CXCR1, CXCR2, CXCR4, IL12A]
GO:000260	positive regulation of dendritic cell antigen processing and presentation	GO_BiologicalProcess-EBI-UniProt-	1.89E-06	1.12E-04	6.84E-20	7.53E-19	[4, 5, 6]	Group12	30.00	3.00	[CCL19, CCL21, CCR7]
GO:000268	regulation of leukocyte migration	GO_BiologicalProcess-EBI-UniProt-	2.42E-19	2.78E-17	6.84E-20	7.53E-19	[3, 4, 5, 6, 7]	Group12	5.92	17.00	[CCL19, CCL2, CCL21, CCL27, CCL7, CCL8, CCR1, CCR2, CCR7, CX3CR1, CXCL10, CXCL8, CXCR2, IL12A, IL4, TNF, XCL1]
GO:003469	response to prostaglandin	GO_BiologicalProcess-EBI-UniProt-	2.24E-04	3.59E-03	6.84E-20	7.53E-19	[4, 5, 6]	Group12	6.52	3.00	[CCL19, CCL21, CCR7]
GO:004208	T-helper 1 type immune response	GO_BiologicalProcess-EBI-UniProt-	4.84E-07	3.34E-05	6.84E-20	7.53E-19	[5]	Group12	8.20	5.00	[CCL19, CCR2, IL12A, TLR4, XCL1]
GO:005176	nitric-oxide synthase biosynthetic process	GO_BiologicalProcess-EBI-UniProt-	3.11E-05	1.21E-03	6.84E-20	7.53E-19	[5]	Group12	12.50	3.00	[CCL2, TLR2, TLR4]
GO:190301	regulation of glycoprotein metabolic process	GO_BiologicalProcess-EBI-UniProt-	7.42E-04	3.71E-03	6.84E-20	7.53E-19	[4, 5, 6]	Group12	4.35	3.00	[CCL19, CCL21, CCR7]
GO:003129	lymphocyte costimulation	GO_BiologicalProcess-EBI-UniProt-	5.27E-05	1.74E-03	6.84E-20	7.53E-19	[2, 5, 6, 7, 8]	Group12	5.00	4.00	[CCL19, CCL21, CCR7, IL4]
GO:000176	establishment of T cell polarity	GO_BiologicalProcess-EBI-UniProt-	7.11E-06	3.62E-04	6.84E-20	7.53E-19	[5, 6]	Group12	20.00	3.00	[CCL19, CCL21, CCR7]
GO:000268	positive regulation of leukocyte migration	GO_BiologicalProcess-EBI-UniProt-	9.42E-21	1.10E-18	6.84E-20	7.53E-19	[3, 4, 5, 6, 7, 8	Group12	8.51	16.00	[CCL19, CCL21, CCL27, CCL7, CCL8, CCR1, CCR2, CCR7, CX3CR1, CXCL10, CXCL8, CXCR2, IL12A, IL4, TNF, XCL1]
GO:003265	regulation of interleukin-10 production	GO_BiologicalProcess-EBI-UniProt-	4.54E-05	1.63E-03	6.84E-20	7.53E-19	[4, 5, 6, 7]	Group12	5.19	4.00	[IL4, TLR2, TLR4, XCL1]
GO:003265	regulation of interleukin-12 production	GO_BiologicalProcess-EBI-UniProt-	4.54E-05	1.63E-03	6.84E-20	7.53E-19	[4, 5, 6, 7]	Group12	5.19	4.00	[CCL19, CCR7, TLR2, TLR4]
GO:005176	regulation of nitric-oxide synthase biosynthetic process	GO_BiologicalProcess-EBI-UniProt-	3.11E-05	1.21E-03	6.84E-20	7.53E-19	[5, 6]	Group12	12.50	3.00	[CCL2, TLR2, TLR4]
GO:190302	positive regulation of glycoprotein metabolic process	GO_BiologicalProcess-EBI-UniProt-	1.37E-04	2.60E-03	6.84E-20	7.53E-19	[4, 5, 6, 7]	Group12	7.69	3.00	[CCL19, CCL21, CCR7]
GO:000240	dendritic cell chemotaxis	GO_BiologicalProcess-EBI-UniProt-	3.01E-17	3.28E-15	6.84E-20	7.53E-19	[4, 6, 7]	Group12	30.00	9.00	[CCL19, CCL21, CCR1, CCR2, CCR7, CXCR1, CXCR2, CXCR4, IL12A]
GO:000240	myeloid dendritic cell chemotaxis	GO_BiologicalProcess-EBI-UniProt-	8.86E-07	5.93E-05	6.84E-20	7.53E-19	[4, 5, 6, 7, 8]	Group12	37.50	3.00	[CCL19, CCL21, CCR7]
GO:000267	regulation of acute inflammatory response	GO_BiologicalProcess-EBI-UniProt-	4.93E-04	5.91E-03	6.84E-20	7.53E-19	[5, 6, 7]	Group12	5.00	3.00	[CCR7, IL4, TNF]
GO:000268	regulation of leukocyte chemotaxis	GO_BiologicalProcess-EBI-UniProt-	5.55E-18	6.17E-16	6.84E-20	7.53E-19	[4, 5, 6, 7, 8]	Group12	8.24	14.00	[CCL19, CCL2, CCL21, CCL27, CCL7, CCR1, CCR2, CCR7, CXCL10, CXCL8, CXCR2, IL12A, IL4, XCL1]
GO:000489	cytokine receptor activity	GO_BiologicalProcess-EBI-UniProt-	1.80E-18	2.04E-16	6.84E-20	7.53E-19	[5, 6, 7]	Group12	11.11	13.00	[ACKR3, CCR1, CCR10, CCR2, CCR3, CCR4, CCR7, CX3CR1, CXCR1, CXCR2, CXCR4, CXCR5, GPR17]
GO:000852	G protein-coupled peptide receptor activity	GO_BiologicalProcess-EBI-UniProt-	5.20E-16	5.57E-14	6.84E-20	7.53E-19	[5, 6, 7]	Group12	7.26	13.00	[ACKR3, CCR1, CCR10, CCR2, CCR3, CCR4, CCR7, CX3CR1, CXCR1, CXCR2, CXCR4, CXCR5, GPR17]
GO:001055	regulation of glycoprotein biosynthetic process	GO_BiologicalProcess-EBI-UniProt-	5.17E-04	5.69E-03	6.84E-20	7.53E-19	[5, 6, 7]	Group12	4.92	3.00	[CCL19, CCL21, CCR7]
GO:003272	positive regulation of chemokine production	GO_BiologicalProcess-EBI-UniProt-	8.72E-05	2.27E-03	6.84E-20	7.53E-19	[4, 5, 6, 7, 8]	Group12	4.40	4.00	[IL4, TLR2, TLR4, TNF]
GO:003273	positive regulation of interleukin-10 production	GO_BiologicalProcess-EBI-UniProt-	9.51E-06	4.56E-04	6.84E-20	7.53E-19	[4, 5, 6, 7, 8]	Group12	7.69	4.00	[IL4, TLR2, TLR4, XCL1]
GO:003273	positive regulation of interleukin-12 production	GO_BiologicalProcess-EBI-UniProt-	1.03E-05	4.83E-04	6.84E-20	7.53E-19	[4, 5, 6, 7, 8]	Group12	7.55	4.00	[CCL19, CCR7, TLR2, TLR4]
GO:003469	response to prostaglandin E	GO_BiologicalProcess-EBI-UniProt-	9.86E-05	2.37E-03	6.84E-20	7.53E-19	[5, 6, 7]	Group12	8.57	3.00	[CCL19, CCL21, CCR7]
GO:004826	positive regulation of receptor-mediated endocytosis	GO_BiologicalProcess-EBI-UniProt-	6.81E-04	4.77E-03	6.84E-20	7.53E-19	[4, 5, 6, 7, 8]	Group12	4.48	3.00	[CCL19, CCL21, IL4]
GO:005177	positive regulation of nitric-oxide synthase biosynthetic process	GO_BiologicalProcess-EBI-UniProt-	1.50E-05	6.46E-04	6.84E-20	7.53E-19	[5, 6, 7]	Group12	15.79	3.00	[CCL2, TLR2, TLR4]
GO:009704	dendritic cell apoptotic process	GO_BiologicalProcess-EBI-UniProt-	2.60E-06	1.48E-04	6.84E-20	7.53E-19	[6]	Group12	27.27	3.00	[CCL19, CCL21, CCR7]
GO:190372	positive regulation of phospholipid metabolic process	GO_BiologicalProcess-EBI-UniProt-	7.74E-04	3.10E-03	6.84E-20	7.53E-19	[4, 5, 6, 7, 8]	Group12	4.29	3.00	[CCL19, CCL21, CCR7]
GO:003166	lipopolysaccharide-mediated signaling pathway	GO_BiologicalProcess-EBI-UniProt-	7.00E-05	1.96E-03	6.84E-20	7.53E-19	[4, 5, 6, 7, 9]	Group12	4.65	4.00	[CCL2, TLR2, TLR4, TNF]
GO:000269	positive regulation of leukocyte chemotaxis	GO_BiologicalProcess-EBI-UniProt-	4.40E-18	4.92E-16	6.84E-20	7.53E-19	[4, 5, 6, 7, 8, 9	Group12	10.40	13.00	[CCL19, CCL21, CCL27, CCL7, CCR1, CCR2, CCR7, CXCL10, CXCL8, CXCR2, IL12A, IL4, XCL1]
GO:000725	I-kappaB phosphorylation	GO_BiologicalProcess-EBI-UniProt-	1.00E-06	6.52E-05	6.84E-20	7.53E-19	[5, 6, 7, 8]	Group12	13.33	4.00	[CX3CR1, TLR2, TLR4, TNF]
GO:001056	positive regulation of glycoprotein biosynthetic process	GO_BiologicalProcess-EBI-UniProt-	1.07E-04	2.47E-03	6.84E-20	7.53E-19	[5, 6, 7, 8]	Group12	8.33	3.00	[CCL19, CCL21, CCR7]
GO:190262	regulation of neutrophil migration	GO_BiologicalProcess-EBI-UniProt-	6.85E-09	5.62E-07	6.84E-20	7.53E-19	[4, 5, 6, 7, 8, 9	Group12	10.53	6.00	[CCL19, CCL21, CCR7, CXCL8, CXCR2, XCL1]
GO:000163	G protein-coupled chemoattractant receptor activity	GO_BiologicalProcess-EBI-UniProt-	1.17E-26	1.45E-24	6.84E-20	7.53E-19	[6, 7, 8]	Group12	40.63	13.00	[ACKR3, CCR1, CCR10, CCR2, CCR3, CCR4, CCR7, CX3CR1, CXCR1, CXCR2, CXCR4, CXCR5, GPR17]
GO:004663	regulation of alpha-beta T cell activation	GO_BiologicalProcess-EBI-UniProt-	1.33E-05	5.98E-04	6.84E-20	7.53E-19	[6, 7, 8]	Group12	4.20	5.00	[CCL19, CCR2, IL12A, IL4, XCL1]
GO:007162	regulation of granulocyte chemotaxis	GO_BiologicalProcess-EBI-UniProt-	4.48E-10	4.16E-08	6.84E-20	7.53E-19	[5, 6, 7, 8, 9]	Group12	10.14	7.00	[CCL19, CCL21, CCR7, CXCL8, CXCR2, IL4, XCL1]
GO:009021	positive regulation of lipid kinase activity	GO_BiologicalProcess-EBI-UniProt-	2.55E-04	3.82E-03	6.84E-20	7.53E-19	[4, 5, 6, 7, 8, 9	Group12	6.25	3.00	[CCL19, CCL21, CCR7]
GO:009702	dendritic cell dendrite assembly	GO_BiologicalProcess-EBI-UniProt-	1.59E-07	1.18E-05	6.84E-20	7.53E-19	[7]	Group12	60.00	3.00	[CCL19, CCL21, CCR7]
GO:009702	dendritic cell differentiation	GO_BiologicalProcess-EBI-UniProt-	2.62E-05	1.05E-03	6.84E-20	7.53E-19	[5, 6, 7, 8, 9]	Group12	5.97	4.00	[CCL19, CCR7, CXCL8, IL4]
GO:190262	positive regulation of neutrophil migration	GO_BiologicalProcess-EBI-UniProt-	1.81E-09	1.57E-07	6.84E-20	7.53E-19	[4, 5, 6, 7, 8, 9	Group12	13.04	6.00	[CCL19, CCL21, CCR7, CXCL8, CXCR2, XCL1]
GO:200010	negative regulation of leukocyte apoptotic process	GO_BiologicalProcess-EBI-UniProt-	2.32E-05	9.51E-04	6.84E-20	7.53E-19	[6, 7, 8]	Group12	6.15	4.00	[CCL19, CCL21, CCR7, CXCR2]
GO:200050	regulation of dendritic cell chemotaxis	GO_BiologicalProcess-EBI-UniProt-	1.89E-06	1.12E-04	6.84E-20	7.53E-19	[5, 6, 7, 8, 9]	Group12	30.00	3.00	[CCL21, CCR7, IL12A]
GO:200054	regulation of dendritic cell dendrite assembly	GO_BiologicalProcess-EBI-UniProt-	6.37E-08	4.91E-06	6.84E-20	7.53E-19	[6, 7, 8]	Group12	75.00	3.00	[CCL19, CCL21, CCR7]
GO:000495	chemokine receptor activity	GO_BiologicalProcess-EBI-UniProt-	1.17E-26	1.45E-24	6.84E-20	7.53E-19	[6, 7, 8, 9]	Group12	40.63	13.00	[ACKR3, CCR1, CCR10, CCR2, CCR3, CCR4, CCR7, CX3CR1, CXCR1, CXCR2, CXCR4, CXCR5, GPR17]
GO:007162	positive regulation of granulocyte chemotaxis	GO_BiologicalProcess-EBI-UniProt-	1.41E-11	1.35E-09	6.84E-20	7.53E-19	[5, 6, 7, 8, 9, 1	Group12	16.28	7.00	[CCL19, CCL21, CCR7, CXCL8, CXCR2, IL4, XCL1]
GO:009002	regulation of neutrophil chemotaxis	GO_BiologicalProcess-EBI-UniProt-	1.57E-09	1.40E-07	6.84E-20	7.53E-19	[5, 6, 7, 8, 9, 1	Group12	13.33	6.00	[CCL19, CCL21, CCR7, CXCL8, CXCR2, XCL1]
GO:200051	positive regulation of dendritic cell chemotaxis	GO_BiologicalProcess-EBI-UniProt-	1.33E-06	8.22E-05	6.84E-20	7.53E-19	[5, 6, 7, 8, 9, 1	Group12	33.33	3.00	[CCL21, CCR7, IL12A]
GO:200066	regulation of dendritic cell apoptotic process	GO_BiologicalProcess-EBI-UniProt-	2.60E-06	1.48E-04	6.84E-20	7.53E-19	[7, 8]	Group12	27.27	3.00	[CCL19, CCL21, CCR7]
GO:004355	regulation of phosphatidylinositol 3-kinase activity	GO_BiologicalProcess-EBI-UniProt-	9.10E-04	1.82E-03	6.84E-20	7.53E-19	[6, 7, 8, 9, 10]	Group12	4.05	3.00	[CCL19, CCL21, CCR7]
GO:004355	positive regulation of phosphatidylinositol 3-kinase activity	GO_BiologicalProcess-EBI-UniProt-	1.71E-04	3.07E-03	6.84E-20	7.53E-19	[5, 6, 7, 8, 9, 1	Group12	7.14	3.00	[CCL19, CCL21, CCR7]
GO:009002	positive regulation of neutrophil chemotaxis	GO_BiologicalProcess-EBI-UniProt-	5.40E-10	4.97E-08	6.84E-20	7.53E-19	[5, 6, 7, 8, 9, 1	Group12	15.79	6.00	[CCL19, CCL21, CCR7, CXCL8, CXCR2, XCL1]
GO:200051	regulation of CD4-positive, alpha-beta T cell activation	GO_BiologicalProcess-EBI-UniProt-	9.47E-04	9.47E-04	6.84E-20	7.53E-19	[7, 8, 9]	Group12	4.00	3.00	[CCL19, IL4, XCL1]
GO:200066	negative regulation of dendritic cell apoptotic process	GO_BiologicalProcess-EBI-UniProt-	8.86E-07	5.93E-05	6.84E-20	7.53E-19	[7, 8, 9]	Group12	37.50	3.00	[CCL19, CCL21, CCR7]
GO:001649	C-C chemokine receptor activity	GO_BiologicalProcess-EBI-UniProt-	9.16E-25	1.12E-22	6.84E-20	7.53E-19	[7, 8, 9, 10]	Group12	41.38	12.00	[ACKR3, CCR1, CCR10, CCR2, CCR3, CCR4, CCR7, CX3CR1, CXCR1, CXCR2, CXCR4, CXCR5]
GO:005070	regulation of interleukin-1 beta secretion	GO_BiologicalProcess-EBI-UniProt-	7.74E-04	3.10E-03	6.84E-20	7.53E-19	[5, 6, 7, 8, 9, 1	Group12	4.29	3.00	[CCL19, CCR7, TLR4]
GO:005128	positive regulation of release of sequestered calcium ion into cytosol	GO_BiologicalProcess-EBI-UniProt-	6.81E-04	4.77E-03	1.27E-27	1.65E-26	[4, 5, 6, 7, 8, 9	Group13	4.48	3.00	[CXCL10, CXCL9, XCL1]
GO:004233	taxis	GO_BiologicalProcess-EBI-UniProt-	5.23E-39	7.01E-37	1.27E-27	1.65E-26	[2, 3]	Group13	4.25	36.00	[ACKR3, CCL11, CCL13, CCL19, CCL2, CCL21, CCL22, CCL23, CCL26, CCL27, CCL7, CCL8, CCR1, CCR10, CCR2, CCR3, CCR4, CCR7, CKLF, CMTM
GO:004209	type 2 immune response	GO_BiologicalProcess-EBI-UniProt-	1.96E-04	3.34E-03	1.27E-27	1.65E-26	[3]	Group13	6.82	3.00	[CCR2, IL4, XCL1]
GO:200105	reactive nitrogen species metabolic process	GO_BiologicalProcess-EBI-UniProt-	1.21E-04	2.54E-03	1.27E-27	1.65E-26	[3]	Group13	4.04	4.00	[CX3CR1, TLR2, TLR4, TNF]
GO:000693	chemotaxis	GO_BiologicalProcess-EBI-UniProt-	4.80E-39	6.48E-37	1.27E-27	1.65E-26	[3, 4]	Group13	4.26	36.00	[ACKR3, CCL11, CCL13, CCL19, CCL2, CCL21, CCL22, CCL23, CCL26, CCL27, CCL7, CCL8, CCR1, CCR10, CCR2, CCR3, CCR4, CCR7, CKLF, CMTM
GO:005090	leukocyte migration	GO_BiologicalProcess-EBI-UniProt-	1.81E-33	2.37E-31	1.27E-27	1.65E-26	[2, 4, 5]	Group13	4.59	31.00	[CCL11, CCL13, CCL19, CCL2, CCL21, CCL22, CCL23, CCL26, CCL27, CCL7, CCL8, CCR1, CCR2, CCR7, CKLF, CX3CR1, CXCL1, CXCL10, CXCL3, C
GO:000226	leukocyte activation involved in inflammatory response	GO_BiologicalProcess-EBI-UniProt-	5.96E-04	5.96E-03	1.27E-27	1.65E-26	[3, 4, 5]	Group13	4.69	3.00	[IL4, TLR2, TNF]
GO:000243	inflammatory response to antigenic stimulus	GO_BiologicalProcess-EBI-UniProt-	3.88E-05	1.43E-03	1.27E-27	1.65E-26	[3, 5]	Group13	5.41	4.00	[CCR7, CXCR2, GPR17, TNF]
GO:005092	regulation of chemotaxis	GO_BiologicalProcess-EBI-UniProt-	3.44E-19	3.92E-17	1.27E-27	1.65E-26	[3, 4, 5]	Group13	5.80	17.00	[CCL19, CCL2, CCL21, CCL26, CCL27, CCL7, CCR1, CCR2, CCR4, CCR7, CXCL10, CXCL8, CXCR2, CXCR4, IL12A, IL4, XCL1]
GO:000236	cytokine production involved in immune response	GO_BiologicalProcess-EBI-UniProt-	1.09E-07	8.31E-06	1.27E-27	1.65E-26	[3, 6]	Group13	4.64	7.00	[CCR2, IL12A, IL4, TLR2, TLR4, TNF, XCL1]
GO:003260	interferon-gamma production	GO_BiologicalProcess-EBI-UniProt-	2.00E-07	1.44E-05	1.27E-27	1.65E-26	[3, 6]	Group13	4.24	7.00	[CCR2, CCR7, CXCL8, IL12A, TLR4, TNF, XCL1]
GO:003261	interleukin-10 production	GO_BiologicalProcess-EBI-UniProt-	6.09E-05	1.89E-03	1.27E-27	1.65E-26	[3, 6]	Group13	4.82	4.00	[IL4, TLR2, TLR4, XCL1]
GO:003261	interleukin-12 production	GO_BiologicalProcess-EBI-UniProt-	5.27E-05	1.74E-03	1.27E-27	1.65E-26	[3, 6]	Group13	5.00	4.00	[CCL19, CCR7, TLR2, TLR4]
GO:004620	nitric oxide metabolic process	GO_BiologicalProcess-EBI-UniProt-	1.07E-04	2.36E-03	1.27E-27	1.65E-26	[4, 5]	Group13	4.17	4.00	[CX3CR1, TLR2, TLR4, TNF]
GO:004801	receptor ligand activity	GO_BiologicalProcess-EBI-UniProt-	9.95E-25	1.20E-22	1.27E-27	1.65E-26	[3, 4, 5, 6]	Group13	4.01	25.00	[CCL11, CCL13, CCL19, CCL2, CCL21, CCL22, CCL23, CCL26, CCL27, CCL7, CCL8, CKLF, CMTM3, CXCL1, CXCL10, CXCL3, CXCL5, CXCL8, CXCL9, I
GO:005092	positive regulation of chemotaxis	GO_BiologicalProcess-EBI-UniProt-	1.22E-20	1.42E-18	1.27E-27	1.65E-26	[3, 4, 5, 6]	Group13	8.38	16.00	[CCL19, CCL21, CCL26, CCL27, CCL7, CCR1, CCR2, CCR4, CCR7, CXCL10, CXCL8, CXCR2, CXCR4, IL12A, IL4, XCL1]
GO:006032	cell chemotaxis	GO_BiologicalProcess-EBI-UniProt-	2.57E-43	3.49E-41	1.27E-27	1.65E-26	[4, 5]	Group13	7.53	33.00	[ACKR3, CCL11, CCL13, CCL19, CCL2, CCL21, CCL22, CCL23, CCL26, CCL27, CCL7, CCL8, CCR1, CCR10, CCR2, CCR3, CCR4, CCR7, CKLF, CX3CI
GO:003059	leukocyte chemotaxis	GO_BiologicalProcess-EBI-UniProt-	1.23E-37	1.64E-35	1.27E-27	1.65E-26	[3, 5, 6]	Group13	8.70	28.00	[CCL11, CCL13, CCL19, CCL2, CCL21, CCL22, CCL23, CCL26, CCL27, CCL7, CCL8, CCR1, CCR2, CCR7, CKLF, CXCL1, CXCL10, CXCL3, CXCL5, CXC
GO:003633	dendritic cell migration	GO_BiologicalProcess-EBI-UniProt-	1.96E-16	2.11E-14	1.27E-27	1.65E-26	[3, 5, 6]	Group13	25.00	9.00	[CCL19, CCL21, CCR1, CCR2, CCR7, CXCR1, CXCR2, CXCR4, IL12A]
GO:004512	cellular extravasation	GO_BiologicalProcess-EBI-UniProt-	2.55E-06	1.48E-04	1.27E-27	1.65E-26	[3, 5, 6]	Group13	5.88	5.00	[CCL2, CCL21, CCR2, CX3CR1, TNF]
GO:007167	mononuclear cell migration	GO_BiologicalProcess-EBI-UniProt-	1.57E-25	1.93E-23	1.27E-27	1.65E-26	[3, 5, 6]	Group13	13.39	17.00	[CCL11, CCL13, CCL19, CCL2, CCL21, CCL22, CCL23, CCL26, CCL27, CCL7, CCL8, CCR1, CCR2, CXCL10, IL4, TNF, XCL1]
GO:007267	lymphocyte migration	GO_BiologicalProcess-EBI-UniProt-	7.70E-24	9.16E-22	1.27E-27	1.65E-26	[3, 5, 6]	Group13	10.76	17.00	[CCL11, CCL13, CCL19, CCL2, CCL21, CCL22, CCL23, CCL26, CCL27, CCL7, CCL8, CCR2, CCR7, CKLF, CXCL10, TAS2R38, XCL1]
GO:009752	myeloid leukocyte migration	GO_BiologicalProcess-EBI-UniProt-	2.03E-32	2.60E-30	1.27E-27	1.65E-26	[3, 5, 6]	Group13	8.09	25.00	[CCL11, CCL13, CCL19, CCL2, CCL21, CCL22, CCL23, CCL26, CCL7, CCL8, CCR1, CCR2, CCR7, CKLF, CX3CR1, CXCL1, CXCL10, CXCL3, CXCL5, CX
GO:000254	chronic inflammatory response	GO_BiologicalProcess-EBI-UniProt-	3.11E-05	1.21E-03	1.27E-27	1.65E-26	[5]	Group13	12.50	3.00	[CCL11, IL4, TNF]
GO:000268	regulation of leukocyte migration	GO_BiologicalProcess-EBI-UniProt-	2.42E-19	2.78E-17	1.27E-27	1.65E-26	[3, 4, 5, 6, 7]	Group13	5.92	17.00	[CCL19, CCL2, CCL21, CCL27, CCL7, CCL8, CCR1, CCR2, CCR7, CX3CR1, CXCL10, CXCL8, CXCR2, IL12A, IL4, TNF, XCL1]
GO:000282	positive regulation of adaptive immune response	GO_BiologicalProcess-EBI-UniProt-	1.27E-06	8.02E-05	1.27E-27	1.65E-26	[4, 5, 6]	Group13	4.41	6.00	[CCL19, CCR2, IL12A, IL4, TNF, XCL1]
GO:004208	T-helper 1 type immune response	GO_BiologicalProcess-EBI-UniProt-	4.84E-07	3.34E-05	1.27E-27	1.65E-26	[5]	Group13	8.20	5.00	[CCL19, CCR2, IL12A, TLR4, XCL1]
GO:005176	nitric-oxide synthase biosynthetic process	GO_BiologicalProcess-EBI-UniProt-	3.11E-05	1.21E-03	1.27E-27	1.65E-26	[5]	Group13	12.50	3.00	[CCL2, TLR2, TLR4]
GO:006175	leukocyte adhesion to vascular endothelial cell	GO_BiologicalProcess-EBI-UniProt-	8.12E-06	4.06E-04	1.27E-27	1.65E-26	[5]	Group13	8.00	4.00	[CCL21, CCR2, CX3CR1, TNF]
GO:006184	antimicrobial humoral immune response mediated by antimicrobial peptide	GO_BiologicalProcess-EBI-UniProt-	1.38E-08	1.11E-06	1.27E-27	1.65E-26	[4, 5, 6]	Group13	6.25	7.00	[CCL13, CXCL1, CXCL10, CXCL3, CXCL5, CXCL8, CXCL9]
GO:007055	response to interleukin-1	GO_BiologicalProcess-EBI-UniProt-	2.81E-12	2.78E-10	1.27E-27	1.65E-26	[5]	Group13	4.44	12.00	[CCL11, CCL13, CCL19, CCL2, CCL21, CCL22, CCL23, CCL26, CCL7, CCL8, CXCL8, XCL1]
GO:199086	response to chemokine	GO_BiologicalProcess-EBI-UniProt-	5.04E-55	6.90E-53	1.27E-27	1.65E-26	[5]	Group13	24.19	30.00	[ACKR3, CCL11, CCL13, CCL19, CCL2, CCL21, CCL22, CCL23, CCL26, CCL7, CCL8, CCR1, CCR10, CCR2, CCR3, CCR4, CCR7, CX3CR1, CXCL1, CX
GO:003129	lymphocyte costimulation	GO_BiologicalProcess-EBI-UniProt-	5.27E-05	1.74E-03	1.27E-27	1.65E-26	[2, 5, 6, 7, 8]	Group13	5.00	4.00	[CCL19, CCL21, CCR7, IL4]
GO:000177	microglial cell activation	GO_BiologicalProcess-EBI-UniProt-	5.96E-04	5.96E-03	1.27E-27	1.65E-26	[4, 5, 6, 7]	Group13	4.69	3.00	[IL4, TLR2, TNF]
GO:000268	positive regulation of leukocyte migration	GO_BiologicalProcess-EBI-UniProt-	9.42E-21	1.10E-18	1.27E-27	1.65E-26	[3, 4, 5, 6, 7, 8	Group13	8.51	16.00	[CCL19, CCL21, CCL27, CCL7, CCL8, CCR1, CCR2, CCR7, CX3CR1, CXCL10, CXCL8, CXCR2, IL12A, IL4, TNF, XCL1]
GO:000286	regulation of inflammatory response to antigenic stimulus	GO_BiologicalProcess-EBI-UniProt-	9.86E-05	2.37E-03	1.27E-27	1.65E-26	[4, 5, 6, 7]	Group13	8.57	3.00	[CCR7, GPR17, TNF]
GO:000512	cytokine activity	GO_BiologicalProcess-EBI-UniProt-	4.83E-31	6.09E-29	1.27E-27	1.65E-26	[4, 5, 6, 7]	Group13	8.11	24.00	[CCL11, CCL13, CCL19, CCL2, CCL21, CCL22, CCL23, CCL26, CCL27, CCL7, CCL8, CKLF, CMTM3, CXCL1, CXCL10, CXCL3, CXCL5, CXCL8, CXCL9, I
GO:003264	regulation of interferon-gamma production	GO_BiologicalProcess-EBI-UniProt-	1.17E-06	7.47E-05	1.27E-27	1.65E-26	[4, 5, 6, 7]	Group13	4.48	6.00	[CCR2, CCR7, IL12A, TLR4, TNF, XCL1]
GO:003265	regulation of interleukin-10 production	GO_BiologicalProcess-EBI-UniProt-	4.54E-05	1.63E-03	1.27E-27	1.65E-26	[4, 5, 6, 7]	Group13	5.19	4.00	[IL4, TLR2, TLR4, XCL1]
GO:003265	regulation of interleukin-12 production	GO_BiologicalProcess-EBI-UniProt-	4.54E-05	1.63E-03	1.27E-27	1.65E-26	[4, 5, 6, 7]	Group13	5.19	4.00	[CCL19, CCR7, TLR2, TLR4]
GO:003434	response to interferon-gamma	GO_BiologicalProcess-EBI-UniProt-	4.69E-14	4.83E-12	1.27E-27	1.65E-26	[4, 5, 6, 7]	Group13	5.14	13.00	[CCL11, CCL13, CCL19, CCL2, CCL21, CCL22, CCL23, CCL26, CCL7, CCL8, TLR2, TLR4, XCL1]
GO:004542	regulation of nitric oxide biosynthetic process	GO_BiologicalProcess-EBI-UniProt-	8.41E-04	2.52E-03	1.27E-27	1.65E-26	[4, 5, 6, 7]	Group13	4.17	3.00	[CX3CR1, TLR4, TNF]
GO:005176	regulation of nitric-oxide synthase biosynthetic process	GO_BiologicalProcess-EBI-UniProt-	3.11E-05	1.21E-03	1.27E-27	1.65E-26	[5, 6]	Group13	12.50	3.00	[CCL2, TLR2, TLR4]
GO:000236	T cell cytokine production	GO_BiologicalProcess-EBI-UniProt-	1.37E-05	6.05E-04	1.27E-27	1.65E-26	[4, 6, 7]	Group13	7.02	4.00	[CCR2, IL12A, IL4, XCL1]
GO:000240	dendritic cell chemotaxis	GO_BiologicalProcess-EBI-UniProt-	3.01E-17	3.28E-15	1.27E-27	1.65E-26	[4, 6, 7]	Group13	30.00	9.00	[CCL19, CCL21, CCR1, CCR2, CCR7, CXCR1, CXCR2, CXCR4, IL12A]
GO:000254	monocyte chemotaxis	GO_BiologicalProcess-EBI-UniProt-	5.16E-22	6.09E-20	1.27E-27	1.65E-26	[4, 6, 7]	Group13	15.56	14.00	[CCL11, CCL13, CCL19, CCL2, CCL21, CCL22, CCL23, CCL26, CCL7, CCL8, CCR1, CCR2, CXCL10, XCL1]
GO:004824	lymphocyte chemotaxis	GO_BiologicalProcess-EBI-UniProt-	1.05E-24	1.26E-22	1.27E-27	1.65E-26	[4, 6, 7]	Group13	18.29	15.00	[CCL11, CCL13, CCL19, CCL2, CCL21, CCL22, CCL23, CCL26, CCL27, CCL7, CCL8, CCR2, CKLF, CXCL10, XCL1]
GO:005090	leukocyte tethering or rolling	GO_BiologicalProcess-EBI-UniProt-	1.69E-06	1.01E-04	1.27E-27	1.65E-26	[4, 6, 7]	Group13	11.76	4.00	[CCL21, CCR2, CX3CR1, TNF]
GO:007267	T cell migration	GO_BiologicalProcess-EBI-UniProt-	3.75E-09	3.15E-07	1.27E-27	1.65E-26	[4, 6, 7]	Group13	7.53	7.00	[CCL2, CCL21, CCL26, CCL27, CCR2, CXCL10, XCL1]
GO:009753	granulocyte migration	GO_BiologicalProcess-EBI-UniProt-	5.79E-31	7.24E-29	1.27E-27	1.65E-26	[4, 6, 7]	Group13	10.63	22.00	[CCL11, CCL13, CCL19, CCL2, CCL21, CCL22, CCL23, CCL26, CCL7, CCL8, CCR7, CKLF, CXCL1, CXCL10, CXCL3, CXCL5, CXCL8, CXCL9, CXCR1, C
GO:190551	macrophage migration	GO_BiologicalProcess-EBI-UniProt-	5.81E-05	1.86E-03	1.27E-27	1.65E-26	[4, 6, 7]	Group13	4.88	4.00	[CCL2, CCR2, CKLF, CX3CR1]
GO:000193	negative regulation of endothelial cell proliferation	GO_BiologicalProcess-EBI-UniProt-	3.81E-04	4.96E-03	1.27E-27	1.65E-26	[5, 6, 7]	Group13	5.45	3.00	[CCL2, IL12A, TNF]
GO:000267	regulation of acute inflammatory response	GO_BiologicalProcess-EBI-UniProt-	4.93E-04	5.91E-03	1.27E-27	1.65E-26	[5, 6, 7]	Group13	5.00	3.00	[CCR7, IL4, TNF]
GO:000268	regulation of leukocyte chemotaxis	GO_BiologicalProcess-EBI-UniProt-	5.55E-18	6.17E-16	1.27E-27	1.65E-26	[4, 5, 6, 7, 8]	Group13	8.24	14.00	[CCL19, CCL2, CCL21, CCL27, CCL7, CCR1, CCR2, CCR7, CXCL10, CXCL8, CXCR2, IL12A, IL4, XCL1]
GO:000282	positive regulation of adaptive immune response based on somatic recombi	GO_BiologicalProcess-EBI-UniProt-	9.78E-07	6.45E-05	1.27E-27	1.65E-26	[5, 6, 7]	Group13	4.62	6.00	[CCL19, CCR2, IL12A, IL4, TNF, XCL1]
GO:003272	positive regulation of chemokine production	GO_BiologicalProcess-EBI-UniProt-	8.72E-05	2.27E-03	1.27E-27	1.65E-26	[4, 5, 6, 7, 8]	Group13	4.40	4.00	[IL4, TLR2, TLR4, TNF]
GO:003272	positive regulation of interferon-gamma production	GO_BiologicalProcess-EBI-UniProt-	5.81E-05	1.86E-03	1.27E-27	1.65E-26	[4, 5, 6, 7, 8]	Group13	4.88	4.00	[CCR2, IL12A, TLR4, TNF]
GO:003273	positive regulation of interleukin-10 production	GO_BiologicalProcess-EBI-UniProt-	9.51E-06	4.56E-04	1.27E-27	1.65E-26	[4, 5, 6, 7, 8]	Group13	7.69	4.00	[IL4, TLR2, TLR4, XCL1]
GO:003273	positive regulation of interleukin-12 production	GO_BiologicalProcess-EBI-UniProt-	1.03E-05	4.83E-04	1.27E-27	1.65E-26	[4, 5, 6, 7, 8]	Group13	7.55	4.00	[CCL19, CCR7, TLR2, TLR4]
GO:004210	positive regulation of cytokine biosynthetic process	GO_BiologicalProcess-EBI-UniProt-	9.49E-05	2.37E-03	1.27E-27	1.65E-26	[4, 5, 6, 7, 8]	Group13	4.30	4.00	[CCR2, IL4, TLR4, TNF]
GO:004253	tumor necrosis factor biosynthetic process	GO_BiologicalProcess-EBI-UniProt-	6.32E-06	3.29E-04	1.27E-27	1.65E-26	[4, 5, 6, 7, 8]	Group13	8.51	4.00	[CCR2, CX3CR1, IL4, TLR4]
GO:004826	positive regulation of receptor-mediated endocytosis	GO_BiologicalProcess-EBI-UniProt-	6.81E-04	4.77E-03	1.27E-27	1.65E-26	[4, 5, 6, 7, 8]	Group13	4.48	3.00	[CCL19, CCL21, IL4]
GO:005177	positive regulation of nitric-oxide synthase biosynthetic process	GO_BiologicalProcess-EBI-UniProt-	1.50E-05	6.46E-04	1.27E-27	1.65E-26	[5, 6, 7]	Group13	15.79	3.00	[CCL2, TLR2, TLR4]
GO:007009	chemokine-mediated signaling pathway	GO_BiologicalProcess-EBI-UniProt-	2.82E-56	3.89E-54	1.27E-27	1.65E-26	[5, 6, 7]	Group13	26.32	30.00	[ACKR3, CCL11, CCL13, CCL19, CCL2, CCL21, CCL22, CCL23, CCL26, CCL7, CCL8, CCR1, CCR10, CCR2, CCR3, CCR4, CCR7, CX3CR1, CXCL1, CX
GO:007134	cellular response to interleukin-1	GO_BiologicalProcess-EBI-UniProt-	4.92E-13	4.97E-11	1.27E-27	1.65E-26	[6]	Group13	5.15	12.00	[CCL11, CCL13, CCL19, CCL2, CCL21, CCL22, CCL23, CCL26, CCL7, CCL8, CXCL8, XCL1]
GO:007162	granulocyte chemotaxis	GO_BiologicalProcess-EBI-UniProt-	1.79E-32	2.32E-30	1.27E-27	1.65E-26	[4, 5, 6, 7, 8]	Group13	12.36	22.00	[CCL11, CCL13, CCL19, CCL2, CCL21, CCL22, CCL23, CCL26, CCL7, CCL8, CCR7, CKLF, CXCL1, CXCL10, CXCL3, CXCL5, CXCL8, CXCL9, CXCR1, C
GO:007167	regulation of mononuclear cell migration	GO_BiologicalProcess-EBI-UniProt-	2.41E-08	1.91E-06	1.27E-27	1.65E-26	[4, 5, 6, 7, 8]	Group13	8.57	6.00	[CCL27, CCR1, CCR2, CXCL10, IL4, TNF]
GO:199086	cellular response to chemokine	GO_BiologicalProcess-EBI-UniProt-	5.04E-55	6.90E-53	1.27E-27	1.65E-26	[6]	Group13	24.19	30.00	[ACKR3, CCL11, CCL13, CCL19, CCL2, CCL21, CCL22, CCL23, CCL26, CCL7, CCL8, CCR1, CCR10, CCR2, CCR3, CCR4, CCR7, CX3CR1, CXCL1, CX
GO:200040	regulation of lymphocyte migration	GO_BiologicalProcess-EBI-UniProt-	1.83E-09	1.57E-07	1.27E-27	1.65E-26	[4, 5, 6, 7, 8]	Group13	8.33	7.00	[CCL2, CCL21, CCL27, CCL7, CCR2, CXCL10, XCL1]
GO:003166	lipopolysaccharide-mediated signaling pathway	GO_BiologicalProcess-EBI-UniProt-	7.00E-05	1.96E-03	1.27E-27	1.65E-26	[4, 5, 6, 7, 9]	Group13	4.65	4.00	[CCL2, TLR2, TLR4, TNF]
GO:007122	cellular response to lipopolysaccharide	GO_BiologicalProcess-EBI-UniProt-	6.50E-12	6.37E-10	1.27E-27	1.65E-26	[5, 6, 8]	Group13	4.14	12.00	[CCL2, CX3CR1, CXCL1, CXCL10, CXCL3, CXCL5, CXCL8, CXCL9, IL12A, TLR2, TLR4, TNF]
GO:000269	positive regulation of leukocyte chemotaxis	GO_BiologicalProcess-EBI-UniProt-	4.40E-18	4.92E-16	1.27E-27	1.65E-26	[4, 5, 6, 7, 8, 9	Group13	10.40	13.00	[CCL19, CCL21, CCL27, CCL7, CCR1, CCR2, CCR7, CXCL10, CXCL8, CXCR2, IL12A, IL4, XCL1]
GO:000271	regulation of B cell mediated immunity	GO_BiologicalProcess-EBI-UniProt-	8.41E-04	2.52E-03	1.27E-27	1.65E-26	[6, 7]	Group13	4.17	3.00	[IL4, TNF, XCL1]
GO:000282	regulation of T-helper 1 type immune response	GO_BiologicalProcess-EBI-UniProt-	1.71E-04	3.07E-03	1.27E-27	1.65E-26	[6, 7]	Group13	7.14	3.00	[CCL19, CCR2, XCL1]
GO:000725	I-kappaB phosphorylation	GO_BiologicalProcess-EBI-UniProt-	1.00E-06	6.52E-05	1.27E-27	1.65E-26	[5, 6, 7, 8]	Group13	13.33	4.00	[CX3CR1, TLR2, TLR4, TNF]
GO:000800	chemokine activity	GO_BiologicalProcess-EBI-UniProt-	1.05E-35	1.39E-33	1.27E-27	1.65E-26	[5, 6, 7, 8]	Group13	29.23	19.00	[CCL11, CCL13, CCL19, CCL2, CCL21, CCL22, CCL23, CCL26, CCL27, CCL7, CCL8, CKLF, CXCL1, CXCL10, CXCL3, CXCL5, CXCL8, CXCL9, XCL1]
GO:007134	cellular response to interferon-gamma	GO_BiologicalProcess-EBI-UniProt-	1.37E-14	1.44E-12	1.27E-27	1.65E-26	[5, 6, 7, 8]	Group13	5.65	13.00	[CCL11, CCL13, CCL19, CCL2, CCL21, CCL22, CCL23, CCL26, CCL7, CCL8, TLR2, TLR4, XCL1]
GO:007167	positive regulation of mononuclear cell migration	GO_BiologicalProcess-EBI-UniProt-	2.19E-10	2.08E-08	1.27E-27	1.65E-26	[4, 5, 6, 7, 8, 9	Group13	18.18	6.00	[CCL27, CCR1, CCR2, CXCL10, IL4, TNF]
GO:190262	regulation of neutrophil migration	GO_BiologicalProcess-EBI-UniProt-	6.85E-09	5.62E-07	1.27E-27	1.65E-26	[4, 5, 6, 7, 8, 9	Group13	10.53	6.00	[CCL19, CCL21, CCR7, CXCL8, CXCR2, XCL1]
GO:190499	regulation of leukocyte adhesion to vascular endothelial cell	GO_BiologicalProcess-EBI-UniProt-	7.51E-05	2.03E-03	1.27E-27	1.65E-26	[6, 7]	Group13	9.38	3.00	[CCL21, CCR2, TNF]
GO:200040	positive regulation of lymphocyte migration	GO_BiologicalProcess-EBI-UniProt-	3.44E-09	2.92E-07	1.27E-27	1.65E-26	[4, 5, 6, 7, 8, 9	Group13	11.76	6.00	[CCL21, CCL27, CCL7, CCR2, CXCL10, XCL1]
GO:001081	T cell chemotaxis	GO_BiologicalProcess-EBI-UniProt-	3.20E-10	3.00E-08	1.27E-27	1.65E-26	[5, 7, 8]	Group13	17.14	6.00	[CCL21, CCL26, CCL27, CCR2, CXCL10, XCL1]
GO:003574	CD4-positive, alpha-beta T cell cytokine production	GO_BiologicalProcess-EBI-UniProt-	6.17E-05	1.85E-03	1.27E-27	1.65E-26	[5, 7, 8]	Group13	10.00	3.00	[IL12A, IL4, XCL1]
GO:003574	natural killer cell chemotaxis	GO_BiologicalProcess-EBI-UniProt-	5.70E-06	3.02E-04	1.27E-27	1.65E-26	[5, 7, 8]	Group13	21.43	3.00	[CCL2, CCL7, XCL1]
GO:007267	eosinophil migration	GO_BiologicalProcess-EBI-UniProt-	5.94E-14	6.06E-12	1.27E-27	1.65E-26	[5, 7, 8]	Group13	20.51	8.00	[CCL11, CCL13, CCL2, CCL23, CCL7, CCL8, IL4, XCL1]
GO:199026	neutrophil migration	GO_BiologicalProcess-EBI-UniProt-	3.72E-31	4.73E-29	1.27E-27	1.65E-26	[5, 7, 8]	Group13	12.65	21.00	[CCL11, CCL13, CCL19, CCL2, CCL21, CCL22, CCL23, CCL26, CCL7, CCL8, CCR7, CKLF, CXCL1, CXCL10, CXCL3, CXCL5, CXCL8, CXCL9, CXCR1, C
GO:000271	positive regulation of B cell mediated immunity	GO_BiologicalProcess-EBI-UniProt-	2.55E-04	3.82E-03	1.27E-27	1.65E-26	[6, 7, 8]	Group13	6.25	3.00	[IL4, TNF, XCL1]
GO:000282	positive regulation of T-helper 1 type immune response	GO_BiologicalProcess-EBI-UniProt-	5.00E-05	1.75E-03	1.27E-27	1.65E-26	[6, 7, 8]	Group13	10.71	3.00	[CCL19, CCR2, XCL1]
GO:003059	neutrophil chemotaxis	GO_BiologicalProcess-EBI-UniProt-	1.83E-32	2.36E-30	1.27E-27	1.65E-26	[5, 6, 7, 8, 9]	Group13	14.48	21.00	[CCL11, CCL13, CCL19, CCL2, CCL21, CCL22, CCL23, CCL26, CCL7, CCL8, CCR7, CKLF, CXCL1, CXCL10, CXCL3, CXCL5, CXCL8, CXCL9, CXCR1, C
GO:004253	regulation of tumor necrosis factor biosynthetic process	GO_BiologicalProcess-EBI-UniProt-	6.32E-06	3.29E-04	1.27E-27	1.65E-26	[5, 6, 7, 8, 9]	Group13	8.51	4.00	[CCR2, CX3CR1, IL4, TLR4]
GO:004663	regulation of alpha-beta T cell activation	GO_BiologicalProcess-EBI-UniProt-	1.33E-05	5.98E-04	1.27E-27	1.65E-26	[6, 7, 8]	Group13	4.20	5.00	[CCL19, CCR2, IL12A, IL4, XCL1]
GO:004824	eosinophil chemotaxis	GO_BiologicalProcess-EBI-UniProt-	5.75E-15	6.09E-13	1.27E-27	1.65E-26	[5, 6, 7, 8, 9]	Group13	26.67	8.00	[CCL11, CCL13, CCL2, CCL23, CCL7, CCL8, IL4, XCL1]
GO:007162	regulation of granulocyte chemotaxis	GO_BiologicalProcess-EBI-UniProt-	4.48E-10	4.16E-08	1.27E-27	1.65E-26	[5, 6, 7, 8, 9]	Group13	10.14	7.00	[CCL19, CCL21, CCR7, CXCL8, CXCR2, IL4, XCL1]
GO:009002	regulation of monocyte chemotaxis	GO_BiologicalProcess-EBI-UniProt-	1.26E-04	2.53E-03	1.27E-27	1.65E-26	[5, 6, 7, 8, 9]	Group13	7.89	3.00	[CCR1, CCR2, CXCL10]
GO:009702	dendritic cell differentiation	GO_BiologicalProcess-EBI-UniProt-	2.62E-05	1.05E-03	1.27E-27	1.65E-26	[5, 6, 7, 8, 9]	Group13	5.97	4.00	[CCL19, CCR7, CXCL8, IL4]
GO:190162	regulation of lymphocyte chemotaxis	GO_BiologicalProcess-EBI-UniProt-	1.17E-12	1.17E-10	1.27E-27	1.65E-26	[5, 6, 7, 8, 9]	Group13	22.58	7.00	[CCL2, CCL21, CCL27, CCL7, CCR2, CXCL10, XCL1]
GO:190262	positive regulation of neutrophil migration	GO_BiologicalProcess-EBI-UniProt-	1.81E-09	1.57E-07	1.27E-27	1.65E-26	[4, 5, 6, 7, 8, 9	Group13	13.04	6.00	[CCL19, CCL21, CCR7, CXCL8, CXCR2, XCL1]
GO:200040	regulation of T cell migration	GO_BiologicalProcess-EBI-UniProt-	4.45E-07	3.11E-05	1.27E-27	1.65E-26	[5, 6, 7, 8, 9]	Group13	8.33	5.00	[CCL21, CCL27, CCR2, CXCL10, XCL1]
GO:000288	regulation of immunoglobulin mediated immune response	GO_BiologicalProcess-EBI-UniProt-	8.41E-04	2.52E-03	1.27E-27	1.65E-26	[7, 8]	Group13	4.17	3.00	[IL4, TNF, XCL1]
GO:001401	positive regulation of gliogenesis	GO_BiologicalProcess-EBI-UniProt-	4.41E-06	2.47E-04	1.27E-27	1.65E-26	[5, 6, 7, 8, 9, 1	Group13	5.26	5.00	[CCR2, CX3CR1, CXCR4, TLR2, TNF]
GO:004663	positive regulation of alpha-beta T cell activation	GO_BiologicalProcess-EBI-UniProt-	5.02E-05	1.71E-03	1.27E-27	1.65E-26	[6, 7, 8, 9]	Group13	5.06	4.00	[CCL19, CCR2, IL12A, XCL1]
GO:007162	positive regulation of granulocyte chemotaxis	GO_BiologicalProcess-EBI-UniProt-	1.41E-11	1.35E-09	1.27E-27	1.65E-26	[5, 6, 7, 8, 9, 1	Group13	16.28	7.00	[CCL19, CCL21, CCR7, CXCL8, CXCR2, IL4, XCL1]
GO:009002	regulation of neutrophil chemotaxis	GO_BiologicalProcess-EBI-UniProt-	1.57E-09	1.40E-07	1.27E-27	1.65E-26	[5, 6, 7, 8, 9, 1	Group13	13.33	6.00	[CCL19, CCL21, CCR7, CXCL8, CXCR2, XCL1]
GO:009002	positive regulation of monocyte chemotaxis	GO_BiologicalProcess-EBI-UniProt-	3.53E-05	1.34E-03	1.27E-27	1.65E-26	[5, 6, 7, 8, 9, 1	Group13	12.00	3.00	[CCR1, CCR2, CXCL10]
GO:014013	positive regulation of lymphocyte chemotaxis	GO_BiologicalProcess-EBI-UniProt-	5.72E-09	4.75E-07	1.27E-27	1.65E-26	[5, 6, 7, 8, 9, 1	Group13	19.23	5.00	[CCL21, CCL27, CCL7, CCR2, XCL1]
GO:200035	regulation of endothelial cell apoptotic process	GO_BiologicalProcess-EBI-UniProt-	6.52E-04	5.22E-03	1.27E-27	1.65E-26	[7, 8]	Group13	4.55	3.00	[CCL2, IL4, TNF]
GO:200040	positive regulation of T cell migration	GO_BiologicalProcess-EBI-UniProt-	6.33E-08	4.94E-06	1.27E-27	1.65E-26	[5, 6, 7, 8, 9, 1	Group13	12.20	5.00	[CCL21, CCL27, CCR2, CXCL10, XCL1]
GO:000289	positive regulation of immunoglobulin mediated immune response	GO_BiologicalProcess-EBI-UniProt-	2.55E-04	3.82E-03	1.27E-27	1.65E-26	[7, 8, 9]	Group13	6.25	3.00	[IL4, TNF, XCL1]
GO:001081	regulation of T cell chemotaxis	GO_BiologicalProcess-EBI-UniProt-	1.02E-09	9.22E-08	1.27E-27	1.65E-26	[6, 7, 8, 9, 10]	Group13	26.32	5.00	[CCL21, CCL27, CCR2, CXCL10, XCL1]
GO:003031	osteoclast differentiation	GO_BiologicalProcess-EBI-UniProt-	1.56E-05	6.55E-04	1.27E-27	1.65E-26	[6, 7, 8, 9, 10]	Group13	4.07	5.00	[ACKR3, CCR1, IL4, TLR4, TNF]
GO:009002	positive regulation of neutrophil chemotaxis	GO_BiologicalProcess-EBI-UniProt-	5.40E-10	4.97E-08	1.27E-27	1.65E-26	[5, 6, 7, 8, 9, 1	Group13	15.79	6.00	[CCL19, CCL21, CCR7, CXCL8, CXCR2, XCL1]
GO:200050	regulation of natural killer cell chemotaxis	GO_BiologicalProcess-EBI-UniProt-	2.60E-06	1.48E-04	1.27E-27	1.65E-26	[6, 7, 8, 9, 10]	Group13	27.27	3.00	[CCL2, CCL7, XCL1]
GO:200051	regulation of CD4-positive, alpha-beta T cell activation	GO_BiologicalProcess-EBI-UniProt-	9.47E-04	9.47E-04	1.27E-27	1.65E-26	[7, 8, 9]	Group13	4.00	3.00	[CCL19, IL4, XCL1]
GO:001082	positive regulation of T cell chemotaxis	GO_BiologicalProcess-EBI-UniProt-	1.15E-07	8.59E-06	1.27E-27	1.65E-26	[6, 7, 8, 9, 10,	Group13	22.22	4.00	[CCL21, CCL27, CCR2, XCL1]
GO:004567	regulation of osteoclast differentiation	GO_BiologicalProcess-EBI-UniProt-	1.66E-06	1.01E-04	1.27E-27	1.65E-26	[6, 7, 8, 9, 10,	Group13	6.41	5.00	[ACKR3, CCR1, IL4, TLR4, TNF]
GO:005070	regulation of interleukin-1 beta secretion	GO_BiologicalProcess-EBI-UniProt-	7.74E-04	3.10E-03	1.27E-27	1.65E-26	[5, 6, 7, 8, 9, 1	Group13	4.29	3.00	[CCL19, CCR7, TLR4]
GO:004567	positive regulation of osteoclast differentiation	GO_BiologicalProcess-EBI-UniProt-	6.82E-05	1.98E-03	1.27E-27	1.65E-26	[6, 7, 8, 9, 10,	Group13	9.68	3.00	[ACKR3, CCR1, TNF]
GO:007037	positive regulation of ERK1 and ERK2 cascade	GO_BiologicalProcess-EBI-UniProt-	9.30E-18	1.02E-15	1.27E-27	1.65E-26	[6, 7, 8, 9, 10,	Group13	5.56	16.00	[ACKR3, CCL11, CCL13, CCL19, CCL2, CCL21, CCL22, CCL23, CCL26, CCL7, CCL8, CCR1, CCR7, TLR4, TNF, XCL1]

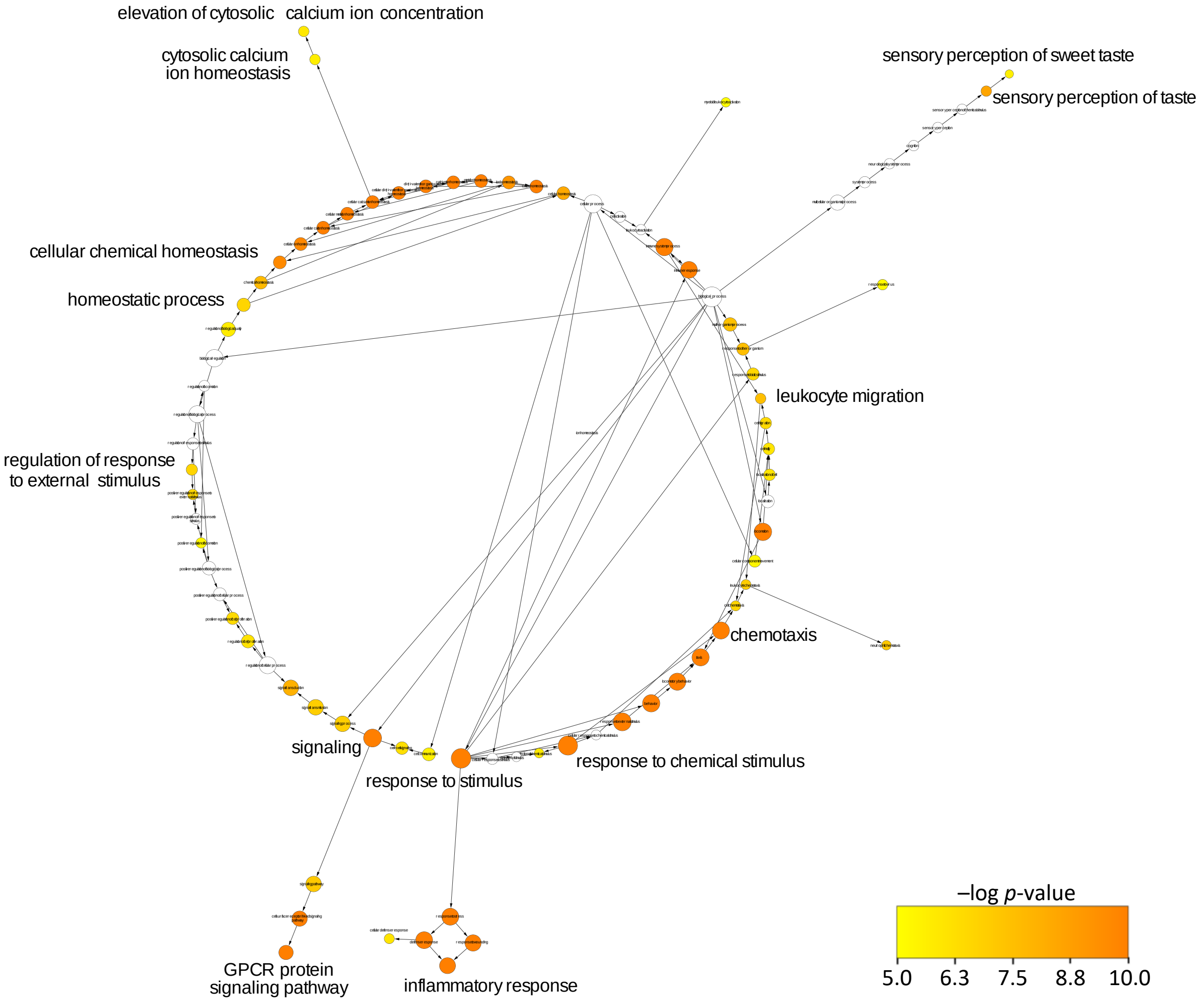

Figure S5: Bingo (v3.0.3) analysis in Cytoscape (v3.7.1) of all 48 genes (Table S4) in the sweetener mix intervention study.

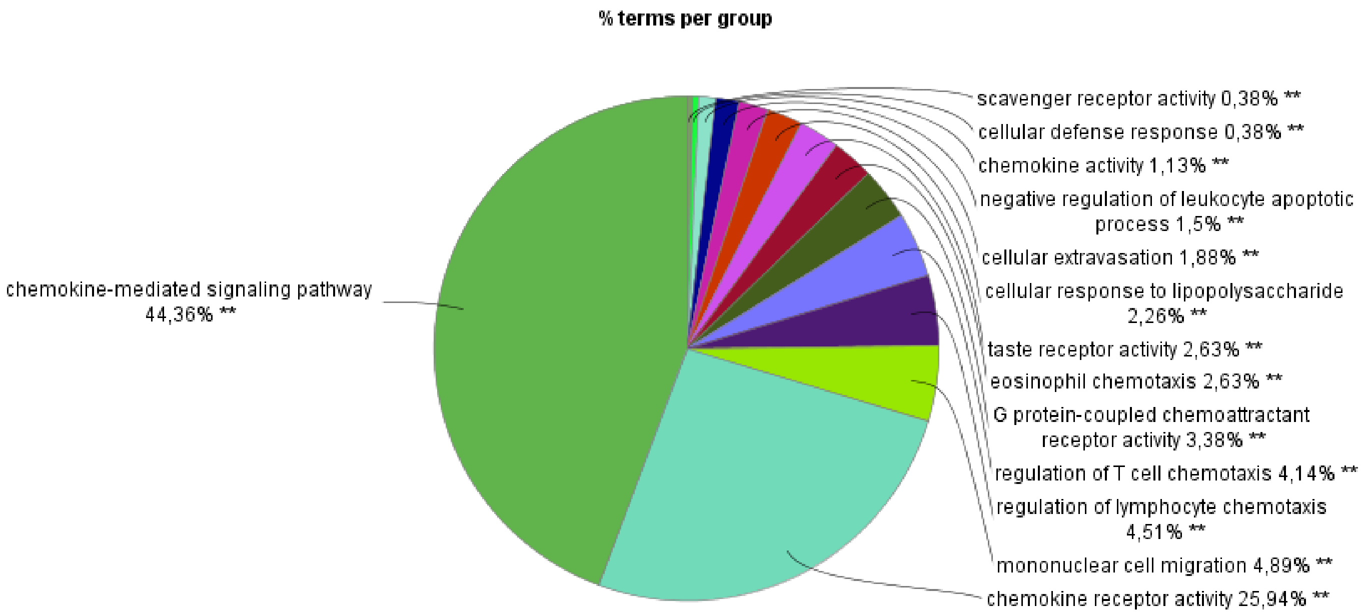

Figure S6: Pie-charts from a ClueGO GO term cluster analysis display the percentage of the number of subcategory ontology terms associated to all 48 genes analyzed in the sweetener mix intervention study. ** Bonferroni-corrected *p* value < 0.01.

References

1. Frank, O., J.K. Kreissl, A. Daschner, and T. Hofmann, *Accurate determination of reference materials and natural isolates by means of quantitative (1)h NMR spectroscopy.* J Agric Food Chem, 2014. **62**(12): p. 2506-15.
